# Understanding the Mechanism of Diels–Alder Reactions with Anionic Dienophiles: A Systematic Comparison of [ECX]^−^ (E = P, As; X = O, S, Se) Anions

**DOI:** 10.1021/acs.inorgchem.2c00549

**Published:** 2022-05-09

**Authors:** Ádám Horváth, Zoltán Benkő

**Affiliations:** Department of Inorganic and Analytical Chemistry, Budapest University of Technology and Economics, Müegyetem rkp. 3, Budapest H-1111, Hungary

## Abstract

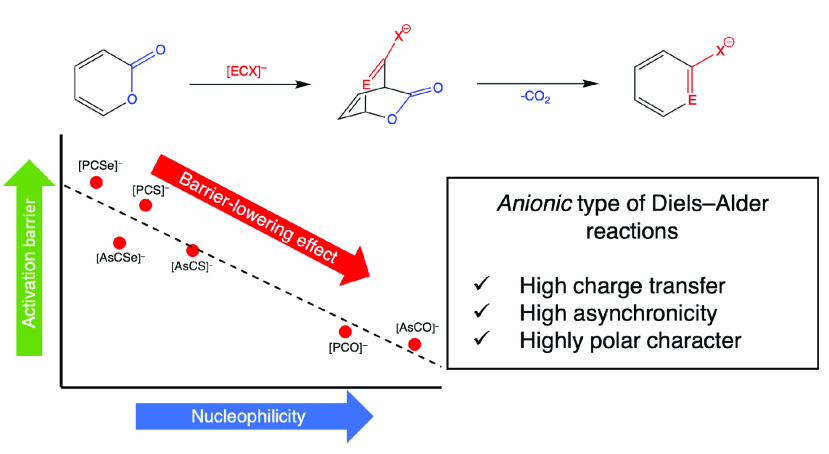

While Diels–Alder (DA) reactions involving neutral or cationic dienophiles are well-known, the characteristics of the analogous reactions with anionic dienophiles are practically unexplored. Herein we present the first comparative computational investigations on the characteristics of DA cycloadditions with anionic dienophiles on the basis of the reactions of [ECX]^−^ anions (E = P, As; X = O, S, Se) with 2*H*-pyran-2-one. All of these reactions were found to be both kinetically and thermodynamically feasible, enabling synthetic access toward 2-phosphaphenolate and arsaphenolate derivatives in the future. This study also reveals that the [ECO]^−^ anions show clear regioselectivity, while for [ECS]^−^ and [ECSe]^−^ anions, the two possible reaction channels have very similar energetics. Additionally, the activation barriers for the [ECO]^−^ anions are lower than those of the heavier analogues. The observed differences can be traced back to the starkly differing nucleophilic character of the pnictogen center in the anions, leading to a barrier-lowering effect in the case of the [ECO]^−^ anions. Furthermore, analysis of the geometries and electron distributions of the corresponding transition states revealed structure–property relationships, and thus a direct comparison of the cycloaddition reactivity of these anions was achieved. Along one of the two pathways, a good correlation was found between the activation barriers and suitable nucleophilicity descriptors (nucleophilic Parr function and global nucleophilicity). Additionally, the tendency of the reaction energies can be explained by the changing aromaticity of the products.

## Abstract 2

In contrast to the phosphaethynolate [PCO]^−^ anion, the cycloaddition reactivity of the heavier congeners ([ECX]^−^, where E = P, As and X = O, S, Se) is unexplored. In this computational study, the Diels−Alder reaction between the known [ECX]^−^ anions and 2-pyrone was employed to compare the reactivity patterns. The first activation barrier of these reactions correlates with the nucleophilicity of the anions, indicating a barrier-lowering effect. The feasibility of the studied reactions, leading to P and As heterocycles, was also explored.

## Introduction

During the past decade, the chemistry of the 2-phosphaethynolate ([PCO]^−^) anion has gained significant interest from both the experimental and theoretical points of view.^1−3^ On the basis of its first documentation by Becker et al. in 1992, this anion was synthesized in the form of a lithium salt, Li[OCP].^4^ Even though the potential of low-coordinate phosphorus compounds was clear at that time, the chemistry of the [PCO]^−^ anion remained practically unexplored for a long time, most likely because of the low stability of the lithium salt. Later on, Grützmacher and co-workers developed a convenient synthetic route to accessing the sodium salt, Na[OCP], which is stable as a dioxane adduct at room temperature and even at higher temperature under an inert atmosphere.^5^ Besides the sodium cation, further countercations have been tested; however, in practice, the sodium salt remains the most frequently used analogue, especially because of its simple and efficient synthesis in large amounts.^6−11^ Consequently, a plethora of thorough experimental and theoretical studies have been devoted to the properties and reactivity of the [PCO]^−^ anion, underlining that this simple anion has become an important building block, especially in low-coordinate phosphorus chemistry.^12−16^ On the basis of these investigations, besides nucleophilic substitutions and P^–^-transfer reactions,^13,17−22^ cycloadditions are also of high importance, in which various P heterocycles can be accessed in a straightforward manner.^12,14,23,24^

**Scheme 1:**
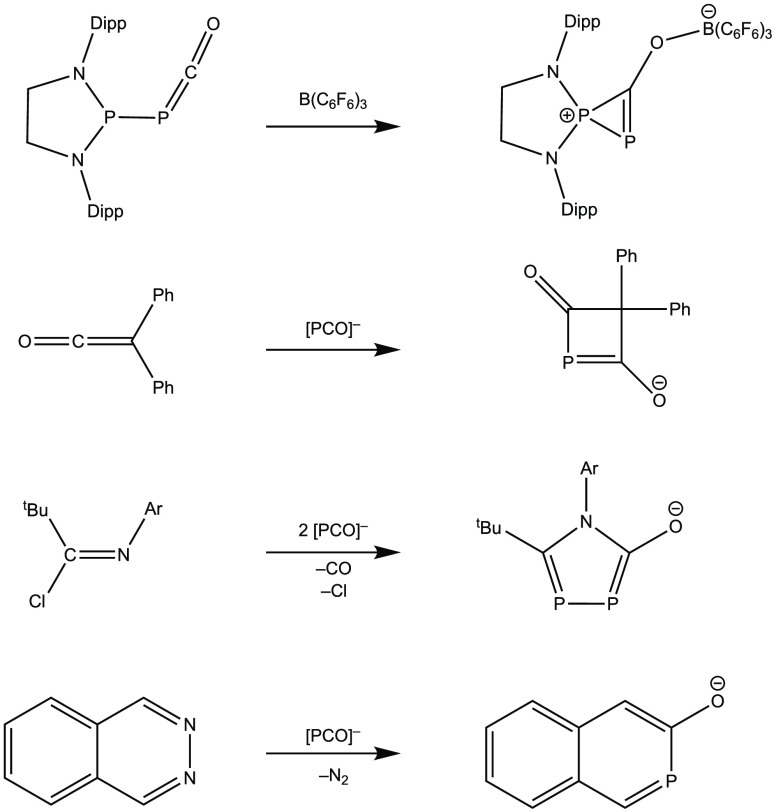
Selected Examples for Three-, Four-, Five-, and Six-Membered P Heterocycles Obtained via (Formal) Cycloadditions of the [PCO]^−^ Anion^9,20,23,27^

The first cycloadditions involving the [PCO]^−^ anion have demonstrated that it reacts with diphenylketene and a bulky carbodiimide (Dipp-N=C=N-Dipp, where Dipp = 2,6-diisopropylphenyl) in a formal [2 + 2] cycloaddition reaction, leading to four-membered heterocycles (Scheme 1).^9^ Expanding this scope, versatile [2 + 2], [3 + 2], and [4 + 2] cycloaddition reactions have been described in which the reaction partners were, e.g., tetracyclone or activated alkynes (typically with ester functionality).^1,12,25^ The experimental work also triggered computational interest in the mechanisms of these cycloaddition reactions.^26^ Recent studies reported the synthesis of aza- and diazaphosphabenzenes using tri- and tetrazines along with a phosphaphenolate analogue from methyl coumalate.^15,16^ Importantly, cycloadditions utilizing the [PCO]^−^ anion can also deliver annulations; for example, the 2-phosphanaphth-3-olate framework has been successfully accessed from phthalazine (Scheme 1).^27^ Very recently, this methodology even enabled the construction of “depolymerizable” coordination polymers.^28^ Although several heterocycles can be obtained using the [PCO]^−^ anion, these reactions typically proceed rather in a stepwise manner (via consecutive nucleophilic attacks) instead of following a concerted mechanism [such as in more conventional pericyclic reactions, e.g., Diels–Alder (DA) reactions].^14,20,26,29^

Among the heterocycles synthesized using the [PCO]^−^ anion, substituted 2-phosphaphenols and their corresponding anions (2-phosphaphenolates) are of special importance. Not only can the parent 2-phosphaphenolate anion^30,31^ be obtained from [PCO]^−^ and 2*H*-pyran-2-one but further heavily substituted analogues have also been reported, showing the functional group tolerance of this reaction.^25,32^ Importantly, these versatile 2-phosphaphenols have been utilized as ligands toward Cu^I^ and Au^I^ centers, and they also allow for the synthesis of neutral chelating ligand systems for transition metals in low oxidation states (e.g., Cu^I^, Pd^II^, and Rh^I^).^32−36^ Not surprisingly, the cycloaddition reactions of the [PCO]^−^ anion clearly resemble those of phosphaalkynes (R-C≡P), which have also become useful synthons in organophosphorus chemistry during the last decades, well-documented by the rich chemistry of the *tert*-butyl analogue, ^*t*^BuC≡P.^37−44^ The reactions of phosphaalkynes with pyrones, leading to phosphabenzenes, are also known.^45^ Recently, the cycloaddition using the trimethylsilyl analogue (TMS-C≡P) enabled synthetic access to biphosphinines (P analogues of bipyridine) and phosphaanilines.^46,47^

Several heavier congeners of the [PCO]^−^ anion are also known, however, to a much lesser extent. Among these, the [PCS]^−^ anion (the P analogue of the thiocyanate ion) was reported as a lithium salt by Becker and Hübler almost 3 decades ago.^48^ In contrast, further heavier [ECX]^−^ analogues have been achieved synthetically only very recently. Indeed, the arsaethynolate ([AsCO]^−^) anion was accessed in 2016, while the [AsCS]^−^, [AsCSe]^−^, and [PCSe]^−^ anions were obtained only in 2018 by Goicoechea and co-workers.^49,50^ While the [PCS]^−^ anion has interesting coordination properties as a ligand,^51,52^ most investigations on this anion are rather of theoretical nature (bonding analysis via computations and photoelectron spectroscopy).^51,53−57^ In general, to the best of our knowledge, the chemistry of the heavier [ECX]^−^ anions is largely unexplored experimentally, except for the [AsCO]^−^ anion, which reacts in cycloadditions with heteroallenes, such as an isocyanate (Dipp-N=C=O), a diphenylketene (Ph_2_C=C=O), and a carbodiimide (Dipp-N=C=N-Dipp).

**Scheme 2:**
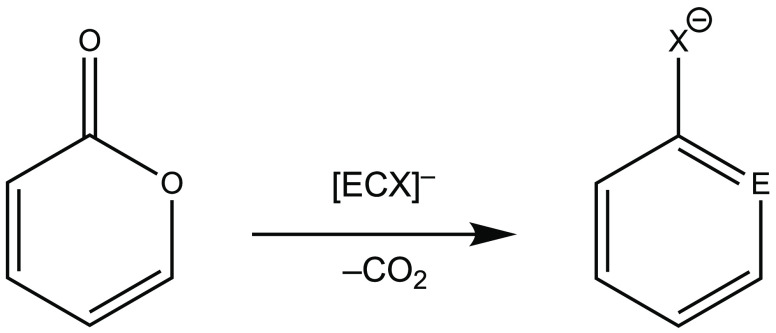
Prototype DA Cycloaddition of [ECX]^−^ Anions with 2*H*-Pyran-2-one Investigated in This Study

Being aware of the well-documented potential of the [PCO]^−^ anion in cycloadditions, we have become interested in whether similar properties can be expected for the heavier analogues as well. This has prompted us to investigate systematically the reactivity of the so-far-known P and As analogue anions with the formula [ECX]^−^ (E = P, As; X = O, S, Se) in a prototype cycloaddition with a synthetically useful diene, the 2*H*-pyran-2-one (Scheme 2). In this computational study, we have also included the neutral model species MeC≡P (Me = CH_3_ or methyl) as a reference. Our main goals are to systematically compare the cycloaddition reactivity of the investigated [ECX]^−^ anions on the basis of the activation barriers and reaction energies to establish structure–reactivity relationships. Furthermore, we target to gain a conceptual understanding of the factors influencing the energetics and regioselectivity of these model reactions using reactivity descriptors, such as global and local nucleophilicity, charge transfer, and asynchronicity. Because the outcomes of these reactions are further new phospha- and arsaphenolate analogues, our calculations may also help in the planning of the experimental work (in terms of the reaction conditions and expected regioselectivity).

## Results and Discussion

### General Description of the Reaction Profiles

**Scheme 3:**
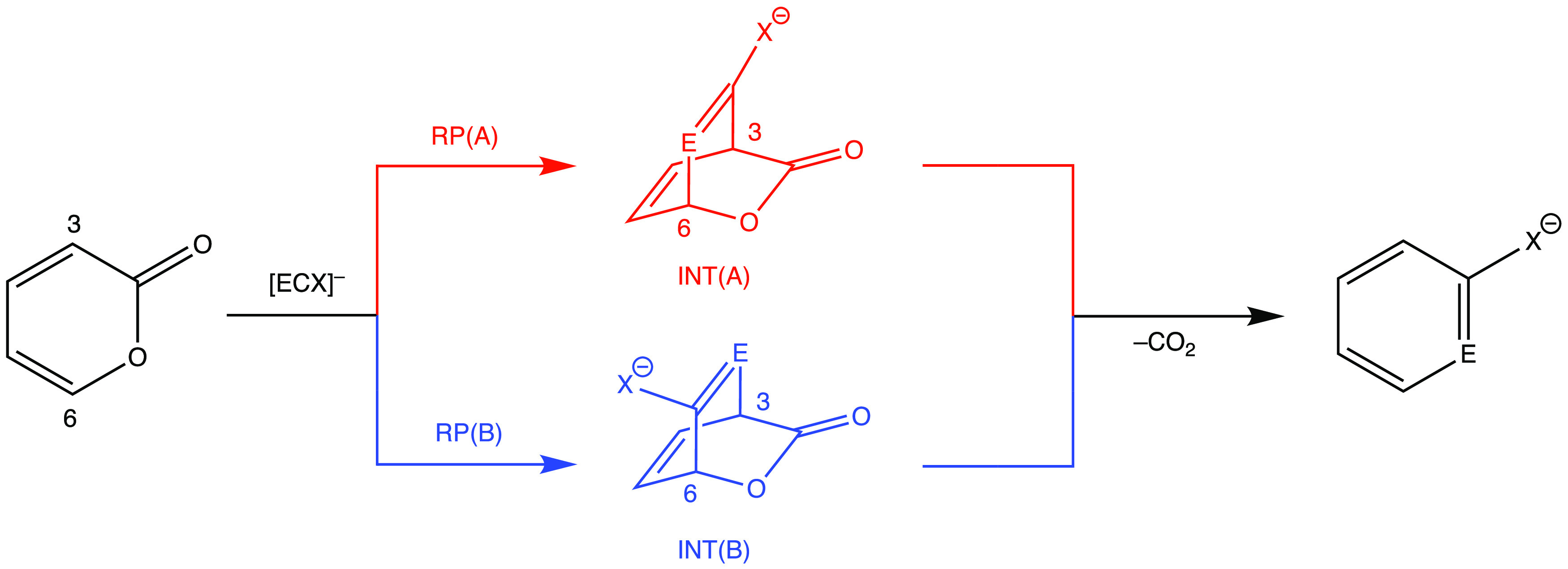
Two Possible Pathways, RP(A) (Red) and RP(B) (Blue), for the DA Reaction of [ECX]^−^ Anions with 2*H*-Pyran-2-one, Followed by a rDA Step, Leading to CO_2_ and an Anionic Pnictaphenolate Derivative

The reaction of 2-pyrone with an [ECX]^−^ anion or MeC≡P can take place via two different reaction pathways (RPs), leading to constitutional isomeric intermediates INT(A) and INT(B) (Scheme 3). In the following, we will refer to these two pathways as RP(A) and RP(B). In RP(A) (Scheme 3, red), the pnictogen heteroatom (E = P, As) attacks the C6 atom of 2-pyrone (next to the O atom in the ring), while in RP(B) (Scheme 3, blue), the C3 center next to the carbonyl group is attacked by the pnictogen center. (Additionally, for each of RP(A) and RP(B), a further pathway can be considered, which is in an enantiomeric relationship with those depicted in Scheme 3; therefore, these channels do not affect the energetics of the reactions.)

On the basis of our initial calculations, both pathways of the [PCO]^−^ anion proceed in two concerted steps (Scheme 3), which is in good accordance with a previous study.^26^ Then we extended the investigation to the other P- and As-containing [ECX]^−^ anions and MeC≡P, assuming a similar mechanism, and we located the relevant stationary points corresponding to reactant and product complexes (RC and PC, respectively), intermediates (INT), and products (PRO), as well as the transition states (TS1 and TS2) connecting them. Besides these two routes, we have explored further possibilities, in which the DA reaction is not in a concerted, but stepwise manner (that is, exclusively the pnictogen center attacks pyrone in a completely asynchronous manner); however, these remained unsuccessful after several attempts.

First, several density functional theory (DFT) and ab initio methods were evaluated; for computational details and considerations on the applied methods see the Computational Methods section. On the basis of this testing procedure, we selected the M06-2X/aug-cc-pVTZ level of theory because this gave relative energies similar to those of the DF-CCSD(T)/aug-cc-pVTZ level (the average difference:  = 3.1 kcal·mol^–1^).

**Figure 1:**
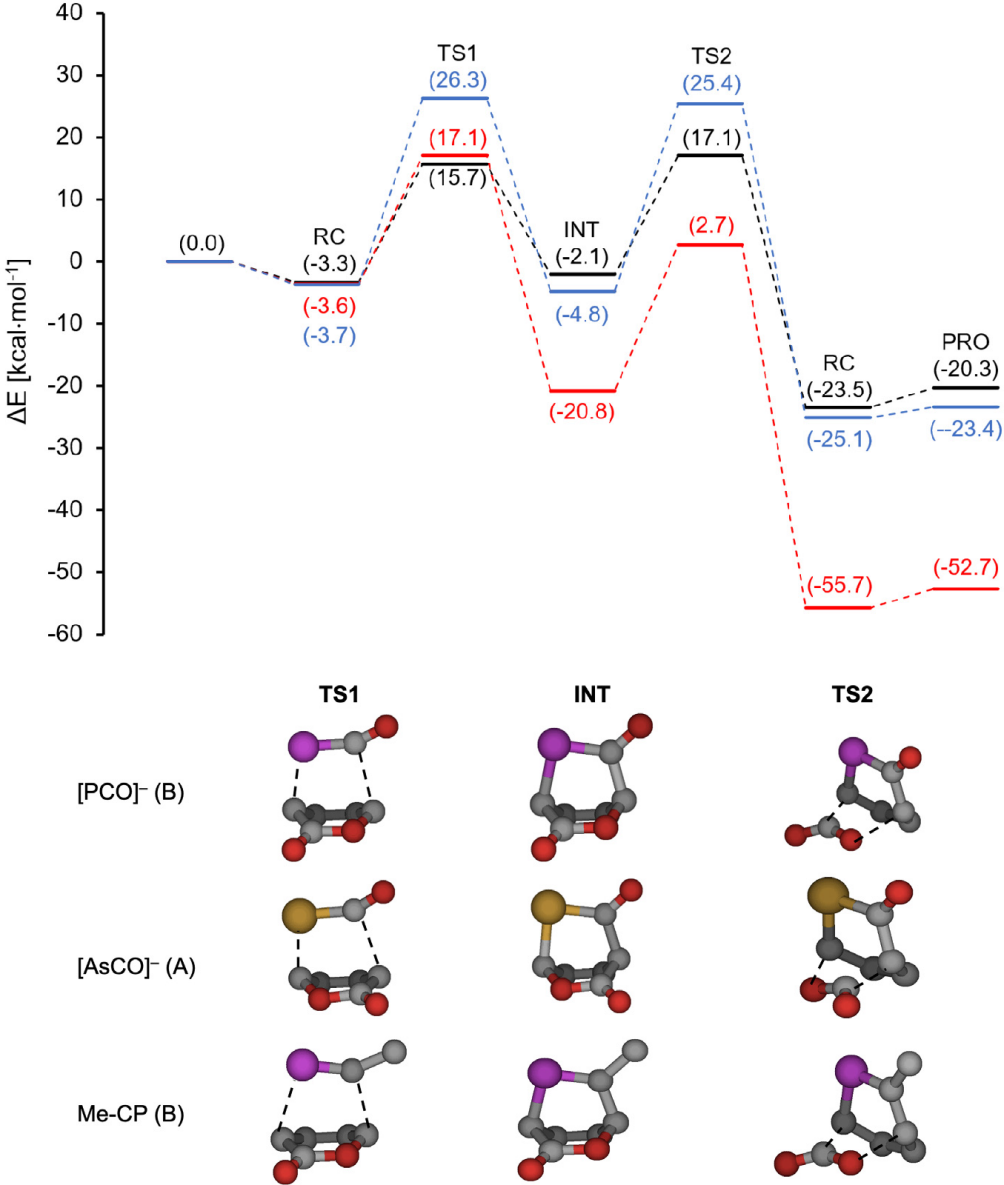
Selected sample minimum energy reaction profiles employing PCM with the notations used in the heading of Table 1 and Scheme 3 as well as the graphical presentations of TS1, INT, and TS2: C, gray; O, red, P; magenta; As, yellow. The blue, black, and red curves present the RP(B) profile for [PCO]^−^, RP(A) for [AsCO]^−^, and RP(B) for MeC≡P, respectively.

The relative energies of the stationary points belonging to the two-step RPs are presented in Table 1 (for selected reaction profiles, see Figure 1), and the corresponding Gibbs free energy profiles can be found in Table S1. Because our main goal is a comparison of the [ECX]^−^ anions and the entropy effects are very similar for the investigated reactions, in the following, we discuss the relative (electronic) energies instead of the Gibbs free energies (as is commonly employed in the literature of DA reactions^58−61^). The polarizable continuum model (PCM) calculations were carried out using tetrahydrofuran (THF) as the solvent because THF (among other ethereal solvents such as dimethoxy ethane or dioxane) is commonly employed as a moderately polar reaction medium for Na[OCP].^6,12,25,32,51^ Because THF is a donor-type solvent, in solution, most likely separated ion pairs are present; thus, countercations were not taken into account in the computations.

**Table 1:** Calculated Relative Energies Compared to the Separated Reactants (Dienophile + 2-Pyrone) for Both Steps along RP(A) and RP(B) (kcal·mol^–1^; Figure 1)^a^

dienophile (pathway)	RC	TS1	INT	TS2	PC	PRO (ΔE_rxn_)	Δ*E*_1_^⧧^	Δ*E*_2_^⧧^
[PCO]^−^ (A)	**–14.9** *–3.4*	**4.1***16.2*	**–14.0** *–1.5*	**–0.4***14.5*	**–39.4***–26.9*	**–31.4** *–23.4*	**19.0***16.2*	**13.6***16.0*
[PCO]^−^ (B)	**–15.8***–3.7*	**12.7** *26.3*	**–16.7***–4.8*	**11.4** *25.4*	**–36.8** *–25.1*		**28.5** *26.3*	**28.1** 30.2
[PCS]^−^ (A)	**–15.3***–3.9*	**10.0***21.2*	**–18.7***–7.6*	**–0.9***12.4*	**–46.5***–38.6*	**–40.4***–35.5*	**25.3***21.2*	**17.8** 20.0
[PCS]^−^ (B)	**–13.9***–4.2*	**12.0***22.9*	**–23.4***–12.1*	**1.9***14.1*	**–45.2***–36.1*		**25.9***22.9*	**25.3** 26.1
[PCSe]^−^ (A)	**–13.5***–4.1*	**11.4***22.1*	**–20.6***–10.0*	**–1.5***11.4*	**–49.6***–42.6*	**–43.7***–39.6*	**24.9***22.1*	**19.1***21.4*
[PCSe]^−^ (B)	**–13.5***–4.1*	**10.8***21.0*	**–25.6***–14.6*	**–1.3***10.4*	**–49.6***–42.6*		**24.2***21.0*	**24.3***25.1*
[AsCO]^−^ (A)	**–14.3***–3.3*	**4.1***15.7*	**–14.3***–2.1*	**2.4***17.1*	**–34.1***–23.5*	**–29.1***–20.3*	**18.4***15.7*	**16.6***19.2*
[AsCO]^−^ (B)	**–14.9***–4.1*	**10.1***23.0*	**–18.5***–7.1*	**11.5***25.8*	**–34.5***–23.1*		**25.0***23.0*	**30.0***32.9*
[AsCS]^−^ (A)	**–13.6***–4.3*	**9.0***19.4*	**–19.5***–9.3*	**1.0***13.3*	**–45.2***–37.1*	**–39.2***–34.0*	**22.5***19.4*	**20.4***22.5*
[AsCS]^−^ (B)	**–14.9***–4.1*	**9.2***19.7*	**–25.6***–15.1*	**–1.0***12.8*	**–44.0***–36.5*		**24.0***19.7*	**26.6***27.9*
[AsCSe]^−^ (A)	**–13.3***–3.1*	**10.8***19.7*	**–21.6***–11.9*	**0.2***12.0*	**–48.6***–41.7*	**–42.7***–38.6*	**24.1***19.7*	**21.7***23.9*
[AsCSe]^−^ (B)	**–13.3***–4.3*	**8.0***18.0*	**–28.0***–17.8*	**–2.5***8.7*	**–47.3***–41.0*		**21.3***18.0*	**25.4***26.5*
P≡C–Me (A)	**–5.7***–3.6*	**15.1***17.9*	**–21.8***–17.6*	**2.4***7.2*	**–55.8***–55.3*	**–57.0***–52.7*	**15.1***17.9*	**24.2***24.7*
**P≡C–Me (B)**	**–5.7***–3.6*	**15.1***17.1*	**–24.5***–20.8*	**–0.9***2.7*	**–55.8***–55.7*		**15.1***17.1*	**23.7***23.5*

a The values in boldface and italics present the DF-CCSD(T)/aug-cc-pVTZ and M06-2X/aug-cc-pVTZ(PCM=THF) levels, respectively. Δ*E*_1_^⧧^ and Δ*E*_2_^⧧^ represent the absolute activation barriers of the first and second steps, respectively (for details, see the main text).

In general, the results obtained in a vacuum (without solvent effects) and employing PCM follow similar trends, but the actual relative energies differ remarkably. These differences can be attributed to the differing strengths of the interactions (in the absence or presence of the shielding effect of the solvent). In the case of PCM calculations, the RCs have only moderate relative stability ranging from Δ*E*_rel_ = −3 to −4 kcal·mol^–1^, and thus the formation of these complexes is thermodynamically disfavored (Δ*G*_rel_ > 0 kcal·mol^–1^; Table S2); therefore, the activation energies are calculated relative to the separated reactants. In contrast, the RCs have remarkable stability in a vacuum (Δ*E*_rel_ = −14 to −16 kcal·mol^–1^), overcompensating for the effect of the entropy change; therefore, the formation of the RCs becomes thermodynamically favored (Δ*G*_rel_ < 0; Table S2). Hence, in a vacuum, it is more feasible to set the zero point of the energy profiles to the RCs instead of the separated reactants, so Δ*E*_1_^⧧^ = *E*_rel_(TS1) – *E*_rel_(RC). Consequently, the first activation barriers are very similar in a vacuum and using PCM (the difference is below 4 kcal·mol^–1^; Table 1). The formation of the intermediates (INT) is exothermic in all cases; thus, the second activation barriers are relative to the energies of the intermediates. Because the results using PCM clearly give a more realistic and practically useful picture about the energetics of the reactions conducted in the solution phase (with a moderately polar solvent), in this section, we will focus on these results in detail (Table 1).

The energy profiles of three selected examples are presented in Figure 1. Besides the neutral reference dienophile (MeC≡P, red), we also highlight the two extremes of the profiles involving anionic species: those with the highest and lowest Δ*E*_1_^⧧^ barriers, which are RP(B) of [PCO]^−^ and RP(A) of [AsCO]^−^, respectively. Because both extremes are considered, all of the remaining profiles can be placed between these two.

The first activation barriers (Δ*E*_1_^⧧^) encompass a rather wide range between 15.7 and 26.3 kcal·mol^–1^, and both reactions involving MeC≡P have rather similar barriers (Δ*E*_1_^⧧^ ≈ 17 kcal·mol^–1^). Importantly, the first transition state (TS1) determines the regioselectivity of these reactions, that is, whether the RP(A) or RP(B) path is favored kinetically. When the two first barriers (Δ*E*_1_^⧧^) are compared, the most significant regioselectivity can be observed in the cases of O-containing [PCO]^−^ and [AsCO]^−^ anions, for which the RP(A) values are favored by 10.1 and 7.3 kcal·mol^–1^, respectively. In contrast, in all of the remaining cases ([PCS]^−^, [PCSe]^−^, [AsCS]^−^ and [AsCSe]^−^ anions and the neutral MeC≡P), this selectivity is of less importance because the differences between the corresponding first barriers along the two pathways are less significant (0.3–1.7 kcal·mol^–1^). Furthermore, in the case of Se analogues [ECSe]^−^, the kinetically preferred route is RP(B). Because of its importance, the detailed analysis of the first barriers can be found in a separate section below.

The TS1s are followed by bicyclic intermediates (INT), which are more stable along RP(B) than along RP(A). Furthermore, the stability of the intermediates is rather different for the neutral and anionic dienophiles. While the neutral intermediates derived from MeC≡P have rather high relative stability (Δ*E*_rel_= −17.6/–20.8 kcal·mol^–1^), the analogues formed from the [PCO]^−^ and [AsCO]^−^ anions are less stable (e.g., Δ*E*_rel_ = −4.8 and −2.1 kcal·mol^–1^, respectively; Figure 1). Compared to the latter, the S- and Se-containing intermediates are significantly more stable: The stability of the intermediates clearly increases in the order X = O < S < Se, and it also depends slightly on the type of pnictogen; the As-containing intermediates are more stable than those with P (by 0.6–3.2 kcal·mol^–1^). As a consequence, the most stable anionic intermediates correspond to the “heaviest” [AsCSe]^−^ anion [Δ*E*_rel_= −17.8 kcal·mol^–1^ along RP(B)], being almost as stable as the intermediates obtained from the neutral MeC≡P.

In the second reaction step, the bridged bicyclic intermediates undergo a retro-Diels–Alder (rDA) reaction, leading to anionic pnictaphenolate analogues accompanied by the deliberation of carbon dioxide (CO_2_). The relative energies of the corresponding TSs are mostly rather similar but differ remarkably in two cases ([PCO]^−^ and [AsCO]^−^) along the two pathways. In most cases, the second transition state (TS2) lies at lower energy than TS1 (the exceptions are both pathways of [AsCO]^−^). However, because the intermediates are more stable than the separated reagents, in most cases the second barrier (Δ*E*_2_^⧧^) typically exceeds the first one (Δ*E*_1_^⧧^). Nevertheless, if the entropy factors are also taken into account, clearly the first step can be considered to be rate-determining (larger activation Gibbs free energies; Table S2). Again, in the O < S < Se order, a trend can be observed for the Δ*EE*_2_^⧧^ barriers: in the case of RP(A), a gradual increase is observed, while for RP(B), a decrease is observed. Following TS2, tightly bound PCs of the pnictaphenolate anion and CO_2_ form and are only slightly more stable in (electronic) energy than the separated products themselves (approximately by 3 kcal·mol^–1^). All reaction sequences are highly exothermic, offering the thermodynamic driving force for the reaction.

#### Experimental Considerations

On the basis of these reaction profiles and the available experimental data on the [PCO]^−^ anion, predictions can be targeted regarding the heavier analogues. The rate-determining barrier for the [PCO]^−^ anion is only slightly higher than 15 kcal·mol^–1^ [in the favored RP(A)], being in nice agreement with the observation that this reaction proceeds at room temperature or using slight warming.^12,25,32,62^ Furthermore, the analogous reaction of the phosphaalkyne, ^*t*^BuC≡P, was carried out at 120 °C.^38^ Additionally, we calculated the first activation barriers for the reaction of ^*t*^BuC≡P with pyrone, which were found to be 18.7 and 17.4 kcal·mol^–1^ for RP(A) and RP(B), respectively [at the M06-2X/aug-cc-pVTZ(PCM=THF) level of theory]. This in in line with the higher experimental reaction temperature (120 °C).

Because the activation barrier for the favored RP(A) path of the [AsCO]^−^ anion (15.7 kcal·mol^–1^) is rather similar to that of [PCO]^−^, the reaction of the former can be expected to take place under similar conditions. Compared to the [ECO]^−^ anions, the reactions of the remaining S- and Se-containing anions are expected to proceed at significantly higher temperature because of the considerably higher barriers (18.0 to 21.0 kcal·mol^–1^). Nevertheless, the As analogues may react more easily compared to the corresponding P analogues because of their lower first barriers. In general, the rate-determining barriers are just slightly higher than those for the ^*t*^BuC≡P analogue; therefore, at an appropriate temperature, all of these anionic heterocycles can be considered to be accessible, employing any member of the [ECX]^−^ family.

The DA reactions of the [PCO]^−^ and [AsCO]^−^ anions are highly regioselective; however, for the parent 2-pyrone, this is only of “theoretical” nature because both RPs result in the same pnictaphenolate anion. Using substituted 2-pyrones and the [PCO]^−^ ion, this regioselectivity has experimental evidence,^16,25^ demonstrating exclusive formation of the products in which the P center attacks the most electrophilic C6 center of 2-pyrone. This route is in our case RP(A); therefore, these findings are in accordance with the significantly lower activation barrier along this route.

Furthermore, the regioselectivity of the DA reactions has been studied extensively using Me_3_SiC≡P and a deuterium-labeled 2-pyrone^63^ or 6-halo-2-pyrones.^64^ In the case of deutero- or chloro-substituted pyrones, the formation of both regioisomers proves that the reaction channels (A and B) are competitive, being in nice agreement with our model calculation for the MeC≡P dienophile. In contrast, the sterically more demanding bromo substituent leads to the blockage of one of the reaction paths, resulting in the clean formation of only one of the isomers.

### Activation Energy Barrier of the DA Step

The most remarkable difference between the reactivity of the pnictogen-containing dienophiles can be outlined by investigating the first DA cycloaddition step. To the best of our knowledge, the characteristics of the DA reactions involving *anionic* dienophiles have not been studied until now. Likely, this can be traced back to two main reasons: On the one hand, suitable anionic dienophiles with tunable properties have become accessible only very recently (see the Introduction). On the other hand, conjugated dienes are substantially electron-rich (even those with electron-withdrawing groups); therefore, the diene–anion interactions are dominated by electrostatic repulsion between the two electron-rich fragments. Thus, these types of DA reactions are generally hard to access; therefore, this deserves a closer inspection.

Thorough investigations focusing on the description of DA reactions involving *neutral* or *cationic* dienophiles can be found in the literature.^65−69^ Classically, the DA-type cycloadditions can be understood based on the frontier molecular orbital (FMO) theory and, therefore, possible orbital interactions between the FMOs [highest occupied molecular orbital (HOMO) and lowest unoccupied molecular orbital (LUMO)] of the dienes and dienophiles should be analyzed. In the so-called normal-electron-demand (NED) DA reaction, the most significant stabilizing interaction arises between the LUMO of the dienophile and the HOMO of the diene. In contrast, if the determining interaction is between the HOMO of the dienophile and the LUMO of the diene, the DA reaction is considered to be inverse-electron demand (IED). Additionally, in the rarely observed case called the neutral DA reaction, both of the two possible HOMO–LUMO interactions are of importance and, therefore, both should be taken into account.^70^

#### Global Nucleophilicity

In the case of our investigated reactions, the HOMO of the diene (pyrone) is rather stabilized (ε_HOMO_ = −8.4 eV) and the LUMOs of the anions are substantially destabilized (ε_LUMO_ > 0 eV). On the contrary, pyrone has a LUMO energy of −1.1 eV, and the HOMO energies of the anions are in the range of ε_HOMO_ = −1.44 to −1.90 eV (Table 2). Thus, the favored orbital interaction arises between the HOMO of the anion and the LUMO of pyrone; hence, these reactions can be considered to be of the IED type. In contrast to the [ECX]^−^ anions, in the case of the neutral MeC≡P, both HOMO–LUMO interactions are substantial (IED gap, 7.6 eV; NED gap, 8.4 eV), leading to a neutral type.

**Table 2:** HOMO Energies (ε_HOMO_, eV) and Global Nucleophilicity Indices (*N*, eV) for the [ECX]^−^ Anions and MeC≡P, Calculated at the M06-2X/aug-cc-pVTZ Level of Theory

dienophile	ε_HOMO_	*N*
[PCO]^−^	–1.54	9.50
[PCS]^−^	–1.83	9.21
[PCSe]^−^	–1.90	9.14
[AsCO]^−^	–1.44	9.60
[AsCS]^−^	–1.76	9.28
[AsCSe]^−^	–1.87	9.17
P≡CMe	–8.69	2.35

**Figure 2:**
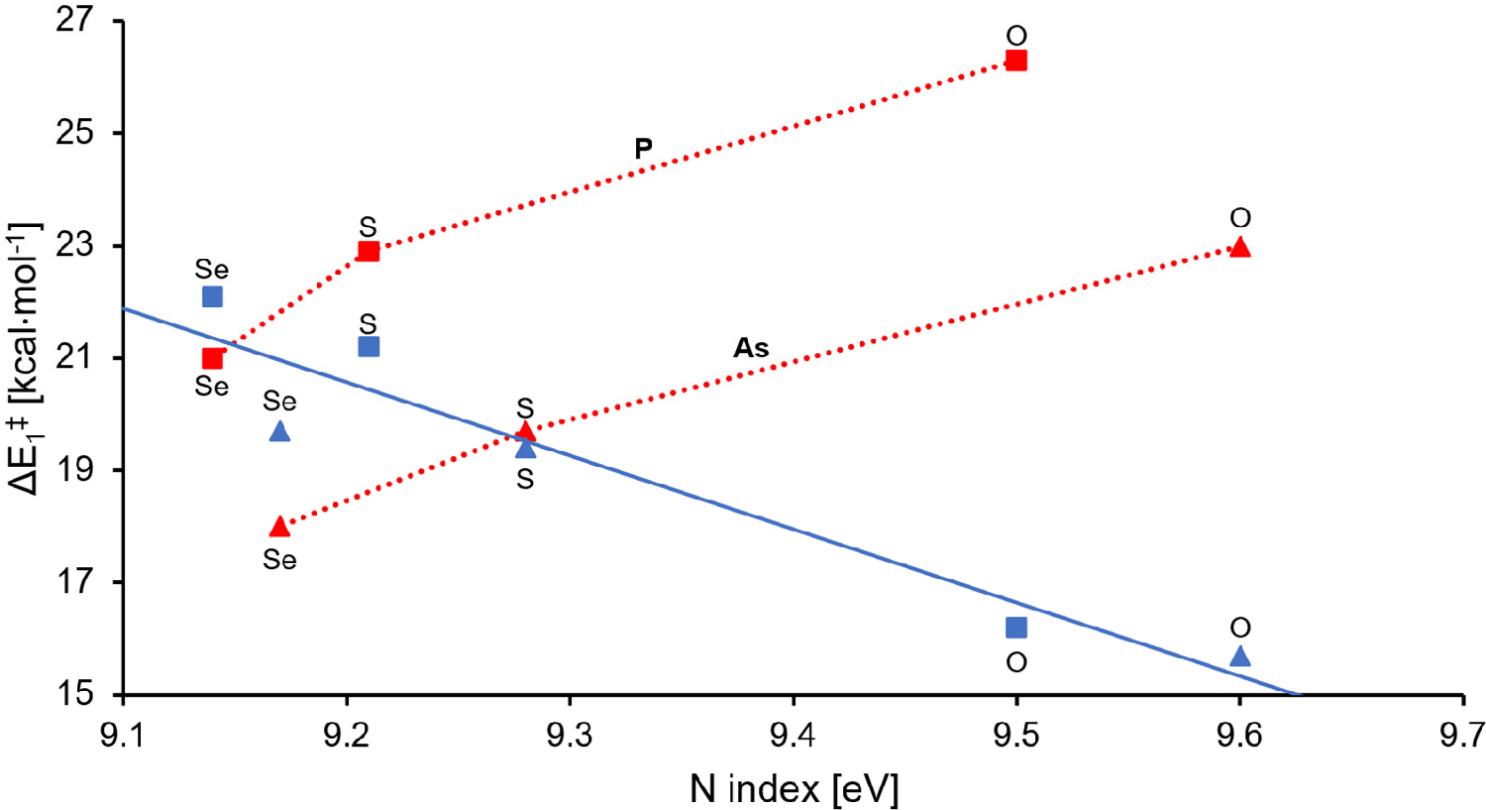
Plot of Δ*E*_1_^⧧^ versus *N* for the dienophiles. The squares and triangles represent the pnictogens P and As, respectively. The blue and red colors represent the pathways RP(A) and RP(B), respectively.

First, we discuss the effect of the HOMO energy of the dienophile on the first activation barriers. However, instead of the HOMO energies, we apply the global nucleophilicity index (*N*) introduced by Domingo et al.,^71^ which compares the HOMO energy of a given molecule to that of the reference tetracyanoethylene (see the Computational Methods section). Importantly, the pathways RP(A) and RP(B) show rather different tendencies (Figure 2).

In the case of the reactions along RP(A), the activation barriers (Δ*E*_1_^⧧^) correlate nicely with the global nucleophilicities (*R*^2^ = 0.91; Figure 2). This trend is in accordance with the change of the IED gap between the HOMO of the actual dienophile and the LUMO of 2-pyrone (the higher the HOMO energy of the anion lies, the larger the *N* index is). The decrease in the IED gap results in larger orbital stabilization, and, consequently, the global nucleophilicity of the anions has a barrier-lowering effect on the first step of RP(A). Thus, in the anionic DA reactions, the nucleophilicity of the dienophile governs the activation barriers, which complements the observations on NED DA reactions using neutral or cationic dienophiles, where the global electrophilicity parameter of the dienophile correlates with the activation barrier.^60^

In stark contrast to RP(A), the first activation barriers for the (typically unpreferred) RP(B) routes show lower variation as well as a reversed tendency. Importantly, among these points, two parallel trends can be observed depending on the pnictogen: the upper (red squares) and lower (red triangles) lines belong to the P and As analogues, respectively. Again, this separation between the pnictogen centers can be explained using the global nucleophilicity: the HOMO of the [AsCX]^−^ anions lies at slightly higher in energy compared to that of the corresponding [PCX]^−^ anions (Table 2), leading to better orbital interaction with pyrone (thus, the activation energies are lower for the As analogues). However, the nucleophilicity of the anions gives no explanation for the rising trend of the activation barriers along RP(B).

#### Further Descriptors: Parr Functions, Global and Local Charge Transfer, and Asynchronicity

**Figure 3:**
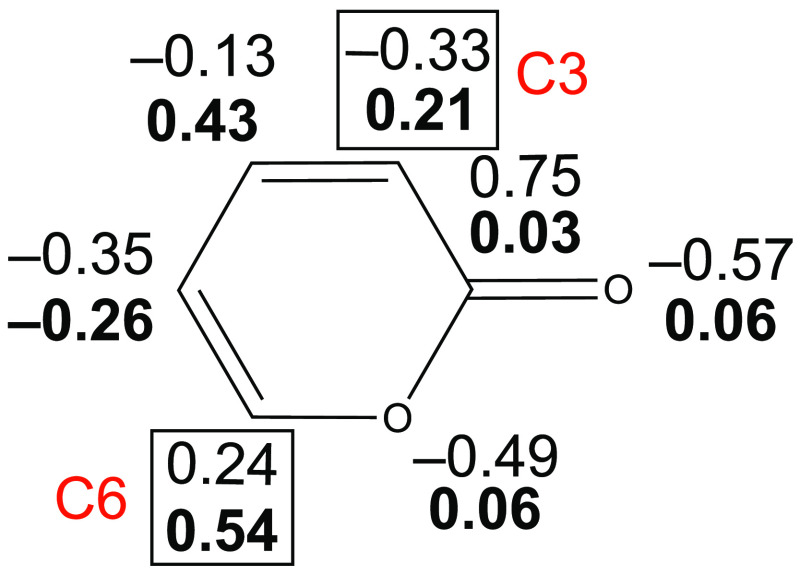
NPA partial charges (e) and electrophilic Parr functions (*P*_k_^+^, boldface) of 2*H*-pyran-2-one at the M06-2X/aug-cc-pVTZ level of theory.

Because the global nucleophilicity does not clarify the remarkable difference between the RP(A) and RP(B), the *local* properties of the reactants have to be taken into account as well. Hence, we calculated the natural population analysis (NPA) charges, as well as the nucleophilic and electrophilic Parr functions of the anions and pyrone, respectively (Table 3 and Figure 3), to account for the electrostatic and nucleophilic–electrophilic orbital interactions. In order to quantify the effects of the heteroatoms, we investigated the charge transfer (CT) and asynchronicity (*A*_sy_) in the TS1s (Table 4). In the following, we give a brief summary of the applicability of these parameters to assist the reader.

**Table 3:** NPA Partial Charges (*q*, e) and Nucleophilic Parr Function Values (*P*_k_^–^) Calculated at the M06-2X/aug-cc-pVTZ Level of Theory for the [ECX]^−^ Anions and MeC≡P

	*q*			*P*_k_^–^		
dienophile	E	C	X	E	C	X
[PCO]^−^	–0.45	0.14	–0.69	0.81	–0.04	0.23
[PCS]^−^	–0.01	–0.73	–0.26	0.69	–0.22	0.53
[PCSe]^−^	0.08	–0.83	–0.25	0.54	–0.32	0.77
[AsCO]^−^	–0.49	0.17	–0.68	0.82	0.00	0.18
[AsCS]^−^	–0.04	–0.75	–0.21	0.73	–0.15	0.42
[AsCSe]^−^	0.06	–0.87	–0.20	0.66	–0.32	0.65
P≡CMe	0.52	–0.53		0.69	0.20	

**Table 4:** Calculated Global and Local Charge-Transfer Values (CT and Δ*q*, respectively, in e) and the *A*_sy_ Indices for TS1

		Δ*q*			
dienophile (pathway)	CT	E	C	X	*A*_sy_
[PCO]^−^ (A)	0.82	0.57	0.14	0.11	0.78
[PCO]^−^ (B)	0.59	0.49	0.04	0.06	0.66
[PCS]^−^ (A)	0.64	0.37	0.08	0.19	0.81
[PCS]^−^ (B)	0.35	0.32	–0.01	0.04	0.43
[PCSe]^−^ (A)	0.55	0.31	0.06	0.18	0.80
[PCSe]^−^ (B)	0.28	0.29	–0.02	0.01	0.19
[AsCO]^−^ (A)	0.79	0.56	0.13	0.10	0.78
[AsCO]^−^ (B)	0.54	0.49	0.01	0.04	0.50
[AsCS]^−^ (A)	0.59	0.38	0.07	0.14	0.78
[AsCS]^−^ (B)	0.34	0.35	–0.02	0.01	0.33
[AsCSe]^−^ (A)	0.51	0.32	0.05	0.14	0.75
[AsCSe]^−^ (B)	0.30	0.32	–0.02	0.00	0.19
MeC≡P (A)	0.07	0.06	0.02		0.00
MeC≡P (B)	0.06	0.10	–0.03		–0.04

##### Parr Functions and NPA Charges

The Parr functions quantify the local philicity of the reactive centers and are related to the contribution of these atoms to the appropriate FMO of the molecule (for nucleophilic Parr function, HOMO; for electrophilic Parr function, LUMO).^72,73^ Therefore, the Parr functions predict the favorable local nucleophilic–electrophilic orbital interactions arising between the two reactants, and, hence, the favored orientations can be identified. In contrast, the NPA charges can be used to map the electrostatic effects.^74^

In pyrone, both reacting centers, C3 and C6, have electrophilic character (Figure 3), but C6 is clearly more electrophilic (*P*_k_^+^ = 0.54) than C3 (*P*_k_^+^ = 0.21). The partial charges on these centers are also markedly different because C6 is positively charged, while C3 is negatively charged. Among the two reacting centers of the anions (E and C), the pnictogen (E) is significantly more nucleophilic (larger *P*_k_^–^ values) than the C atom (Table 3). However, the NPA charges show a remarkable variation: For the [PCO]^−^ and [AsCO]^−^ anions, the negative charge is localized on the pnictogen center, while the C center is slightly positively charged. In contrast, in the S and Se analogues, the C centers are negatively charged and the pnictogens are practically neutral.

##### Charge Transfer

The global CT measures the transferred electron density from the anionic dienophiles to the pyrone, and therefore it is related to the strength of the nucleophilic–electrophilic orbital interactions between the reactants.^75^ The global CT can be decomposed to local CT terms (Δ*q*): CT = ∑Δ*q*. These Δ*q* values account for the transferred electron density by a certain atom and thus predict the local nucleophilic–electrophilic interactions (for details, see the Computational Methods section).

The global CT also has an important role in the classification of DA reactions, which was established for cycloadditions of cyclopentadiene with a series of substituted ethylenes and a simple iminium cation.^60^ On the basis of the CT values, three groups of DA reactions can be considered: nonpolar (CT < 0.15 e), polar (0.15 e < CT < 0.40 e), and ionic (0.40 e < CT).

##### Asynchronicity

The classical DA cycloadditions (e.g., butadiene or cyclopentadiene with ethylene) are concerted processes,^76−78^ and thus the TSs of these reactions are highly synchronous. However, in asynchronous reactions (often called one step two stage), the evolution degree of the two new bonds is remarkably different. *A*_sy_ of the TSs also offers information on the localization of the interactions. The high *A*_sy_ is typically associated with the polar or ionic nature and is the result of a nonsymmetrical substitution pattern of the diene and/or dienophile.^67,76−82^

In the following, we will discuss the Parr functions, NPA charges, and CT and *A*_sy_ parameters with respect to the RPs. First, we discuss a neutral reference (MeC≡P), for which the two activation barriers are very similar. Furthermore, the DA reactions with MeC≡P are close to the classical ones on the basis of the practically synchronous TS1 (*A*_sy_ ≈ 0) and its nonpolar character (CT ≈ 0 e). Compared to this neutral nucleophile, the reactions with anionic dienophiles show different characteristics in several aspects.

**Figure 4:**
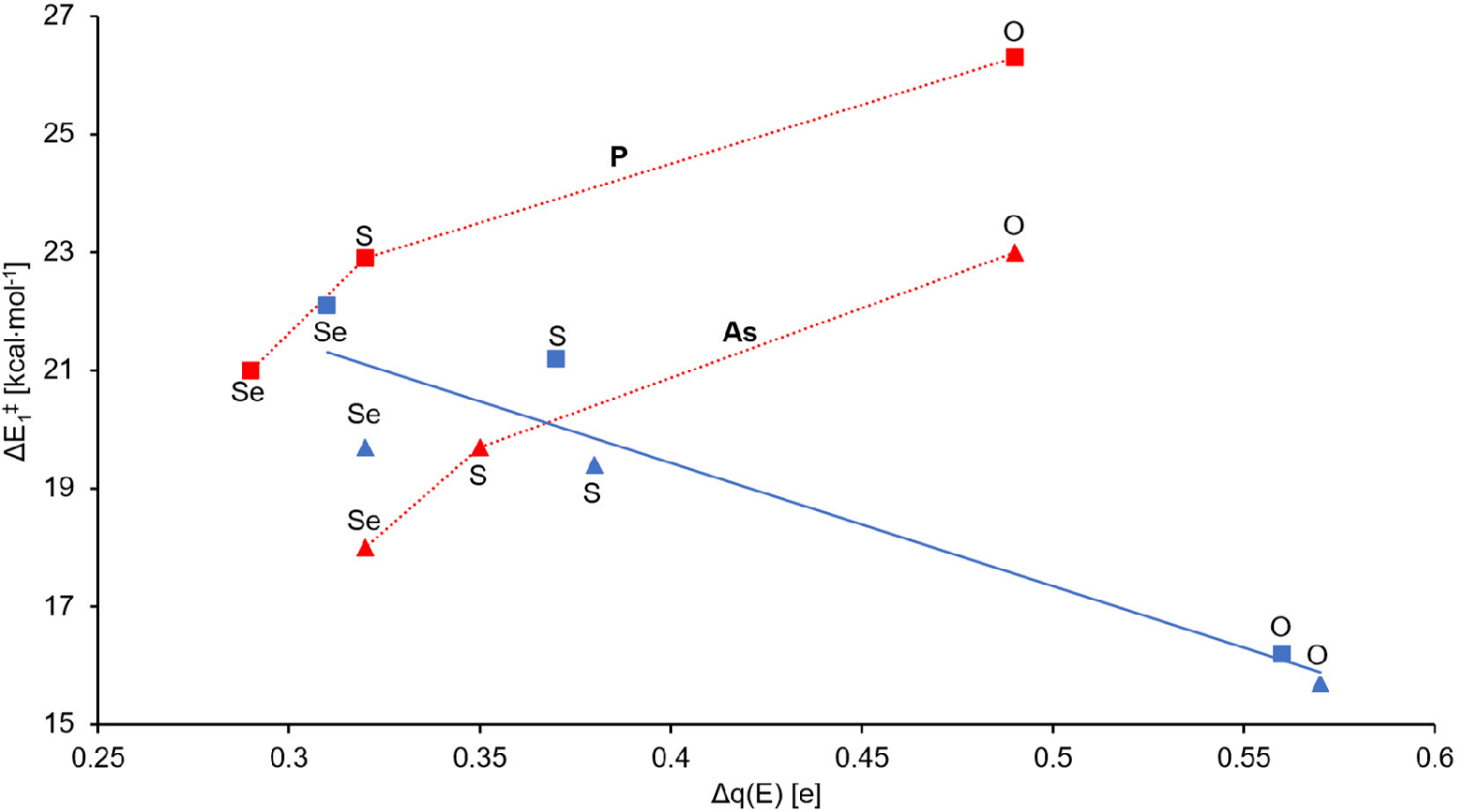
Local CT (Δ*q*) versus first activation barriers. The squares and triangles represent the pnictogens, P and As, respectively. The blue and red colors represent the pathways, RP(A) and RP(B), respectively [*R*_RP(A)_^2^ = 0.88].

If the two pathways are compared for the [ECX]^−^ anions, the Parr functions indicate a stronger local nucleophilic–electrophilic interaction when the E pnictogen center attacks the more electrophilic C6 center of pyrone, fulfilled along RP(A). Therefore, the Parr functions give a trend similar to that of the *N* indices (Figure S3). In accordance with the dominant nucleophilic–electrophilic interaction, all of the CT values are above 0.4 e; therefore, all of the DA reactions along RP(A) can be considered to be ionic.^60^ Furthermore, the transferred electron density is mainly provided by the pnictogen centers [dominant Δ*q*(E) values], showing the localized nature of the interaction between the E···C6 centers. Similar to the *N* index, a good correlation can be found between the Δ*E*_a_^⧧^ barriers and Δ*q*(E) values for the RP(A) pathways (Figure 4). Besides the local nucleophilicity, the negative charge accumulated on the pnictogen center in the [ECO]^−^ anions may also have a stabilizing effect because attractive electrostatic interactions arise between the reacting E···C6 centers. In agreement with the highly localized interactions and ionic character, the reactions are strongly asynchronous (*A*_sy_ ≈ 0.80). The high *A*_sy_ observed in the cases of the [ECO]^−^ anions is a result of the rather strong nucleophilic–electrophilic interactions; however, for the S and Se analogues, this orbital interaction is less pronounced because of the lower *P*_k_^–^ values for these anions. In these cases, the significant *A*_sy_ of the TS can be explained by the electrostatic repulsion between the C centers of the anions and the C3 center of the pyrone, which are both negatively charged (Table 3 and Figure 3).

In stark contrast to RP(A), along RP(B), the local nucleophilic–electrophilic (orbital) interactions are significantly weaker because the pnictogen centers (E) attack the much less electrophilic C3 center of the pyrone. Therefore, besides the orbital interactions, the electrostatic effects should also be taken into account for a qualitative explanation of the trend in Figures 2 and 4. Indeed, along RP(B) of the [ECO]^−^ anions, the negatively charged pnictogen center attacks the C3 center of pyrone having a negative partial charge, which results in electrostatic repulsion between these centers, destabilizing the TS for these anions. In contrast, in the S-containing and especially the Se-containing anions, the pnictogen centers are rather neutral (no repulsion between the E···C3 centers). Additionally, the C of [ECS/Se]^−^ is negatively charged, which leads to an electrostatic attraction with the C6 center of pyrone, thus stabilizing the TS. It is important to note that the differences in the activation barriers between the anions are much less significant along RP(B) than along RP(A), and the electrostatic interactions can be considered to be less dominant.

The weaker nucleophilic–electrophilic interactions along RP(B) [compared to RP(A)] can be further bolstered by the generally lower CT values and less asynchronic character of the TS structures (Table 4). The *A*_sy_ values along RP(B) encompass a wider range (0.19–0.66), which can also be traced back to the importance of both electrostatic and nucleophilic–electrophilic interactions. The CT values along RP(B) also show larger variation (0.28–0.59), indicating generally weaker interactions between the reacting centers. Furthermore, the local CT values on the pnictogen centers are also similar along both pathways, and the transferred charge is practically provided exclusively by the pnictogen (Figure 4).

### Stability of the Products and rDA Step

The final products, the pnictaphenolate analogues, are significantly more stable than the separated starting materials; thus, the reaction sequences are highly exothermic (Δ*E*_r_= −30 to −50 kcal·mol^–1^). Clearly, the formation of stable CO_2_ and aromatic products ensures the thermodynamic driving force. On the basis of the varying reaction energies, the relative stability of the pnictaphenolate derivatives (phospha- and arsaphenolates) markedly differs from that of the neutral 2-methylphosphabenzene forming from MeC≡P. While the reaction energies involving anionic species range between −29.1 and 43.7 kcal·mol^–1^, the formation of a neutral phosphabenzene is more exothermic (−57.0 kcal·mol^–1^).

These differences can be explained by the differing aromaticities of the cyclic products. In order to quantify the aromaticity, we calculated the corresponding NICS(1) values (Table 5; for NICS(0), see Table S9). 2-Methylphosphaphabenzene has a highly aromatic structure with a NICS(1) value of −9.2 ppm, which is similar to that of benzene [NICS(1) = −10.2 ppm].^83,84^ Similarly, 2-phosphaphenol also possesses significant aromatic character with a NICS(1) value of −9.5 ppm,^12^ showing that the hydroxy substituent has a negligible effect. In stark contrast, the anionic 2-pnictaphenolates are less aromatic compared to the neutral congeners. This observation is in line with the higher relative stability of the neutral 2-methylphosphabenzene (more exothermic reaction) compared to those of the anionic pnictaphenolates. Furthermore, NICS(1) shows a fair correlation (*R*^2^ = 0.77) with the reaction energies (Δ*E*_rxn_; Figure S4); thus, the reaction energies of the investigated DA reactions can serve as a simple energetic measurement for the aromaticity of these species.

**Table 5:** NICS(1) Values (ppm), Total Contribution (%) of the Aromatic and Nonaromatic Resonance Structures, Reaction Energies (Δ*E*_rxn_, kcal·mol^–1^), and HOMO Energies of the Pnictaphenolate Analogues

E	X	NICS(1)	aromatic	nonaromatic	Δ*E*_rxn_	ε_HOMO_
P	O	–4.5	26	40	–31.4	–1.9
P	S	–4.8	31	12	–40.4	–1.8
P	Se	–5.0	47	12	–43.7	–1.8
As	O	–4.4	15	35	–29.1	–1.8
As	S	–4.5	41	15	–39.2	–1.9
As	Se	–4.8	41	13	–42.7	–1.8
MeC≡P		–9.2	70		–57.0	–8.0

**Figure 5:**
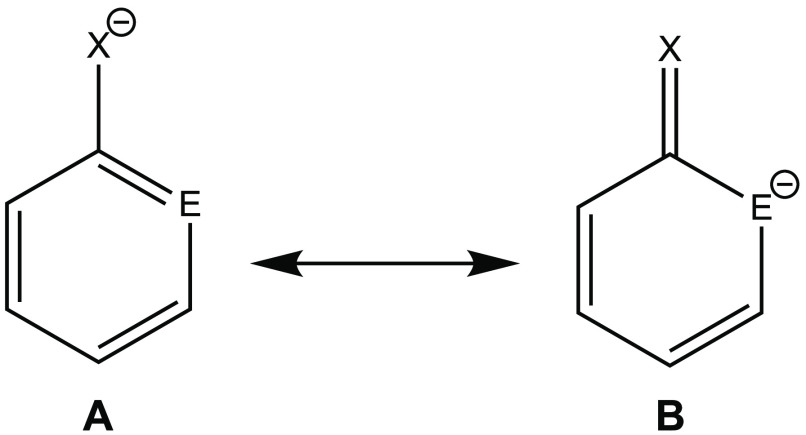
Representative examples for the aromatic (**A**) and nonaromatic (**B**) resonance structures of the phospha- and arsaphenolate anions (E = P, As; X = O, S, Se).

The lower aromaticity of the anionic pnictaphenolate derivatives is due to the substantial contribution of nonaromatic resonance structures (e.g., **B** in Figure 5), similar to the phenolate anion.^85^ The weights of the possible resonance structures were obtained by natural resonance theory (NRT) analyses (Tables 5 and S10).

In the order O > S > Se, the NICS(1) values of the pnictaphenolates decrease, along with increasing aromaticity, which is in line with the more exothermic reaction energies. This trend is also in nice accordance with our NRT calculations, which show an increasing contribution of the aromatic resonance structures, peaking in the case of the neutral phosphabenzene (Table 5 and Figure S4).

Because the nonaromatic resonance structures contain an exocyclic C=X π bond, the change in the aromaticity can be scrutinized by the relative strength of these π bonds. The bonding energies related to the *π contributions* of the C=O, C=S, and C=Se bonds are 88.0, 55.4, and 44.3 kcal·mol^–1^, respectively (Scheme S1 and Table S13).^86^ Correspondingly, the strongest π bond is in the C=O moiety, which therefore has the lowest ability to take part in cyclic delocalization. This results in a higher contribution of nonaromatic resonance structures (**B**), which obviously decreases the aromaticity. In line with the decrease in the π(C=X) bonding energy in the order of O > S > Se, the contribution of the nonaromatic structures decreases, and that of the aromatic structures increases.

#### TS2

Even though the rDA reactions are useful synthetic tools for the production of unsaturated compounds from bicyclic or cyclic compounds and are widely employed in the chemistry of 2-pyrones and cyclopentadienones,^87,88^ the rDA reactions are clearly less explored compared to the DA reactions.^89,90^ Utilizing the theory of microscopic reversibility,^91,92^ the rDA reactions are typically explained by the reverse route, that is, the reaction going backward from the products to the bicyclic intermediates. Thus, FMO theory can also be applied to understand the reactivity trends. On the basis of the diene character (corresponding to nonaromatic resonance structure **B**), we discuss the characterization of TS2 using the back-route analogy.

In these back-route reactions, the anionic pnictaphenolate species play the role of dienes and CO_2_ acts as a dienophile. All of the 2-pnictaphenolates have very similar and high HOMO energies of around −2 eV (Table 5), and the LUMOs are also destabilized (≈2.3 eV; Table S8). The HOMO of CO_2_ is rather stabilized with an energy of −12.6 eV, and its LUMO lies at −0.1 eV. Therefore, the stabilizing orbital interaction arises between the HOMO of the diene and the LUMO of CO_2_, so these reactions can be considered to be NED DA reactions.

Again, we obtained the same descriptors as those above for the DA step: NPA charges, nucleophilic and electrophilic Parr function values, the CT values, and the *A*_sy_ values of the TSs (Table S8). On the basis of the CT and *A*_sy_ values, the “anionic” and “neutral” routes again differ. With respect to the anions, the higher FMO gap, weaker nucleophilicity, and higher aromaticity of 2-methylphosphabenzene acting as a diene leads to lower *A*_sy_ [0.33 and 0.39 for RP(A) and RP(B), respectively] and moderate CT [RP(A), CT = 0.33 e; RP(B), CT = 0.39 e] values. In the cases of the anionic pnictaphenolates, both the *A*_sy_ and CT values can be found in a relatively narrow range, exceeding those of the neutral counterpart: the CT values are practically the same (0.46–0.51 e; Table S8) for all of the anions, and the *A*_sy_ values range between 0.53 and 0.63. This shows rather high ionic character as well as significant *A*_sy_. Thus, because of the electronic similarity of the “dienes” (pnictaphenolates), the structural and electronic characteristics in TS2 are also very similar.

## Conclusion

Herein, we have shown a computational study on the DA reactivity of the so-far-known P and As analogues of [ECX]^−^ anions to compare their cycloaddition activity and to clarify the fundamental aspects of the DA reactions involving anionic dienophiles.

In the cases of the [ECX]^−^ anions (E = P, As; X = O, S, Se) and a neutral model compound (MeC≡P), two possible RPs (A and B) were studied. Along RP(A), the pnictogen center E attacks the more electrophilic (and positively charged) center of 2-pyrone, while in RP(B), it attacks the less electrophilic (and negatively charged) center. The electronic properties (HOMO energy, global nucleophilicity, and Parr functions) of the anions acting as dienophiles markedly influence the activation barriers. For RP(A), a good correlation was found between the barriers of the DA step and the global and local nucleophilicities, and the trend is reversed for RP(B). These observations can be explained by the differing nucleophilicities and charge distributions of the anions. Clearly, the chalcogen centers (X) have an important tailoring effect by influencing the local nucleophilicity of the pnictogen center (E). The high nucleophilicity of the O-containing analogues ([ECO]^−^) causes a barrier-lowering effect in comparison with the heavier chalcogen-containing congeners.

Furthermore, the structural and electronic properties of the TS1s have been analyzed using *A*_sy_ and global/local nucleophilicity indices. On the basis of these, we can characterize this *anionic* type of DA reaction: the anionic dienophile exhibits strongly localized nucleophilicity and the ability to transfer a large amount of electron density toward the diene during the reaction, resulting in a highly asynchronic TS (*A*_sy_ > 0.5) combined with high CT values (>0.5 e).

Additionally, the reaction energies of these DA reactions show a correlation with the aromaticity in the anionic pnictaphenolate products: the lower the aromaticity is, the less exothermic the reaction sequence is. This effect is the most pronounced for the O-containing phospha- and arsaphenolates, which have the lowest aromatic character because of the high stability of the exocyclic C=O π bond.

Because all of the investigated reactions can be considered to be feasible, further new anionic and neutral 2-pnictaphenol analogues can be accessible in the future. Knowing the outstanding coordination properties of the phosphabenzenes and phosphaphenols, a similar potential may be expected for the heavier analogues as well, especially if the tailoring effects of the chalcogen and pnictogen atoms are considered.

## Computational Methods

In our study, we employed the *Gaussian09*(93) and *Mrcc*(94,95) suites of programs. All of the geometries were optimized at the B3LYP-D3/aug-cc-pVTZ level of theory, and single-point-energy calculations were performed at the M06-2X/aug-cc-pVTZ and DF-CCSD(T)/aug-cc-pVTZ levels of theory. The effect of solvation was simulated by applying PCM with THF. Harmonic vibrational analyses were carried out both in a vacuum and using PCM; for local minima, all force constants were positive, while for TSs, one imaginary frequency was found. Optimization of the TSs was carried out using the force constants from a previous vibrational analysis, and intrinsic reaction coordinate calculations were performed both forward and backward along the reaction coordinate.

The accuracy of the B3LYP-D3/aug-cc-pVTZ geometries was tested in a vacuum by geometry optimizations on a model reaction [RP(A) pathway of the [PCO]^−^ anion] at various levels (MP2/aug-cc-pVTZ, M06-2X/aug-cc-pVTZ, and ωB97X-D/aug-cc-pVTZ; Table S5). All of these were found to be very similar (no significant changes were observed), and the difference in electronic energies is also minor (less than 0.6 kcal·mol^–1^); therefore, the B3LYP-D3 geometries were used in further calculations. Then, coupled-cluster (CC) methods were tested in a vacuum, and calculations were carried out at the CCSD(T)/aug-cc-pVTZ, DF-CCSD(T)/aug-cc-pVTZ, and DF-CCSD(T)/aug-cc-pVQZ levels of theory (Table S6). All of these calculations resulted in similar relative energy profiles; thus, the (most cost-effective) DF-CCSD(T)/aug-cc-pVTZ method was chosen for comparison. The B3LYP-D3, M06-2X, and ωB97X-D functionals combined with the aug-cc-pVTZ basis set were tested (in a vacuum using single-point calculations) to find an appropriate functional for PCM calculations (note that the solvent effects cannot be simulated with the CC methods). The M06-2X/aug-cc-pVTZ level fits the best to the CC results (the average difference between the relative energy of the stationary points:  = 3.1 kcal·mol^–1^), which is in accordance with a previous study on the cycloadditions of [PCO]^−^.^26^ Thus, for the calculations both in a vacuum and using PCM, the M06-2X functional was employed. For detailed results, see the Supporting Information.

The NPA charges and Wiberg bond indices (WBIs) were calculated at the M06-2X/aug-cc-pVTZ and B3LYP-D3/aug-cc-pVTZ (Tables 3, S2, and S3) levels of theory using the *NBO 3.1* program.^96^ The NRT analyses were performed at the M06-2X/aug-cc-pVTZ level using the *NBO 5.9* package (Table S10).^97^ The nucleophilic (*P*_k_^–^) and electrophilic (*P*_k_^+^) Parr functions were determined at the same levels as the Mulliken atomic spin density of the corresponding radicals obtained by the removal or addition of an electron, respectively. The HOMO energies were obtained at the M06-2X/aug-cc-pVTZ level of theory (Tables 3 and S2), and the NICS calculation were carried out at the B3LYP/aug-cc-pVTZ level of theory (Tables 5 and S9).

Besides the Parr functions, *N*(71) was calculated for the dienophiles as *N* = ε_HOMO,dienophile_ – ε_HOMO,TCE_ (eV), where TCE stands for tetracyanoethylene, [C_2_(CN)_4_], having a HOMO energy of −11.04 eV at the M06-2X/aug-cc-pVTZ level of theory.

We introduced *A*_sy_ based on transient bond valences (*v*) as *A*_sy_ = |*v*_2_ – *v*_1_|/(*v*_2_ + *v*_1_). The value of the *A*_sy_ index is 1 and 0 for fully asynchronous and synchronous reactions, respectively. The bond valences *v*_1_ and *v*_2_ denote the C–C and C–E distances, respectively; calculated as *v* = exp[−(*d*_TS_ – *d*_INT_)/*A*], where *A* is an empirically defined factor (0.37 Å)^98,99^ and *d*_TS_ and *d*_INT_ refer to the appropriate atomic distances in the TSs and in the intermediates, respectively (for raw data, see Tables S3 and S11). To check the accuracy of our method, we calculated *A*_sy_′ using the WBIs as *A*_sy_′ = [WBI(E,TS1)/WBI(E,INT) – WBI(C,TS1)/WBI(C,INT)]/ [WBI(E,TS1)/WBI(E,INT) + WBI(C,TS1)/WBI(C,INT)] (for raw data, see Table S3).^100^ The two methods delivered similar trends for *A*_sy_; however, those based on transient bond valences can be obtained in a simpler way.

## Supporting Information Available

Gibbs free energy profiles for the reactions, method testing, raw data for calculation of the descriptors (CT and *A*_sy_), and optimized geometries with total energies (PDF)

## Dedication

The authors dedicate this paper to Prof. László Nyulászi on the occasion of his 65th birthday.
