# Supplementary material for: Understanding the Mechanism of Diels–Alder Reactions with Anionic Dienophiles: A Systematic Comparison of [ECX]− (E = P, As; X = O, S, Se) Anions
Source: Inorg Chem. 2022 May 9;61(20):7922–34. doi: 10.1021/acs.inorgchem.2c00549 (PMC9131451; doi:10.1021/acs.inorgchem.2c00549)
Supplement: Supplementary file 1 — ic2c00549_si_001.pdf [file ic2c00549_si_001.pdf]

Supporting Information

Understanding the mechanism of Diels–Alder reactions with anionic dienophiles: a systematic comparison of $[ECX]^-$ (E: P, As; X: O, S, Se) anions

*Ádám Horváth, Zoltán Benkő**

Department of Inorganic and Analytical Chemistry, Budapest University of Technology and Economics, Műegyetem rkp. 3., H-1111 Budapest, Hungary, E-mail: benko.zoltan@vbk.bme.hu

Table of contents

GIBBS FREE ENERGY PROFILES: ... 3

PROPERTIES OF THE DIENOPHILES AND INDICES FOR TS1: ... 4

METHOD TESTING: ... 7

CALCULATED PROPERTIES OF THE PNICTAPHENOLATES: ... 15

CALCULATION OF THE BONDING ENERGY OF THE C=X BONDS: ... 19

CALCULATED GEOMETRIES AND THEIR ENERGIES: ... 20

GAS PHASE GEOMETRIES AND THEIR EXACT ENERGIES: ... 20
2*H*-PYRAN-2-ONE: ... 20
CO_2: ... 20
$[PCO]^-$: ... 21
$[PCS]^-$: ... 26
$[PCSe]^-$: ... 32
$[AsCO]^-$: ... 37
$[AsCS]^-$: ... 43
$[AsCSe]^-$: ... 48
MECP: ... 54
PCM GEOMETRIES AND THEIR EXACT ENERGIES: ... 60
2*H*-PYRAN-2-ONE: ... 60
CO_2: ... 60
$[PCO]^-$: ... 60
$[PCS]^-$: ... 66
$[PCSe]^-$: ... 72
$[AsCO]^-$: ... 77
$[AsCS]^-$: ... 83
$[AsCSe]^-$: ... 88
MECP: ... 94

Gibbs free energy profiles:

Table S1 Calculated relative Gibbs free energy profiles for both steps along RP(A) and RP(B). **TS1** and **TS2** represent the transition states for the first ([4+2] cycloaddition) and the second step (elimination of CO_2), respectively. **RC** and **PC** represent the reactant and product complexes, respectively. The values with normal letters and **in bold** present the calculations in vacuum and applying PCM=THF at M06-2X/aug-cc-pVTZ level, respectively. All values are given in kcal·mol^{-1} and are compared to the Gibbs free energy of the separated reactants.

Dienophile (pathway)	RC	TS1	INT	TS2	PC	PRO
$[PCO]^{-}$ (A)	-5.1 **5.5**	16.7 **27.5**	2.9 **12.2**	17.7 **26.5**	-29.3 **-18.1**	-30.5 **-23.9**
$[PCO]^{-}$ (B)	-5.7 **5.0**	25.1 **37.0**	0.0 **9.0**	28.7 **36.8**	-27.1 **-17.4**	
$[PCS]^{-}$ (A)	-5.7 **4.1**	24.0 **32.6**	-0.8 **6.4**	17.4 **24.5**	-37.1 **-28.7**	-39.4 **-35.5**
$[PCS]^{-}$ (B)	-4.9 **5.0**	25.4 **34.3**	-5.7 **2.0**	19.2 **25.8**	-35.4 **-27.3**	
$[PCSe]^{-}$ (A)	-4.5 **4.9**	25.7 **33.5**	-2.5 **4.1**	17.3 **23.7**	-40.2 **-33.0**	-42.6 **-39.3**
$[PCSe]^{-}$ (B)	-4.5 **5.0**	24.5 **32.6**	-7.6 **-0.3**	16.1 **22.3**	-40.2 **-33.0**	
$[AsCO]^{-}$ (A)	-4.4 **5.7**	16.7 **26.8**	2.3 **11.4**	20.8 **28.9**	-23.8 **-14.9**	-27.5 **-20.9**
$[AsCO]^{-}$ (B)	-4.4 **4.9**	22.9 **34.0**	-2.2 **6.7**	29.2 **36.9**	-24.2 **-14.3**	
$[AsCS]^{-}$ (A)	-5.8 **3.3**	21.3 **29.3**	-3.9 **3.4**	17.3 **23.8**	-36.9 **-29.5**	-39.6 **-35.5**
$[AsCS]^{-}$ (B)	-6.0 **3.72**	21.1 **29.8**	-10.1 **-2.5**	16.7 **23.0**	-35.5 **-29.0**	
$[AsCSe]^{-}$ (A)	-4.2 **5.4**	23.9 **30.9**	-4.5 **2.4**	17.9 **23.9**	-39.2 **-32.5**	-42.0 **-38.4**
$[AsCSe]^{-}$ (B)	-4.2 **4.9**	21.5 **29.5**	-11.0 **-3.5**	14.7 **20.5**	-37.6 **-32.1**	
Me-CP (A)	-4.8 **6.2**	30.2 **31.1**	-3.0 **-2.0**	19.5 **20.5**	-49.5 **45.6**	-55.6 **-50.9**
Me-CP (B)	-4.8 **6.2**	29.5 **29.9**	-5.8 **-5.3**	16.3 **16.1**	-49.5 **45.4**	

Properties of the dienophiles and indices for TS1:

Table S2 NPA partial charges (q, in electrons), nucleophilic Parr function (P_k^-) and HOMO energies (ε_{HOMO}, in eV) calculated at the B3LYP-D3/aug-cc-pVTZ level of theory. From the HOMO energies the N nucleophilicity indices were calculated.

	q			P_k^-		
$[ECX]^-$	E	C	X	E	C	X
$[PCO]^-$	-0.43	0.09	-0.66	0.72	0.06	0.22
$[PCS]^-$	-0.04	-0.72	-0.24	0.55	-0.10	0.55
$[PCSe]^-$	0.02	-0.80	-0.21	0.46	-0.10	0.64
$[AsCO]^-$	-0.45	0.10	-0.65	0.75	0.09	0.17
$[AsCS]^-$	-0.05	-0.74	-0.22	0.61	-0.06	0.45
$[AsCSe]^-$	0.02	-0.84	-0.18	0.53	-0.09	0.56
P≡C-Me	-0.33	0.28	-	0.46	0.48	-

Table S3 The bridging C-C and C-E atomic distances [d(C-C), d(C-E)], the calculated bond valences from these distances [v_1, v_2; $v_{1,2}$=exp{-(d_{TS}-d_{INT})/A}] and the Wiberg bond indices of these bonds (WBI(C-C), WBI(C-E)) in the TS1 and INT structures at M06-2X/aug-cc-pVTZ (in brackets) and B3LYP-D3/aug-cc-pVTZ levels of theory.

Reaction	d(C-C)		WBI(C-C)		v_1	d(C-E)		WBI(C-E)		v_2
	TS1	INT	TS1	INT	-	TS1	INT	TS1	INT	-
$[PCO]^-$ (A)	2.48	1.61	0.25 (0.24)	0.87 (0.88)	0.10	2.00	1.91	0.81 (0.82)	0.89 (0.89)	0.79
$[PCO]^-$ (B)	2.41	1.58	0.28 (0.27)	0.90 (0.90)	0.11	2.22	1.97	0.59 (0.59)	0.78 (0.78)	0.51
$[PCS]^-$ (A)	2.52	1.56	0.26 (0.27)	0.94 (0.94)	0.08	2.05	1.92	0.73 (0.73)	0.86 (0.86)	0.71
$[PCS]^-$ (B)	2.29	1.55	0.36 (0.36)	0.96 (0.96)	0.13	2.37	1.97	0.46 (0.44)	0.77 (0.77)	0.34
$[PCSe]^-$ (A)	2.55	1.56	0.26 (0.26)	0.96 (0.96)	0.07	2.10	1.93	0.68 (0.68)	0.85 (0.85)	0.63
$[PCSe]^-$ (B)	2.21	1.54	0.40 (0.39)	0.98 (0.97)	0.16	2.50	1.97	0.39 (0.37)	0.77 (0.77)	0.24
$[AsCO]^-$ (A)	2.49	1.61	0.25 (0.24)	0.88 (0.88)	0.09	2.17	2.05	0.75 (0.76)	0.84 (0.84)	0.74
$[AsCO]^-$ (B)	2.33	1.58	0.32 (0.32)	0.91 (0.91)	0.13	2.44	2.11	0.50 (0.50)	0.73 (0.72)	0.41
$[AsCS]^-$ (A)	2.49	1.56	0.28 (0.29)	0.95 (0.95)	0.08	2.22	2.06	0.67 (0.67)	0.81 (0.81)	0.65
$[AsCS]^-$ (B)	2.25	1.55	0.38 (0.38)	0.97 (0.97)	0.15	2.57	2.11	0.40 (0.39)	0.72 (0.72)	0.29
$[AsCSe]^-$ (A)	2.49	1.56	0.28 (0.29)	0.96 (0.96)	0.08	2.28	2.07	0.62 (0.61)	0.81 (0.80)	0.57
$[AsCSe]^-$ (B)	2.22	1.54	0.40 (0.39)	0.98 (0.98)	0.16	2.65	2.11	0.35 (0.34)	0.72 (0.72)	0.23
Me-CP (A)	2.20	1.53	0.39 (0.38)	0.98 (0.98)	0.16	2.47	1.93	0.41 (0.40)	0.86 (0.86)	0.17
Me-CP (B)	2.16	1.53	0.41 (0.39)	0.99 (0.98)	0.18	2.59	1.93	0.33 (0.32)	0.83 (0.82)	0.17

Table S4 The asynchronicity indexes (A_{sy}), charge transfer (CT) values and the local charge transfer (Δq) for TS1. The As_{y} values were calculated with bond valance (BV) method and using Wiberg Bond Indexes (WBI) of the corresponding (TS1 and INT) structures at M06-2X/aug-cc-pVTZ (in brackets) and B3LYP-D3/aug-cc-pVTZ levels of theory. The local and global charge transfer values (CT and Δq, respectively in e) were calculated at B3LYP-D3/aug-cc-pVTZ level of theory.

Reaction	CT (e)	Δq			A_{sy}^{BV} (-)	A_{sy}^{WBI}(-)
		E	C	X		
$[PCO]^{-}$ (A)	0.795	0.55	0.14	0.10	0.78	0.52 (0.54)
$[PCO]^{-}$ (B)	0.594	0.48	0.05	0.06	0.66	0.42 (0.42)
$[PCS]^{-}$ (A)	0.625	0.39	0.05	0.18	0.81	0.51 (0.50)
$[PCS]^{-}$ (B)2	0.362	0.35	-0.03	0.04	0.43	0.23 (0.21)
$[PCSe]^{-}$ (A)	0.555	0.36	0.02	0.16	0.80	0.50 (0.49)
$[PCSe]^{-}$ (B)	0.287	0.33	-0.05	-0.01	0.19	0.10 (0.09)
$[AsCO]^{-}$ (A)	0.765	0.53	0.15	0.09	0.78	0.52 (0.53)
$[AsCO]^{-}$ (B)	0.547	0.46	0.05	0.04	0.50	0.32 (0.33)
$[AsCS]^{-}$ (A)	0.586	0.38	0.05	0.16	0.78	0.47 (0.47)
$[AsCS]^{-}$ (B)	0.354	0.35	-0.02	0.04	0.33	0.17 (0.16)
$[AsCSe]^{-}$ (A)	0.508	0.35	0.03	0.13	0.75	0.45 (0.44)
$[AsCSe]^{-}$ (B)	0.297	0.34	-0.03	-0.01	0.19	0.09 (0.08)
Me-CP (A)	0.06	0.06	0.02	0.02	0.00	0.10 (0.09)
Me-CP (B)	0.07	0.10	-0.03	0.03	-0.04	0.01 (0.02)

Method Testing:

Table S5 Reaction profiles using all investigated DFT levels of theory and the DF-CCSD(T) method in combination with the aug-cc-pVTZ basis set.

Dienophile (pathway)	Method	RC	TS1	INT	TS2	PC	PRO
$[PCO]^{-}$ (A)	B3LYP-D3	-14.4	8.8	-1.6	7.9	-37.4	-29.6
	M06-2X	-14.4	5.4	-10.6	5.9	-38.1	-29.8
	ωB97XD	-13.8	9.1	-8.4	6.2	-37.5	-30.6
	DF-CCSD(T)	-14.9	4.1	-14.0	-0.4	-39.4	-31.4
$[PCO]^{-}$ (B)	B3LYP-D3	-15.1	16.7	-4.2	17.0	-34.5	-29.6
	M06-2X	-15.0	14.3	-13.7	17.4	-35.3	-29.8
	ωB97XD	-14.5	19.0	-10.9	17.7	-35.2	-30.6
	DF-CCSD(T)	-15.8	12.7	-16.7	11.4	-36.8	-31.4
$[PCS]^{-}$ (A)	B3LYP-D3	-14.5	14.3	-5.0	7.3	-43.0	-37.4
	M06-2X	-14.6	12.6	-14.8	5.6	-45.3	-39.2
	ωB97XD	-14.0	16.5	-13.2	5.0	-45.9	-41.0
	DF-CCSD(T)	-15.4	10.0	-18.7	-0.9	-46.5	-40.4
$[PCS]^{-}$ (B)	B3LYP-D3	-13.6	17.2	-9.7	8.1	-41.8	-37.4
	M06-2X	-14.1	14.2	-19.8	7.8	-43.8	-39.2
	ωB97XD	-13.0	19.3	-17.8	7.3	-44.8	-41.0
	DF-CCSD(T)	-13.9	12.0	-23.3	1.9	45.2	-40.4

Dienophile (pathway)	Method	RC	TS1	INT	TS2	PC	PRO
$[PCSe]^{-}$ (A)	B3LYP-D3	-13.5	15.3	-7.2	6.0	-46.5	-41.0
	M06-2X	-12.9	15.5	-15.6	6.2	-47.6	-41.7
	ωB97XD	-12.8	17.9	-15.2	4.1	-49.3	-44.5
	DF-CCSD(T)	-13.5	11.4	-20.6	-1.5	-49.6	-43.7
$[PCSe]^{-}$ (B)	B3LYP-D3	-13.5	16.1	-12.2	4.4	-46.5	-41.0
	M06-2X	-12.9	13.9	-20.9	5.4	-47.6	-41.7
	ωB97XD	-12.8	17.9	-20.1	3.8	-49.3	-44.5
	DF-CCSD(T)	-13.5	10.7	-25.6	-1.3	-49.6	-43.7
$[AsCO]^{-}$ (A)	B3LYP-D3	-13.8	8.2	-2.5	10.0	-31.8	-27.3
	M06-2X	-13.7	5.6	-11.1	9.2	-31.3	-26.7
	ωB97XD	-13.3	9.1	-8.8	8.7	-32.5	-28.4
	DF-CCSD(T)	-14.3	4.1	-14.3	2.4	-34.1	-29.1
$[AsCO]^{-}$ (B)	B3LYP-D3	-14.5	13.7	-6.8	16.4	-32.3	-27.3
	M06-2X	-14.8	12.0	-15.8	18.2	-32.2	-26.7
	ωB97XD	-13.9	16.8	-13.0	17.8	-33.0	-28.4
	DF-CCSD(T)	-14.9	10.1	-18.5	11.5	-34.5	-29.1

Dienophile (pathway)	Method	RC	TS1	INT	TS2	PC	PRO
$[AsCS]^-$ (A)	B3LYP-D3	-13.5	13.0	-6.5	8.5	-41.7	-36.2
	M06-2X	-13.9	11.4	-16.6	6.9	-43.9	-37.9
	ωB97XD	-13.0	15.5	-14.6	6.2	-45.0	-40.1
	DF-CCSD(T)	-13.6	9.0	-19.5	0.9	-45.2	-39.2
$[AsCS]^-$ (B)	B3LYP-D3	-14.2	13.7	-12.7	6.5	-40.7	-36.2
	M06-2X	-14.2	11.1	-22.9	6.7	-42.6	-37.9
	ωB97XD	-13.7	16.1	-20.5	5.9	-44.0	-40.1
	DF-CCSD(T)	-14.9	9.2	-25.6	1.0	-44.0	-39.2
$[AsCSe]^-$ (A)	B3LYP-D3	-13.5	13.6	-8.9	7.1	-45.4	-39.9
	M06-2X	-13.6	12.6	-18.6	6.2	-47.7	-41.8
	ωB97XD	-12.9	16.5	-16.7	5.2	-48.6	-43.8
	DF-CCSD(T)	-13.3	10.8	-21.5	0.2	-48.6	-42.7
$[AsCSe]^-$ (B)	B3LYP-D3	-13.5	12.4	-15.3	2.6	-44.2	-39.9
	M06-2X	-13.6	10.1	-25.1	3.1	-46.3	-41.8
	ωB97XD	-12.9	14.7	-22.9	2.3	-47.5	-43.8
	DF-CCSD(T)	-13.3	8.0	-27.9	-2.5	-47.3	-42.7

Dienophile (pathway)	Method	RC	TS1	INT	TS2	PC	PRO
Me-CP (A)	B3LYP-D3	-5.2	19.5	-10.1	7.1	-59.8	-56.6
	M06-2X	-5.1	17.0	-18.6	6.2	-59.9	-56.7
	ωB97XD	-5.0	20.6	-18.6	4.7	-62.5	-59.8
	DF-CCSD(T)	-5.7	15.1	-21.8	2.4	-55.8	-57.0
Me-CP (B)	B3LYP-D3	-5.2	19.0	-12.6	4.1	-59.8	-59.8
	M06-2X	-5.1	16.8	-21.4	2.9	-59.9	-56.7
	ωB97XD	-5.0	20.4	-21.1	1.7	-62.5	-59.8
	DF-CCSD(T)	-5.7	15.1	-24.5	-0.9	-55.8	-57.0

Table S6 The calculations on $[PCO]^-$ (A) during the method testing, the geometry was optimized at the B3LYP-D3/aug-cc-pVTZ level of theory.

Method/basis -type(opt/SP)	A	TS1	INT	TS2	PRO
B3LYP/6-31+G* -opt	0.0	12.1	-1.5	8.8	-27.3
B3LYP-D3/aug-cc-pVTZ -opt	0.0	8.8	-1.6	7.9	-29.6
M06-2X/aug-cc-pVTZ -SP	0.0	5.4	-10.6	5.9	-29.8
M06-2X/aug-cc-pVTZ -opt	0.0	5.7	-10.7	6.2	-29.7
ωB97X-D/aug-cc-pVTZ -SP	0.0	9.1	-8.4	6.2	-30.6
ωB97X-D/aug-cc-pVTZ -opt	0.0	9.1	-8.4	6.4	-30.5
MP2/aug-cc-pVTZ -SP	0.0	0.4	-12.6	1.7	-35.3
MP2/aug-cc-pVQZ -SP	0.0	1.2	-11.8	2.4	-35.9
MP2/aug-cc-pVTZ -opt	0.0	0.8	-13.0	1.1	-35.8
DF-CCSD(T)/aug-cc-pVTZ	0.0	4.1	-14.0	-0.4	-31.4
DF-CCSD(T)/aug-cc-pVQZ	0.0	5.0	-13.2	0.5	-32.1
CCSD(T)/aug-cc-pVTZ (Gaussian09)	0.0	4.0	-14.2	-0.5	-31.5
LNO-CCSD(T)/aug-cc-pVTZ -SP	0.0	4.1	-14.0	-0.4	-31.4
LNO-CCSD(T)/aug-cc-pVQZ -SP	0.0	5.2	-13.0	1.0	-32.1

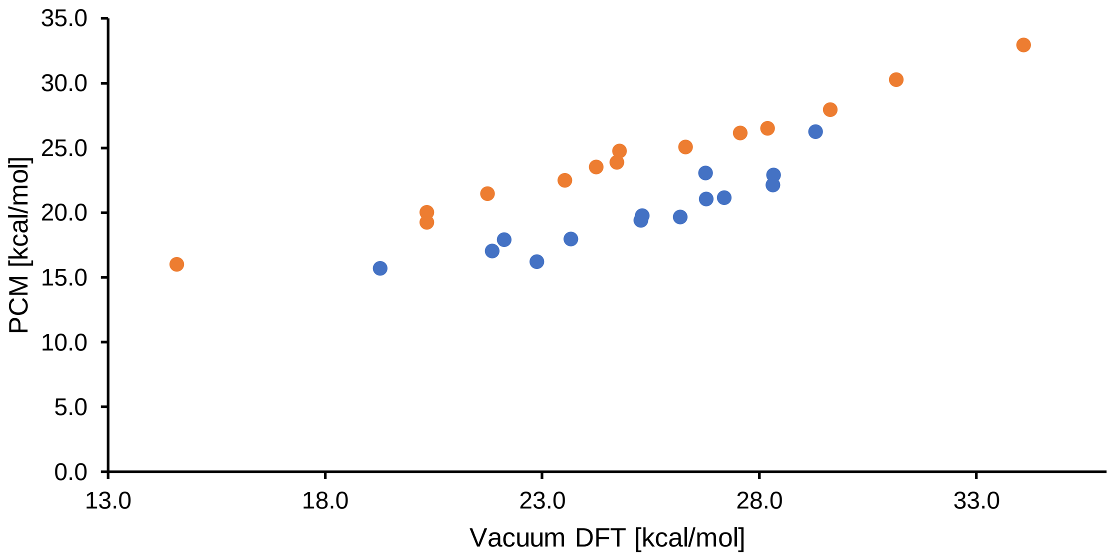

Figure S1 Plot of $\Delta E_1^{\ddagger}$ (blue) and $\Delta E_2^{\ddagger}$ (orange) barriers: M06-2X/aug-cc-pVTZ(PCM) versus M06-2X/aug-cc-pVTZ (vacuum) levels of theory.

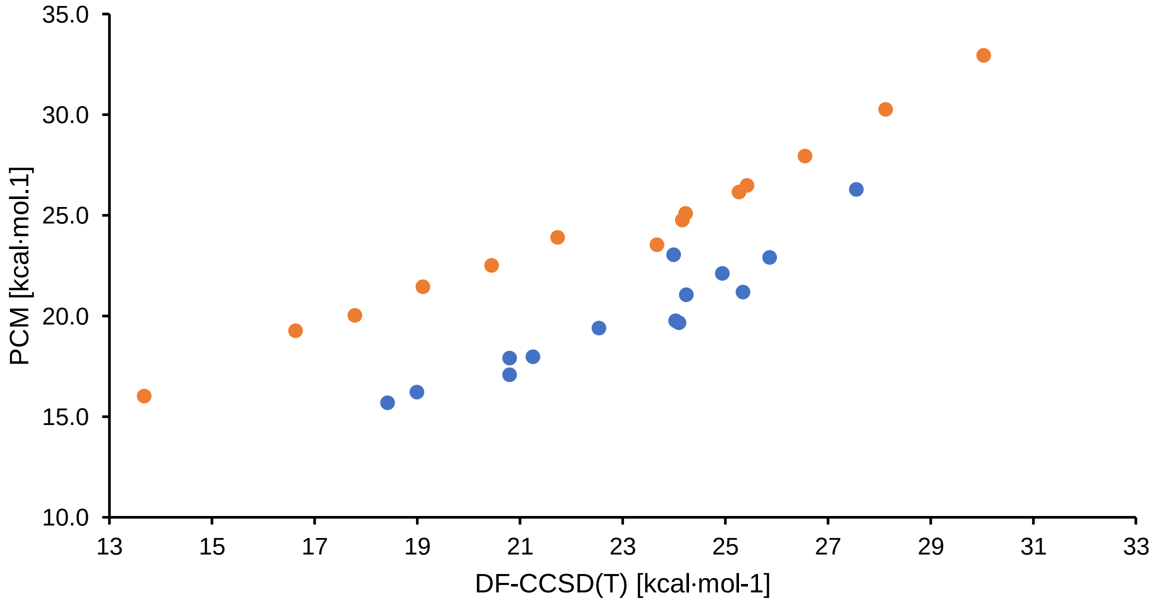

Figure S2 Plot of $\Delta E_1^{\ddagger}$ (blue) and $\Delta E_2^{\ddagger}$ (orange) barriers: M06-2X/aug-cc-pVTZ (PCM) versus DF-CCSD(T)/aug-cc-pVTZ levels of theory.

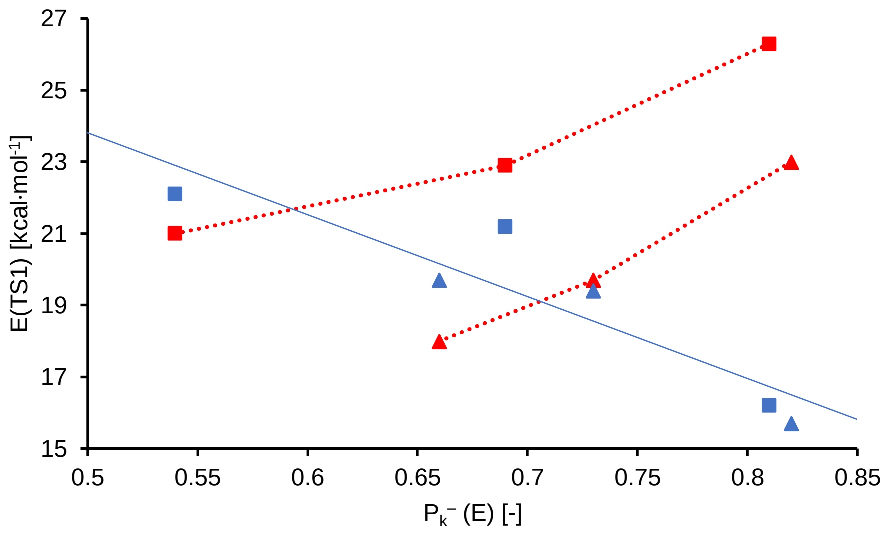

Figure S3 Plot of the P_k^- of the pnictogen versus the first activation barrier.

Table S7 Calculated relative energies along the two reaction pathways. The **TS1** and **TS2** represents the transition states for the [4+2] cycloaddition, elimination of CO_2, respectively. **RC** and **PC** represent the reactant and the product complexes, respectively. The values **in bold**, parenthesises and *italics* present the DF-CCSD(T)/aug-cc-pVTZ, the gas phase M06-2X/aug-cc-pVTZ and the PCM calculations (with THF) at M06-2X/aug-cc-pVTZ level. All values are given in kcal·mol^{-1} and are compared to the energy of the separated reactants (dienophile + 2*H*-pyrone).

[ECX]$^-$ anion	RC	TS1	INT	TS2	PC	PRO
[PCO]$^-$ (A)	**-14.9** (-14.4) *-3.4*	**4.1** (5.4) *16.2*	**-14.0** (-10.6) *-1.5*	**-0.4** (5.9) *14.5*	**-39.4** (-38.1) *-26.9*	**-31.4** (-29.8) *-23.4*
**[PCO]$^-$ (B)**	**-15.8** (-15.0) *-3.7*	**12.7** (14.3) *26.3*	**-16.7** (-13.7) *-4.8*	**11.4** (17.4) *25.4*	**-36.8** (-35.3) *-25.1*	
[PCS]$^-$ (A)	**-15.3** (-14.6) *-3.9*	**10.0** (12.6) *21.2*	**-18.7** (-14.8) *-7.6*	**-0.9** (5.6) *12.4*	**-46.5** (-45.3) *-38.6*	**-40.4** (-39.2) *-35.5*
[PCS]$^-$ (B)	**-13.9** (-14.1) *-4.2*	**12.0** (14.2) *22.9*	**-23.4** (-19.8) *-12.1*	**1.9** (7.8) *14.1*	**-45.2** (-43.8) *-36.1*	
[PCSe]$^-$ (A)	**-13.5** (-12.9) *-4.1*	**11.4** (15.5) *22.1*	**-20.6** (-15.6) *-10.0*	**-1.5** (6.2) *11.4*	**-49.6** (-47.6) *-42.6*	**-43.7** (-41.7) *-39.6*
[PCSe]$^-$ (B)	**-13.5** (-12.9) *-4.1*	**10.8** (13.9) *21.0*	**-25.6** (-20.9) *-14.6*	**-1.3** (5.4) *10.4*	**-49.6** (-47.6) -42.6	
**[AsCO]$^-$ (A)**	**-14.3** (-13.7) *-3.3*	**4.1** (5.6) *15.7*	**-14.3** (-11.1) *-2.1*	**2.4** (9.2) *17.1*	**-34.1** (-31.3) *-23.5*	**-29.1** (-26.7) *-20.3*
[AsCO]$^-$ (B)	**-14.9** (-14.8) *-4.1*	**10.1** (12.0) 23.0	**-18.5** (-15.8) -7.1	**11.5** (18.2) *25.8*	**-34.5** (-32.2) *-23.1*	
[AsCS]$^-$ (A)	**-13.6** (-13.9) *-4.3*	**9.0** (11.4) *19.4*	**-19.5** (-16.6) *-9.3*	**1.0** (6.9) *13.3*	**-45.2** (-43.9) *-37.1*	**-39.2** (-37.9) *-34.0*
[AsCS]$^-$ (B)	**-14.9** (-14.2) *-4.1*	**9.2** (11.1) *19.7*	**-25.6** (-22.9) *-15.1*	**-1.0** (6.7) *12.8*	**-44.0** (-42.6) *-36.5*	
[AsCSe]$^-$ (A)	**-13.3** (-13.6) *-3.1*	**10.8** (12.6) *19.7*	**-21.6** (-18.6) *-11.9*	**0.2** (6.2) *12.0*	**-48.6** (-47.7) *-41.7*	**-42.7** (-41.8) *-38.6*
[AsCSe]$^-$ (B)	**-13.3** (-13.6) *-4.3*	**8.0** (10.1) *18.0*	**-28.0** (-25.1) *-17.8*	**-2.5** (3.1) *8.7*	**-47.3** (-46.3) *-41.0*	
Me-CP (A)	**-5.7** (-5.1) *-3.6*	**15.1** (17.0) *17.9*	**-21.8** (-18.6) *-17.6*	**2.4** (6.2) *7.2*	**-55.8** (59.9) *-55.3*	**-57.0** (-56.7) *-52.7*
**Me-CP (B)**	**-5.7** (-5.1) *-3.6*	**15.1** (16.8) *17.1*	**-24.5** (-21.4) *-20.8*	**-0.9** (2.9) *2.7*	**-55.8** (-59.9) *-55.7*	

Calculated properties of the pnictaphenolates:

Table S8 The properties of the pnictaphenolates: NPA charges (q; e), nucleophilic Parr function values (P_k^-; -) on the C3 (next to the oxido group) and C6 (next to the pnictogen) carbon atoms, the charge transfer (CT; e) in the corresponding TS2 (between the pnictaphenolate and CO_2) at the M06-2X/aug-cc-pVTZ and the energy of the HOMO (ε_{HOMO}; eV) at M06-2X/aug-cc-pVTZ level of theory.

Reaction	$\Delta E^{\ddagger}$	q		CT	ε_{HOMO}	P_k^-		A_{sy} (BV)
		C3	C6			C3	C6	
$[PCO]^-$ (A)	31.0	-0.34	-0.56	-0.51	-1.9	0.33	-0.24	0.53
$[PCO]^-$ (B)	42.8			-0.48				0.56
$[PCS]^-$ (A)	39.5	-0.27	-0.59	-0.48	-1.8	0.28	-0.15	0.54
$[PCS]^-$ (B)	42.3			-0.49				0.59
$[PCSe]^-$ (A)	42.2	-0.27	-0.59	-0.47	-1.8	0.21	0	0.54
$[PCSe]^-$ (B)	42.4			-0.49				0.60
$[AsCO]^-$ (A)	31.5	-0.35	-0.55	-0.52	-1.8	0.24	-0.32	0.58
$[AsCO]^-$ (B)	40.7			-0.50				0.60
$[AsCS]^-$ (A)	40.2	-0.28	-0.58	-0.49	-1.9	0.32	-0.27	0.58
$[AsCS]^-$ (B)	38.2			-0.51				0.63
$[AsCSe]^-$ (A)	42.9	-0.28	-0.58	-0.47	-1.8	0.21	0	0.57
$[AsCSe]^-$ (B)	40.2			-0.52				0.63
Me-CP (A)	-59.4	-0.22	-0.56	-0.29	-8.0	0.09	0.01	0.33
Me-CP (B)	-56.2			-0.34				0.39

Table S9 The NICS(0) values of the anionic pnictaphenolates with the appropriate E=P, As and X=O, S, Se heteroatoms at the B3LYP/aug-cc-pVTZ level of theory.

E	X	NICS(0)
P	O	-2.0
P	S	-1.7
P	Se	-1.8
As	O	-1.9
As	S	-1.7
As	Se	-1.8
P≡C-Me		-6.6

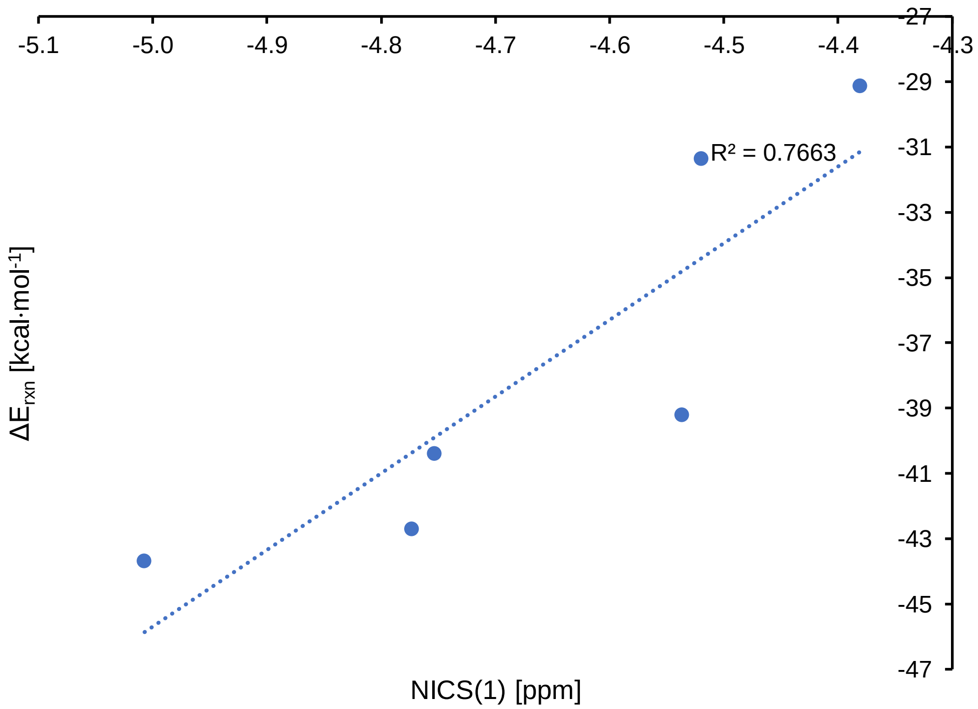

Figure S4 Plot of NICS(1) versus the relative stability of the anionic pnictaphenolates (ΔE_{rxn})

Table S10 The aromatic and non-aromatic resonance structures of the pnictaphenolates with contributions larger than 5% and the sum of the contribution of the aromatic and non-aromatic structures.

-	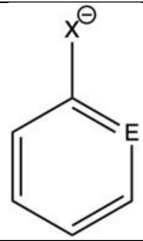		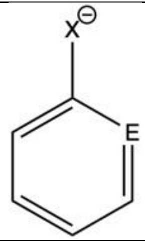		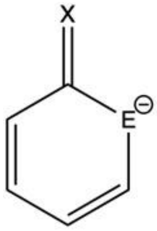		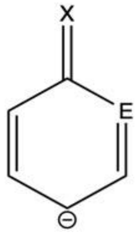		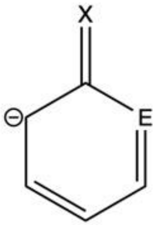		Aromatic		Non-aromatic	
**-**	**P**	**As**	**P**	**As**	**P**	**As**	**P**	**As**	**P**	**As**	**P**	**As**	**P**	**As**
O	16.03	11.08	9.95	4.25	16.69	18.37	12.80	7.91	9.95	10.58	25.98	15.33	39.44	36.86
S	15.43	26.39	15.51	14.73	6.81	9.17	-	-	5.26	5.95	30.94	41.09	12.07	15.12
Se	27.54	24.21	20.38	16.50	6.23	6.92	-	-	6.33	6.41	47.92	41.71	12.56	13.33

Table S11 The C-O and C-C atomic distances (d(C-C), d(C-O)) and the calculated BV bond valences v_{C-C} and v_{C-O} bond valances calculated from the intermediate and the second transition states involving the CO_2 moiety.

Reaction	d(C-C)		v_{C-C}	d(C-O)		v_{C-O}
	TS2	INT	-	TS2	INT	-
$[PCO]^-$ (A)	1.60	1.52	0.81	2.00	1.49	0.25
$[PCO]^-$ (B)	1.68	1.50	0.62	2.11	1.46	0.18
$[PCS]^-$ (A)	1.63	1.53	0.76	2.03	1.48	0.22
$[PCS]^-$ (B)	1.67	1.51	0.65	2.14	1.47	0.17
$[PCSe]^-$ (A)	1.64	1.53	0.74	2.04	1.48	0.22
$[PCSe]^-$ (B)	1.66	1.51	0.66	2.14	1.48	0.17
$[AsCO]^-$ (A)	1.61	1.52	0.79	2.05	1.48	0.21
$[AsCO]^-$ (B)	1.67	1.50	0.62	2.14	1.46	0.16
$[AsCS]^-$ (A)	1.64	1.53	0.73	2.07	1.47	0.20
$[AsCS]^-$ (B)	1.66	1.50	0.65	2.17	1.47	0.15
$[AsCSe]^-$ (A)	1.66	1.53	0.71	2.07	1.47	0.20
$[AsCSe]^-$ (B)	1.65	1.50	0.66	2.17	1.47	0.15
Me-CP (A)	1.78	1.54	0.52	1.96	1.46	0.26
Me-CP (B)	1.74	1.53	0.56	1.98	1.47	0.25

Calculation of the bonding energy of the C=X bonds:

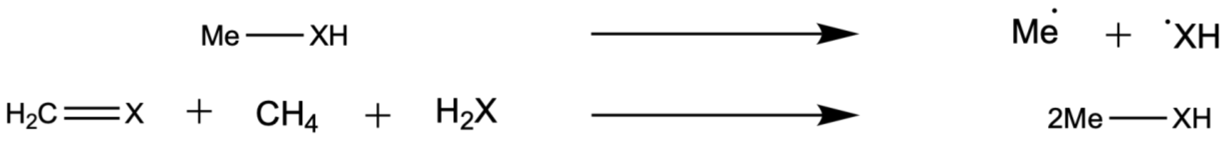

Scheme S1 The used reactions for approximating the bonding energy of the C=X π-bond, where X=O, S, Se.

The equation for the determination of the π bonding energies: $\mathbf{E_{\pi} = \Delta E_r(1) + \Delta E_r(2)}$

Table S12 The energies (E in a.u.) of the species from Scheme S1 were calculated at the MP4/6-31G*//HF/6-31G* level of theory and the zero point corrections (ZPE in a.u.) were calculated at the HF/6-31G* level of theory.

**Species**	**E**	**ZPE**
$CH_3\cdot$	-39.689181	0.030968
CH_4	-40.354552	0.04777
$HO\cdot$	-75.5356066	0.009105
CH_2=O	-114.1890706	0.029195
CH_3-OH	-115.3723416	0.055334
H_2O	-76.2063126	0.022981
$HS\cdot$	-398.1829875	0.006604
CH_2=S	-436.7891958	0.026851
CH_3-SH	-437.9909845	0.04963
H_2S	-398.8115999	0.016445
$HSe\cdot$	-2398.2423832	0.005823
CH_2=Se	-2436.8329223	0.026068
CH_3-SeH	-2438.0420402	0.047852
H_2Se	-2398.8605321	0.014538

Calculated geometries and their energies:

The geometries were optimized at B3LYP-D3/aug-cc-pVTZ and at B3LYP-D3(PCM=THF)/aug-cc-pVTZ (PCM) levels of theory and the geometries are presented in ‘xyz’ coordinates. The energies in vacuum are given at B3LYP-D3/aug-cc-pVTZ (B3LYP), DF-CCSD(T)/aug-cc-pVTZ (DF-CCSD(T)) and M06-2X/aug-cc-pVTZ (M06-2X) levels of theory. The PCM energies are given at B3LYP-D3/aug-cc-pVTZ (DFT) and M06-2X/aug-cc-pVTZ (M06-2X) levels of theory.

Gas phase geometries and their exact energies:

2*H*-pyran-2-one:

$E_{B3LYP\text{-}D3}$= -343.506338
$E_{M06\text{-}2X}$= -343.362536
$E_{DF\text{-}CCSD(T)}$= -342.837496617676

C	-0.063499	0.000000	-0.008326
C	-0.050985	0.000000	1.418937
C	1.121187	-0.000000	-0.648405
C	1.121006	0.000000	2.095251
C	2.392256	-0.000000	1.403791
O	2.300033	-0.000000	-0.007780
O	3.491720	-0.000000	1.885208
H	1.172204	0.000000	3.173051
H	-0.987847	0.000000	1.960685
H	-0.982258	0.000000	-0.572556
H	1.240825	-0.000000	-1.722273

CO_2:

$E_{B3LYP\text{-}D3}$= -188.6635649
$E_{M06\text{-}2X}$= -188.5940894
$E_{DF\text{-}CCSD(T)}$= -188.340629315003

C	0.0	0.0	0.0
O	1.160615	0.0	0.0
O	-1.160615	0.0	0.0

[PCO]⁻:

[PCO]⁻:
$E_{B3LYP-D3}$= -454.800934
E_{M06-2X}= -454.7198758
$E_{DF\_CCSD(T)}$= -454.120817541117

C	0.000000	0.000000	0.066929
P	0.000000	0.000000	1.693760
O	0.000000	0.000000	-1.130689

Product:
$E_{B3LYP-D3}$= -609.6908326
E_{M06-2X}= -609.5358757
$E_{DF-CCSD(T)}$= -608.667673614642

P	-0.412262	-0.000000	-0.225821
C	-0.101211	-0.000000	1.499561
C	1.137080	0.000000	2.117204
C	2.329797	0.000000	1.370768
C	2.400158	0.000000	-0.004653
C	1.285727	-0.000000	-0.925311
O	1.458202	-0.000000	-2.167360
H	3.377193	0.000000	-0.482018
H	3.267361	0.000000	1.920702
H	1.201958	0.000000	3.200981
H	-0.983728	-0.000000	2.135455

RP(A):

RC:
$E_{B3LYP-D3}$= -798.3301602
E_{M06-2X}= -798.1052837
$E_{DF-CCSD(T)}$= -796.982027260892

C	0.000000	0.000000	0.000000
C	0.000000	0.000000	1.358543
C	1.234126	0.000000	2.102340
O	2.396419	-0.000221	1.323488
C	2.370730	-0.000203	-0.027970
C	1.218164	-0.000088	-0.730809
O	1.365532	0.000168	3.306285
O	2.172512	0.002378	-3.900958
C	3.311372	0.002542	-3.520918
P	4.842175	0.002721	-2.984988
H	-0.907270	0.000051	1.943552
H	-0.940349	0.000066	-0.537802
H	1.256137	0.000069	-1.809561
H	3.354458	-0.000253	-0.481475

TS1:

$E_{B3LYP-D3}$= -798.2932024

E_{M06-2X}= -798.0737748

$E_{DF-CCSD(T)}$= -796.951750698228

C	0.119523	-0.116211	-0.107662
O	0.123159	-0.172279	1.278600
C	1.397356	0.032998	1.896205
C	2.044480	1.274623	1.441849
C	2.004090	1.455527	0.100423
C	1.274845	0.514318	-0.680436
P	2.562092	-1.555952	1.543390
C	2.572246	-1.529270	-0.163554
O	2.860505	-1.966837	-1.222121
O	-0.852501	-0.571232	-0.692646
H	1.302631	0.542601	-1.759199
H	2.559090	2.248947	-0.387886
H	2.630272	1.874705	2.122470
H	1.223713	-0.020851	2.967569

INT:

$E_{B3LYP-D3}$= -798.3098371

E_{M06-2X}= -798.0993381

$E_{DF-CCSD(T)}$= -796.980694614495

O	0.064138	0.137433	-0.024968
C	0.178450	-0.530813	1.297844
C	1.231530	0.205762	2.077685
C	2.388064	0.344121	1.433439
C	2.411232	-0.334637	0.089761
C	1.227429	0.204017	-0.696858
O	1.284510	0.688830	-1.805176
H	3.322174	-0.186727	-0.479766
H	3.266545	0.834822	1.826549
H	1.038999	0.551250	3.084364
H	-0.816328	-0.449110	1.723727
C	2.203270	-1.916096	0.277904
P	0.670177	-2.367886	1.096056
O	3.084180	-2.643846	-0.178896

TS2:
$E_{B3LYP-D3}$= -798.2946032
E_{M06-2X}= -798.0730448
$E_{DF-CCSD(T)}$= -796.958902632818

C	0.121102	-0.107850	-0.010992
C	0.109127	-0.099622	1.480107
C	1.638579	-0.036145	1.941520
O	2.420545	-0.888928	1.394225
C	1.296512	-2.164630	0.336921
C	0.756868	-1.140325	-0.568057
C	-0.545288	-1.386651	2.046606
O	-1.467036	-1.295711	2.848130
O	1.948179	0.836257	2.748638
P	0.269160	-2.957383	1.550089
H	-0.397676	0.755306	1.915582
H	-0.289330	0.718335	-0.574720
H	0.919870	-1.236379	-1.634571
H	2.101665	-2.770015	-0.064635

PC:
$E_{B3LYP-D3}$= -798.3669043
E_{M06-2X}= -798.1431787
$E_{DF-CCSD(T)}$= -797.021095859922

C	-0.288382	1.166532	-0.165581
P	-0.429407	-0.178550	0.945778
C	1.279536	-0.820598	0.982404
O	1.553657	-1.810744	1.719625
C	0.858196	1.578230	-0.822188
C	2.083505	0.914887	-0.645778
C	2.278397	-0.177422	0.172891
C	3.323482	-3.472062	1.479672
O	3.593151	-3.262689	0.364611
O	3.213747	-3.844649	2.577419
H	3.268905	-0.617251	0.229799
H	2.943313	1.288754	-1.194471
H	0.820431	2.431585	-1.491983
H	-1.204359	1.723713	-0.346849

RP(B):

RC:

$E_{B3LYP-D3}$= -798.3312769
E_{M06-2X}= -798.1062741
$E_{DF-CCSD(T)}$= -796.98342702958

C	0.000000	0.000000	0.000000
C	0.000000	0.000000	1.356983
C	1.233405	0.000000	2.076716
C	2.371283	-0.000291	1.362788
O	2.400138	-0.000462	0.008961
C	1.226366	-0.000055	-0.757731
O	1.352372	0.000164	-1.961848
O	-1.620863	0.017805	4.137104
C	-0.718674	0.000027	4.929478
P	0.504089	-0.010900	5.994342
H	3.366133	-0.000525	1.783418
H	1.250718	-0.000178	3.159531
H	-0.919737	0.000317	1.927111
H	-0.910874	0.000095	-0.579754

TS1:

$E_{B3LYP-D3}$= -798.2805855
E_{M06-2X}= -798.059576
$E_{DF-CCSD(T)}$= -796.938125447969

C	-0.122914	0.266274	0.113069
O	-0.009276	-0.024402	1.477109
C	1.236050	-0.048599	2.022458
C	2.317191	0.175026	1.041394
C	2.033042	1.214879	0.090476
C	0.764494	1.173792	-0.449698
O	1.365251	-0.237956	3.215900
P	2.315478	-1.799034	0.033662
C	0.808554	-1.792799	-0.731704
O	-0.062337	-2.236031	-1.417558
H	-1.129591	0.094406	-0.230837
H	0.497389	1.691019	-1.362267
H	2.832989	1.795236	-0.346236
H	3.291145	0.161232	1.511671

INT:

$E_{B3LYP-D3}$= -798.313987

E_{M06-2X}= -798.1042838

$E_{DF-CCSD(T)}$= -796.984981980080

O	0.098263	0.087728	-0.074386
C	0.022177	-0.049936	1.272696
C	1.359903	0.022997	1.956974
C	2.100824	1.218852	1.441710
C	2.159955	1.325605	0.114628
C	1.464961	0.197996	-0.587061
P	2.248643	-1.641567	1.405137
C	2.233273	-1.157820	-0.318249
O	2.728718	-1.725150	-1.298876
H	1.358518	0.323144	-1.660013
H	2.682036	2.099291	-0.429158
H	2.562320	1.916851	2.128668
H	1.216245	0.011311	3.031277
O	-1.052682	-0.197663	1.812589

TS2:

$E_{B3LYP-D3}$= -798.2802373

E_{M06-2X}= -798.0546193

$E_{DF-CCSD(T)}$= -796.940167017680

C	0.078000	-0.135827	-0.038974
C	-0.217610	-0.147079	1.347983
O	1.780937	-0.041466	2.000926
C	2.405587	-1.002233	1.488657
C	1.481239	-2.005899	0.506400
C	0.918277	-1.108752	-0.481432
C	-0.651753	-1.380599	2.069031
O	-1.641038	-1.274904	2.825244
O	3.584559	-1.330333	1.566673
P	0.337917	-2.830033	1.767459
H	-0.556124	0.765811	1.823252
H	-0.193581	0.716873	-0.647985
H	1.292725	-1.112032	-1.498576
H	2.180142	-2.729136	0.102194

PC:
$E_{B3LYP-D3}$= -798.362258
E_{M06-2X}= -798.138656
$E_{DF-CCSD(T)}$= -797.016938114864

C	0.010183	0.445610	0.122750
P	0.126623	0.030445	1.822732
C	1.943086	0.064811	2.102692
O	2.411266	-0.187259	3.236457
C	1.059433	0.711367	-0.739051
C	2.397169	0.677974	-0.300391
C	2.799036	0.388172	0.982728
C	0.203346	-2.630903	-0.640829
O	-0.916932	-2.522168	-0.935110
O	1.319785	-2.809228	-0.386468
H	3.861129	0.385877	1.214420
H	3.171253	0.894984	-1.031491
H	0.857937	0.956922	-1.777108
H	-0.997984	0.484696	-0.281884

$[PCS]^{-}$:

$[PCS]^{-}$:
$E_{B3LYP-D3}$= -777.7697123
E_{M06-2X}= -777.6822039
$E_{DF-CCSD(T)}$= -776.725876779303

C	0.000000	0.000000	0.220963
P	0.000000	0.000000	1.819500
S	0.000000	0.000000	-1.410463

Product:
$E_{B3LYP-D3}$= -932.6720784
E_{M06-2X}= -932.5130438
$E_{DF-CCSD(T)}$= -931.287114083928

P	-0.398967	-0.000000	-0.210389
C	-0.097739	-0.000000	1.512671
C	1.145369	0.000000	2.123142
C	2.326774	0.000000	1.368964
C	2.371716	0.000000	-0.013743
C	1.255731	-0.000000	-0.890335
S	1.466171	-0.000000	-2.604683
H	3.346800	0.000000	-0.491132
H	3.272780	0.000000	1.901265
H	1.217077	0.000000	3.206272
H	-0.980133	-0.000000	2.147502

RP(A):

RC:

$E_{B3LYP-D3}$= -1121.2991688
E_{M06-2X}= -1121.0679455
$E_{DF-CCSD(T)}$= -1119.58783222638

C	0.000000	0.000000	0.000000
C	0.000000	0.000000	1.356981
C	1.228495	0.000000	2.111397
O	2.399900	-0.000044	1.341020
C	2.368857	-0.000064	-0.012359
C	1.228955	-0.000023	-0.723515
O	1.357383	0.000050	3.315045
C	0.140309	0.000254	-4.111405
S	-1.357615	0.001897	-3.463475
P	1.611981	-0.001356	-4.730160
H	-0.909877	0.000000	1.938133
H	-0.920597	0.000016	-0.568483
H	1.248925	-0.000025	-1.803830
H	3.361738	-0.000063	-0.437231

TS1:

$E_{B3LYP-D3}$= -1121.2532533
E_{M06-2X}= -1121.0246085
$E_{DF-CCSD(T)}$= -1119.547446678660

C	0.052175	-0.120139	-0.098443
O	0.081128	-0.137908	1.286041
C	1.364131	-0.004892	1.883412
C	2.136103	1.114701	1.363746
C	2.100249	1.239544	0.008181
C	1.260969	0.364559	-0.714844
P	2.396446	-1.739492	1.525875
C	2.352778	-1.830365	-0.135187
S	2.636320	-2.558849	-1.548629
O	-0.975890	-0.463908	-0.653966
H	1.291355	0.310884	-1.791221
H	2.760153	1.916963	-0.520531
H	2.828208	1.646961	1.999375
H	1.213652	-0.021795	2.958115

INT:
$E_{B3LYP-D3}$= -1121.2840183
E_{M06-2X}= -1121.0683202
$E_{DF-CCSD(T)}$= -1119.593100139572

O	0.060382	0.017634	-0.053078
C	0.175315	-0.610809	1.278564
C	1.177973	0.174949	2.073200
C	2.344089	0.339960	1.454603
C	2.429340	-0.365327	0.118482
C	1.238947	0.146944	-0.693532
O	1.305024	0.661042	-1.784373
H	3.340817	-0.157450	-0.428961
H	3.193312	0.878393	1.848587
H	0.946799	0.532793	3.067250
H	-0.831358	-0.579394	1.683019
C	2.263768	-1.910192	0.299611
P	0.795824	-2.419892	1.083804
S	3.499904	-2.900804	-0.296267

TS2:
$E_{B3LYP-D3}$= -1121.264438
E_{M06-2X}= -1121.0358918
$E_{DF-CCSD(T)}$= -1119.564765236732

C	0.305475	0.033650	0.115365
O	0.170110	0.059230	1.380875
C	2.022201	-0.066410	2.205482
C	2.535710	1.140026	1.567214
C	2.483897	1.182069	0.231878
C	1.843566	0.011864	-0.430052
P	2.558094	-1.684151	1.681235
C	2.482677	-1.312770	-0.053462
S	2.946099	-2.394663	-1.249235
O	-0.570293	0.064976	-0.740350
H	1.778787	0.104857	-1.507732
H	2.770934	2.047073	-0.349282
H	2.871630	1.969060	2.177116
H	1.731125	0.027019	3.245844

PC:
$E_{B3LYP-D3}$= -1121.344593
E_{M06-2X}= -1121.1168784
$E_{DF-CCSD(T)}$= -1119.63741563307

C	-0.209658	0.779651	-0.153174
P	-0.133668	0.120511	1.464932
C	1.609402	-0.210025	1.659487
S	2.174485	-0.899587	3.146245
C	0.864097	0.962595	-1.008895
C	2.167861	0.622499	-0.627230
C	2.506427	0.085568	0.603051
C	2.872722	-3.373132	1.095051
O	3.954843	-3.001318	0.889741
O	1.814829	-3.832014	1.231107
H	3.551196	-0.140514	0.788794
H	2.969548	0.789678	-1.339260
H	0.702284	1.378618	-1.998333
H	-1.198386	1.063599	-0.503771

RP(B):

RC:
$E_{B3LYP-D3}$= -1121.2977795
E_{M06-2X}= -1121.067267
$E_{DF-CCSD(T)}$= -1119.58544701909

O	0.000000	0.000000	0.000000
C	0.000000	0.000000	1.402490
C	1.303861	0.000000	2.010601
C	2.433713	0.002725	1.252089
C	2.360099	0.004378	-0.162950
C	1.135110	0.001762	-0.728021
O	-1.075468	0.000201	1.959959
C	3.004207	-0.210631	-3.613690
S	2.950702	1.418086	-3.572797
P	3.035017	-1.806585	-3.614692
H	0.960105	0.001228	-1.792890
H	3.225033	0.004333	-0.805800
H	3.403729	0.003105	1.734244
H	1.325337	-0.002114	3.089778

TS1:

$E_{B3LYP\text{-}D3}$= -1121.2485812
$E_{M06\text{-}2X}$= -1121.0221265
$E_{DF\text{-}CCSD(T)}$= -1119.544226057297

C	-0.005512	0.143959	0.067831
C	0.049064	-0.016263	1.475817
C	1.302823	0.023296	2.044398
C	2.359330	0.295278	1.176200
O	2.142194	1.154503	0.110812
C	0.925716	1.111443	-0.515104
C	2.535411	-1.697073	0.067531
S	3.999190	-2.313535	0.508503
O	0.716263	1.850633	-1.454195
P	1.130813	-1.748701	-0.798231
H	3.394621	0.302686	1.472186

INT:

$E_{B3LYP\text{-}D3}$= -1121.2914596
$E_{M06\text{-}2X}$= -1121.0763162
$E_{DF\text{-}CCSD(T)}$= -1119.600583266899

C	0.012996	-0.021532	-0.006009
C	0.018863	-0.027013	1.500305
O	1.420207	-0.006826	1.953315
C	2.178985	-1.008270	1.456997
C	1.445120	-1.874580	0.466915
C	0.760433	-0.990025	-0.529567
C	-0.680991	-1.302595	2.022346
S	-2.112202	-1.101125	2.910594
O	3.331911	-1.144376	1.803119
P	0.107013	-2.785722	1.586420
H	-0.424732	0.854366	1.948766
H	-0.579159	0.689442	-0.561898
H	0.872514	-1.169296	-1.590916
H	2.127875	-2.600197	0.040040

TS2:

$E_{B3LYP-D3}$= -1121.2631012
E_{M06-2X}= -1121.0323882
$E_{DF-CCSD(T)}$= -1119.560307163345

C	0.021128	-0.005440	-0.060881
C	0.018679	0.058333	1.390955
C	1.213792	-0.006246	2.024468
C	2.357136	-0.238405	1.199221
O	2.015399	1.450068	-0.065711
C	0.872487	1.336656	-0.568576
C	2.365473	-1.311561	0.215471
S	3.728502	-2.351861	0.287936
O	0.262552	2.046005	-1.361964
P	1.000219	-1.426583	-0.842958
H	3.327741	0.099008	1.536588
H	1.342503	0.261641	3.064267
H	-0.891265	0.309036	1.923464
H	-0.959207	0.082154	-0.515144

PC:

$E_{B3LYP-D3}$= -1121.342596
E_{M06-2X}= -1121.1145799
$E_{DF-CCSD(T)}$= -1119.6354653473

C	0.012277	0.501306	0.132942
P	0.116046	0.121755	1.839426
C	1.882575	0.095101	2.118541
S	2.479724	-0.244405	3.700914
C	1.081479	0.710558	-0.721969
C	2.405247	0.633898	-0.265361
C	2.767245	0.351821	1.038190
C	0.211089	-2.608085	-0.613419
O	-0.904361	-2.498822	-0.925598
O	1.323021	-2.775115	-0.334623
H	3.825708	0.314700	1.276079
H	3.202675	0.802994	-0.981762
H	0.901936	0.940483	-1.767498
H	-0.991626	0.566776	-0.277041

[PCSe]⁻:

[PCSe]⁻:

$E_{B3LYP-D3}$= -2781.1748423
E_{M06-2X}= -2781.112811
$E_{DF-CCSD(T)}$= -2779.040736054697

C	0.000000	0.000000	0.000000
P	-1.591676	0.000000	0.000000
Se	1.786417	0.000000	0.000000

Product:
$E_{B3LYP-D3}$= -2936.0828987
E_{M06-2X}= -2935.9476684
$E_{DF-CCSD(T)}$= -2933.607218002151

C	-0.042675	0.043481	0.020542
C	0.343117	-0.512019	1.230233
C	1.429795	-0.088462	2.026003
P	2.448120	1.271183	1.505507
C	1.708524	1.727764	-0.012275
C	0.604459	1.123462	-0.591331
Se	1.824819	-0.957594	3.660205
H	-0.235073	-1.348796	1.609220
H	-0.902412	-0.389621	-0.480232
H	0.225000	1.488284	-1.540775
H	2.169246	2.562226	-0.534374

RP(A):

RC:
$E_{B3LYP-D3}$= -3124.70263
E_{M06-2X}= -3124.495828
$E_{DF-CCSD(T)}$= -3121.8997512808

C	0.000000	0.000000	0.000000
C	0.000000	0.000000	1.360695
C	1.215998	0.000000	2.088311
C	2.367724	-0.002021	1.386067
O	2.396222	-0.002676	0.038000
C	1.231629	-0.000238	-0.743841
O	1.368156	0.001596	-1.947300
C	3.729772	-0.412441	4.559613
Se	3.743247	1.370955	4.633886
P	3.698745	-1.998456	4.455743
H	3.349531	-0.002988	1.834291
H	1.267353	0.002788	3.164862
H	-0.940945	-0.000066	1.897314
H	-0.907962	0.000840	-0.583685

TS1:
$E_{B3LYP-D3}$= -3124.6568569
E_{M06-2X}= -3124.4506993
$E_{DF-CCSD(T)}$= -3121.860002807668

C	-0.029037	-0.007577	0.042191
C	0.073929	0.081249	1.481302
O	1.372732	-0.143096	1.991859
C	2.043963	-1.253859	1.506636
C	1.454651	-1.842247	0.329163
C	0.658238	-1.048848	-0.514661
P	-1.150896	-1.412501	2.298644
C	-0.371018	-2.790239	1.830128
Se	-0.129656	-4.545144	1.794781
O	3.066733	-1.594621	2.069879
H	-0.307953	0.982390	1.948192
H	-0.771175	0.569976	-0.488804
H	0.517940	-1.332840	-1.550432
H	1.905200	-2.760357	-0.011085

INT:
$E_{B3LYP-D3}$= -3124.6927164
E_{M06-2X}= -3124.5001721
$E_{DF-CCSD(T)}$= -3121.910988114054

C	-0.135032	-0.125605	-0.039510
C	0.063000	-0.020434	1.500406
C	1.566356	-0.054777	1.786606
O	2.147973	-1.222808	1.447468
C	1.213032	-2.206706	0.870703
P	0.532435	-1.525756	-0.798687
C	-0.505396	-1.241317	2.193227
C	0.073251	-2.383121	1.830938
O	2.201852	0.834383	2.298720
Se	-1.045645	1.254981	-0.875692
H	1.811395	-3.099918	0.722773
H	-0.239082	-3.367045	2.152909
H	-1.332486	-1.148104	2.881240
H	-0.338244	0.915524	1.869764

TS2:

$E_{B3LYP-D3}$= -3124.6717018
E_{M06-2X}= -3124.4655304
$E_{DF-CCSD(T)}$= -3121.880543604669

C	0.246811	0.051728	0.023459
C	-0.079805	0.116114	1.439441
O	1.849960	0.026956	2.089209
C	2.362652	-1.047777	1.645399
C	1.384990	-1.953857	0.684416
C	0.961325	-1.005689	-0.380620
P	-0.806306	-1.284446	2.279242
C	0.239716	-2.477167	1.518083
Se	0.073944	-4.282301	1.819824
O	3.498146	-1.475843	1.804756
H	-0.326185	1.095283	1.834965
H	-0.021952	0.872480	-0.629350
H	1.329104	-1.124576	-1.390177
H	2.005030	-2.764908	0.321731

PC:

$E_{B3LYP-D3}$= -3124.7552974
E_{M06-2X}= -3124.5512542
$E_{DF-CCSD(T)}$= -3121.95731422958

C	0.000000	0.000000	0.000000
C	0.000000	0.000000	1.385703
C	1.191588	0.000000	2.118343
C	2.455472	-0.002965	1.547999
C	2.734668	-0.011963	0.165342
Se	4.527411	-0.042506	-0.455387
P	1.418028	-0.008563	-1.023410
O	4.233420	-2.854397	1.944248
C	3.838049	-3.153089	0.893010
O	3.424941	-3.545230	-0.118600
H	-0.958051	0.002388	-0.512708
H	-0.942195	0.001365	1.924801
H	1.128573	0.001284	3.201375
H	3.310837	-0.006960	2.215066

RP(B):

RC:

$E_{B3LYP-D3}$= -3124.7026301
E_{M06-2X}= -3124.4958251
$E_{DF-CCSD(T)}$= -3121.89974893148

C	0.000000	0.000000	0.000000
C	0.000000	0.000000	1.417089
C	1.193973	0.000000	2.044869
O	2.365365	0.001111	1.377099
C	2.438286	0.003324	-0.023682
C	1.167628	0.001778	-0.698681
O	3.541099	0.006457	-0.524472
C	-0.827827	-0.392857	4.845674
P	-0.757294	-1.979407	4.773714
Se	-0.881895	1.390791	4.886619
H	1.313382	-0.000468	3.117537
H	-0.897447	0.001568	2.013915
H	-0.943635	-0.001449	-0.531885
H	1.202306	0.002442	-1.777516

TS1:

$E_{B3LYP-D3}$= -3124.6555952
E_{M06-2X}= -3124.4531476
$E_{DF-CCSD(T)}$= -3121.861108295393

P	-0.021806	0.273457	-0.144689
C	0.021834	-0.037887	2.333001
C	1.475363	0.069026	2.347177
O	2.121977	-1.027290	1.831589
C	1.358879	-2.049164	1.299039
C	-0.544277	-1.320285	2.473147
C	0.147486	-2.373107	1.918392
O	2.134969	0.992558	2.776009
H	1.982570	-2.776369	0.806940
H	-0.290037	-3.346694	1.758620
H	-1.562246	-1.430069	2.821887
H	-0.490808	0.839487	2.697320
C	0.646769	-1.154779	-0.592467
Se	1.114696	-2.342445	-1.888655

INT:
$E_{B3LYP-D3}$= -3124.7006275
E_{M06-2X}= -3124.5086691
$E_{DF-CCSD(T)}$= -3121.918953058407

C	0.025276	-0.017700	-0.013100
C	0.039678	-0.013730	1.493773
O	1.447761	0.033344	1.934603
C	2.218483	-0.957588	1.440118
C	1.491339	-1.843720	0.460438
C	0.785843	-0.975989	-0.536440
C	-0.626778	-1.296382	2.022690
Se	-2.193748	-1.100812	2.996640
O	3.376061	-1.074645	1.776912
P	0.171124	-2.761111	1.597910
H	-0.409814	0.866414	1.938151
H	-0.582323	0.680530	-0.568220
H	0.894940	-1.159109	-1.597231
H	2.182364	-2.561801	0.034018

TS2:
$E_{B3LYP-D3}$= -3124.6741921
E_{M06-2X}= -3124.466758
$E_{DF-CCSD(T)}$= -3121.880342151629

P	0.099934	0.270322	-0.260949
C	0.056360	-0.004881	1.614133
C	1.667081	-0.026834	2.017307
O	2.297591	-1.024294	1.590775
C	0.957930	-2.216852	0.427090
C	0.665741	-1.304177	-0.658343
C	-0.486976	-1.283552	2.052380
C	0.013555	-2.405978	1.488454
O	2.032677	0.931565	2.690509
Se	0.873013	-2.058222	-2.363928
H	1.690974	-2.986870	0.230156
H	-0.156209	-3.394966	1.890975
H	-1.152336	-1.327011	2.906499
H	-0.362470	0.857435	2.120297

PC:
$E_{B3LYP-D3}$= -3124.7552974
E_{M06-2X}= -3124.5512543
$E_{DF-CCSD(T)}$= -3121.95731421174

C	0.000000	0.000000	0.000000
C	0.000000	0.000000	1.385702
C	1.191588	0.000000	2.118342
C	2.455471	-0.002965	1.547998
C	2.734667	-0.011963	0.165342
Se	4.527410	-0.042506	-0.455387
P	1.418029	-0.008563	-1.023411
O	4.233431	-2.854399	1.944189
C	3.838051	-3.153077	0.892950
O	3.424935	-3.545204	-0.118661
H	-0.958051	0.002388	-0.512708
H	-0.942194	0.001365	1.924800
H	1.128573	0.001284	3.201375
H	3.310835	-0.006960	2.215065

$[AsCO]^-$:

$[AsCO]^-$:
$E_{B3LYP-D3}$= -2349.3788366
E_{M06-2X}= -2349.3044273
$E_{DF-CCSD(T)}$= -2347.596819853372

C	0.000000	0.000000	-0.099567
O	0.000000	0.000000	1.091132
As	0.000000	0.000000	-1.855364

Product:
$E_{B3LYP-D3}$= -2504.2650721
E_{M06-2X}= -2504.115417
$E_{DF-CCSD(T)}$= -2502.140128099706

As	-0.487475	0.000000	-0.298936
C	-0.122632	0.000000	1.543066
C	1.128940	0.000000	2.121043
C	2.321976	0.000000	1.368531
C	2.428137	0.000000	-0.004453
C	1.360497	0.000000	-0.978297
O	1.587446	0.000000	-2.204881
H	3.423448	0.000000	-0.444684
H	3.253295	0.000000	1.929164
H	1.218030	0.000000	3.204375
H	-0.987894	0.000000	2.201136

RP(A):

RC:

$E_{B3LYP-D3}$= -2692.9071559
E_{M06-2X}= -2692.6887626
$E_{DF-CCSD(T)}$= -2690.457090842320

C	0.000000	0.000000	0.000000
C	0.000000	0.000000	1.358460
C	1.233696	0.000000	2.102761
O	2.396865	-0.000376	1.323735
C	2.370876	-0.000338	-0.027315
C	1.218508	-0.000113	-0.730062
O	1.365091	0.000269	3.306403
O	2.028315	-0.001582	-4.002096
C	3.169574	-0.001926	-3.652546
As	4.834587	-0.002399	-3.112321
H	-0.907338	0.000085	1.943289
H	-0.940051	0.000082	-0.538155
H	1.253816	-0.000158	-1.808057
H	3.354304	-0.000574	-0.480952

TS1:

$E_{B3LYP-D3}$= -2692.8721855
E_{M06-2X}= -2692.6580595
$E_{DF-CCSD(T)}$= -2690.427739304957

C	0.158226	-0.176602	-0.115347
O	0.148997	-0.219112	1.271977
C	1.392344	0.027220	1.916630
C	2.059310	1.236455	1.441079
C	2.032776	1.404759	0.094776
C	1.308693	0.464524	-0.686329
As	2.669452	-1.690858	1.584115
C	2.646693	-1.589245	-0.258472
O	2.918546	-1.982435	-1.333730
O	-0.803085	-0.648953	-0.702645
H	1.336852	0.493457	-1.764986
H	2.608161	2.183184	-0.393309
H	2.665228	1.827745	2.111976
H	1.207248	-0.023644	2.984644

INT:

$E_{B3LYP-D3}$= -2692.8891167
E_{M06-2X}= -2692.6846657
$E_{DF-CCSD(T)}$= -2690.457027083643

C	0.104790	-0.099721	-0.034917
O	0.068066	-0.127161	1.309773
C	1.381757	0.009157	1.966035
C	2.044028	1.240275	1.435589
C	2.058684	1.345265	0.107301
C	1.489876	0.149861	-0.606996
As	2.561656	-1.617246	1.541979
C	2.402718	-1.144527	-0.329081
O	2.868608	-1.707883	-1.310716
O	-0.906917	-0.225880	-0.688059
H	1.414747	0.261575	-1.683559
H	2.502877	2.161076	-0.444054
H	2.498727	1.953570	2.109988
H	1.155902	0.047592	3.025926

TS2:

$E_{B3LYP-D3}$= -2692.8691764
E_{M06-2X}= -2692.6522546
$E_{DF-CCSD(T)}$= -2690.430521480635

C	0.119333	-0.123675	-0.009864
C	0.106674	-0.095403	1.478278
C	1.638679	-0.035721	1.958737
O	2.432116	-0.865808	1.404666
C	1.325695	-2.182847	0.286198
C	0.759720	-1.149923	-0.578127
C	-0.575422	-1.348553	2.078989
O	-1.487823	-1.223498	2.877408
O	1.920377	0.823775	2.791090
As	0.267640	-3.071943	1.583363
H	-0.388506	0.776736	1.894090
H	-0.296302	0.697988	-0.576709
H	0.911250	-1.222417	-1.649070
H	2.148846	-2.752454	-0.130207

PC:

$E_{B3LYP-D3}$= -2692.9358202
E_{M06-2X}= -2692.7168001
$E_{DF-CCSD(T)}$= -2690.488582009361

C	0.000000	0.000000	0.000000
C	0.000000	0.000000	1.379890
C	1.182025	0.000000	2.145747
C	2.474081	0.016159	1.663359
C	2.905850	0.060903	0.286006
O	4.113755	0.126066	-0.022499
As	1.515080	0.022399	-1.104491
C	2.750996	3.124127	1.911270
O	2.069467	3.399721	1.015294
O	3.438022	2.915978	2.826249
H	3.290968	0.012273	2.381047
H	1.066549	-0.016757	3.226358
H	-0.946126	-0.007877	1.914482
H	-0.960586	-0.010417	-0.508412

RP(B):

RC:

$E_{B3LYP-D3}$= -2692.908288
E_{M06-2X}= -2692.6903033
$E_{DF-CCSD(T)}$= -2690.458061207342

C	0.000000	0.000000	0.000000
C	0.000000	0.000000	1.426945
C	1.186178	0.000000	2.055657
O	2.373537	-0.002019	1.401607
C	2.444945	0.000027	0.000823
C	1.174712	0.000789	-0.679564
O	3.550190	0.001299	-0.494172
O	-2.849292	1.495595	0.605830
C	-3.252755	0.375263	0.655407
As	-3.760896	-1.297740	0.724115
H	1.322078	-0.001171	3.127201
H	-0.929810	-0.018360	1.972873
H	-0.951300	-0.013566	-0.512638
H	1.219039	0.000412	-1.758209

TS1:

$E_{B3LYP-D3}$= -2692.8633635
E_{M06-2X}= -2692.6478406
$E_{DF-CCSD(T)}$= -2690.418165246957

C	-0.090678	0.195853	0.099076
O	0.011483	-0.061719	1.468496
C	1.253848	-0.050905	2.034226
C	2.337796	0.249273	1.092922
C	2.036327	1.209384	0.084461
C	0.774895	1.129628	-0.465702
O	1.363370	-0.260438	3.226427
As	2.345905	-1.948416	0.023894
C	0.712999	-1.797553	-0.791220
O	-0.166456	-2.183502	-1.496656
H	-1.098898	0.024364	-0.242976
H	0.508192	1.619048	-1.392696
H	2.825348	1.792174	-0.369729
H	3.312226	0.249588	1.559154

INT:

$E_{B3LYP-D3}$= -2692.8960173
E_{M06-2X}= -2692.6922208
$E_{DF-CCSD(T)}$= -2690.463781809808

C	-0.001571	-0.044298	-0.016523
O	0.010242	-0.064323	1.442229
C	1.246438	-0.047288	2.007972
C	2.364231	0.090009	1.023825
C	2.041789	1.146729	0.025827
C	0.820202	1.106506	-0.508533
O	1.359144	-0.114490	3.213292
As	2.360025	-1.797034	0.080109
C	0.582689	-1.405716	-0.570027
O	-0.127302	-2.053722	-1.335538
H	-1.051734	0.002234	-0.290777
H	0.450467	1.780081	-1.267345
H	2.789424	1.881997	-0.244702
H	3.304532	0.218625	1.545391

TS2:
$E_{B3LYP-D3}$= -2692.8589991
E_{M06-2X}= -2692.6378924
$E_{DF-CCSD(T)}$= -2690.415920162324

C	0.073373	-0.144480	-0.035655
C	-0.239422	-0.145406	1.344415
O	1.793001	-0.059202	2.014510
C	2.419932	-1.014596	1.496369
C	1.534125	-1.990949	0.470883
C	0.923607	-1.106654	-0.489316
C	-0.697345	-1.349140	2.095534
O	-1.675997	-1.210301	2.850417
O	3.598237	-1.347796	1.590964
As	0.335108	-2.935618	1.808409
H	-0.568294	0.780855	1.802224
H	-0.204086	0.705308	-0.646676
H	1.264788	-1.104735	-1.518829
H	2.242248	-2.692893	0.047602

PC:
$E_{B3LYP-D3}$= -2692.9366455
E_{M06-2X}= -2692.7182108
$E_{DF-CCSD(T)}$= -2690.489271960145

C	0.000000	0.000000	0.000000
C	0.000000	0.000000	1.378112
C	1.184073	0.000000	2.146558
C	2.473418	-0.000212	1.667900
C	2.909686	0.002466	0.289445
O	4.117022	0.002634	-0.018210
As	1.520159	0.006951	-1.106256
C	0.000841	3.179933	-0.003293
O	0.899945	3.355824	0.706632
O	-0.923364	3.083212	-0.703334
H	3.290469	0.003724	2.386322
H	1.065013	0.003609	3.226745
H	-0.946454	-0.002825	1.912502
H	-0.959707	0.005547	-0.508975

$[AsCS]^{-}$:

$[AsCS]^{-}$:
$E_{B3LYP-D3}$= -2672.3438073
E_{M06-2X}= -2672.2613138
$E_{DF-CCSD(T)}$= -2670.198512504877

C	-0.004268	0.000000	-0.105679
S	0.000429	0.000000	1.517908
As	-0.014771	0.000000	-1.825952

Product:
$E_{B3LYP-D3}$= -2827.2442209
E_{M06-2X}= -2827.090179
$E_{DF-CCSD(T)}$= -2824.757850700537

C	-0.018206	0.040384	0.042970
C	0.351006	-0.527489	1.249259
C	1.423039	-0.145037	2.095110
As	2.535472	1.323728	1.571006
C	1.707654	1.769761	-0.051673
C	0.611126	1.127203	-0.587941
S	1.736785	-0.970981	3.570312
H	-0.241537	-1.366956	1.603504
H	-0.874723	-0.397543	-0.460361
H	0.205309	1.463982	-1.538262
H	2.136995	2.602856	-0.601201

RP(A):

RC:
$E_{B3LYP-D3}$= -3015.871725
E_{M06-2X}= -3015.645929
$E_{DF-CCSD(T)}$= -3013.057649850234

C	0.000000	0.000000	0.000000
C	0.000000	0.000000	1.360986
C	1.215708	0.000000	2.088630
C	2.367730	0.001438	1.386426
O	2.396202	0.002849	0.038424
C	1.231478	0.001865	-0.743679
O	1.368578	0.003050	-1.947179
C	3.728166	0.026174	4.565413
S	3.705035	1.635114	4.359829
As	3.730712	-1.683107	4.739074
H	3.349473	0.002097	1.834598
H	1.267187	-0.004981	3.165311
H	-0.941028	0.000016	1.897521
H	-0.907961	-0.000951	-0.583685

TS1:

$E_{B3LYP-D3}$= -3015.8293727

E_{M06-2X}= -3015.6056377

$E_{DF-CCSD(T)}$= -3013.021735278880

C	-0.041871	-0.031487	0.054275
C	0.062547	0.078222	1.490918
O	1.356977	-0.162003	2.005682
C	2.019257	-1.281388	1.530296
C	1.422040	-1.879820	0.361665
C	0.641284	-1.076754	-0.495231
As	-1.247363	-1.487061	2.361768
C	-0.338233	-2.917931	1.782013
S	-0.066379	-4.507222	1.699808
O	3.038860	-1.625488	2.098670
H	-0.310465	0.985663	1.951252
H	-0.776144	0.549853	-0.484019
H	0.506479	-1.362170	-1.531252
H	1.883863	-2.791009	0.017653

INT:

$E_{B3LYP-D3}$= -3015.8605825

E_{M06-2X}= -3015.6503059

$E_{DF-CCSD(T)}$= -3013.067076632198

C	-0.170994	-0.079260	-0.035298
C	0.069198	-0.023389	1.507560
C	1.574398	-0.057225	1.776124
O	2.157569	-1.230662	1.462554
C	1.244410	-2.242817	0.915340
As	0.537314	-1.581113	-0.904523
C	-0.500810	-1.245929	2.191821
C	0.083113	-2.390481	1.843347
O	2.214165	0.843604	2.264011
S	-1.003315	1.203651	-0.740145
H	1.850359	-3.135499	0.805687
H	-0.251808	-3.370123	2.157301
H	-1.348929	-1.156827	2.854417
H	-0.323738	0.909482	1.895735

TS2:

$E_{B3LYP-D3}$= -3015.8365353

E_{M06-2X}= -3015.61281

$E_{DF-CCSD(T)}$= -3013.034495933275

C	0.233022	0.051838	0.025127
C	-0.090377	0.174131	1.434185
O	1.871247	0.031804	2.076968
C	2.355256	-1.065108	1.661948
C	1.374067	-1.962882	0.697337
C	0.932159	-1.021565	-0.365585
As	-0.894612	-1.266463	2.385789
C	0.246502	-2.537373	1.528622
S	0.134060	-4.185748	1.776779
O	3.474271	-1.527885	1.850331
H	-0.300421	1.173953	1.797349
H	-0.029890	0.855084	-0.652998
H	1.279430	-1.157688	-1.380422
H	1.998899	-2.765308	0.321096

PC:

$E_{B3LYP-D3}$= -3015.916582

E_{M06-2X}= -3015.693822

$E_{DF-CCSD(T)}$= -3013.108097072428

C	0.000000	0.000000	0.000000
C	0.000000	0.000000	1.380100
C	1.179200	0.000000	2.142400
C	2.471100	0.000800	1.644000
C	2.870700	-0.002700	0.285400
S	4.538000	-0.015100	-0.157300
As	1.532400	-0.007000	-1.079700
C	4.144100	-3.058600	1.085800
O	3.852300	-3.483000	0.045300
O	4.427900	-2.731400	2.164200
H	-0.956200	0.000500	-0.515400
H	-0.946300	0.000700	1.914500
H	1.073200	-0.000500	3.222600
H	3.281000	-0.001600	2.367600

RP(B):

RC:

$E_{B3LYP-D3}$= -3015.8727419
E_{M06-2X}= -3015.646497
$E_{DF-CCSD(T)}$= -3013.059678238143

C	0.000000	0.000000	0.000000
C	0.000000	0.000000	1.441660
C	1.161277	0.000000	2.145287
C	2.417855	-0.000128	1.479597
C	2.408576	-0.000373	0.133592
O	1.267388	-0.000281	-0.594013
O	-0.961927	0.000185	-0.735581
C	5.850189	-0.048213	2.330997
S	6.082350	-0.035214	0.724237
As	5.574691	-0.061375	4.027358
H	3.299013	-0.001660	-0.476743
H	3.354938	-0.002426	2.016876
H	1.142626	-0.000441	3.227862
H	-0.970295	-0.000190	1.914968

TS1:

$E_{B3LYP-D3}$= -3015.828319
E_{M06-2X}= -3015.6061708
$E_{DF-CCSD(T)}$= -3013.021375706627

C	-0.010014	0.081833	-0.021365
C	0.047863	0.017152	1.353119
C	1.308450	0.201064	1.925354
O	2.197249	1.065365	1.310174
C	2.210351	1.126317	-0.061828
C	1.205428	0.290443	-0.706773
C	2.267775	-1.812194	1.591660
As	2.528358	-1.847688	-0.166950
O	3.008116	1.860807	-0.607452
S	2.406728	-2.483922	3.086925
H	1.489554	0.157755	2.986188
H	-0.763261	-0.360532	1.957355
H	-0.897728	-0.209102	-0.566465
H	1.236336	0.315599	-1.785499

INT:

$E_{B3LYP-D3}$= -3015.8704227
E_{M06-2X}= -3015.6603637
$E_{DF-CCSD(T)}$= -3013.076742315610

C	0.011679	-0.030779	-0.001620
C	0.031920	-0.010601	1.503069
O	1.431482	0.014002	1.950738
C	2.213153	-0.971693	1.451030
C	1.525106	-1.834934	0.438214
C	0.777135	-0.984634	-0.529263
C	-0.690325	-1.257509	2.062977
S	-2.116001	-1.012970	2.930364
O	3.364032	-1.085096	1.814875
As	0.142466	-2.877126	1.645441
H	-0.402420	0.885900	1.932410
H	-0.615602	0.648567	-0.558359
H	0.856838	-1.173261	-1.592283
H	2.228876	-2.532069	0.000346

TS2:

$E_{B3LYP-D3}$= -3015.8398173
E_{M06-2X}= -3015.6131311
$E_{DF-CCSD(T)}$= -3013.034428486717

As	0.087880	0.359206	-0.338291
C	0.049644	-0.000577	1.661024
C	1.668680	-0.016488	2.010786
O	2.301512	-1.006040	1.572628
C	0.942850	-2.234131	0.407331
C	0.678063	-1.362974	-0.724143
C	-0.489306	-1.283908	2.055673
C	0.011296	-2.402297	1.475341
O	2.039575	0.946847	2.677561
S	0.915962	-2.062433	-2.264465
H	1.666302	-3.019092	0.227089
H	-0.149141	-3.389362	1.888105
H	-1.160216	-1.350471	2.904952
H	-0.366236	0.847406	2.191766

PC:
$E_{B3LYP-D3}$= -3015.9149364
E_{M06-2X}= -3015.691806
$E_{DF-CCSD(T)}$= -3013.106089644210

C	0.000000	0.000000	0.000000
As	0.000000	0.000000	1.878083
C	1.891131	0.000000	2.186412
S	2.491627	0.004708	3.794384
C	1.126509	-0.002851	-0.795728
C	2.430748	-0.006048	-0.269204
C	2.768352	-0.004764	1.070973
C	0.087748	-3.213188	0.039953
O	1.195850	-3.340085	0.353560
O	-1.024824	-3.152703	-0.295043
H	3.827079	-0.011225	1.315393
H	3.251710	-0.013748	-0.979245
H	1.017951	-0.002461	-1.877047
H	-0.972413	-0.004795	-0.483196

$[AsCSe]^{-}$:

$[AsCSe]^{-}$:
$E_{B3LYP-D3}$= -4675.7481246
E_{M06-2X}= -4675.6889405
$E_{DF-CCSD(T)}$= -4672.512563044197

C	0.000000	0.000000	-0.163346
Se	0.000000	0.000000	1.615512
As	0.000000	0.000000	-1.875765

Product:
$E_{B3LYP-D3}$= -4830.6544449
E_{M06-2X}= -4830.5239968
$E_{DF-CCSD(T)}$= -4827.077488296311

C	-0.024150	0.048490	0.032556
C	0.354914	-0.523957	1.236840
C	1.427623	-0.137198	2.067926
As	2.534929	1.324317	1.556755
C	1.700472	1.780024	-0.059419
C	0.601205	1.138192	-0.593087
Se	1.778966	-1.052872	3.677284
H	-0.235138	-1.365897	1.588275
H	-0.882887	-0.387809	-0.467713
H	0.190787	1.480780	-1.539328
H	2.126200	2.615839	-0.607366

RP(A):

RC:

$E_{B3LYP-D3}$= -5019.3267631
E_{M06-2X}= -5019.073123
$E_{DF-CCSD(T)}$= -5015.371236967499

C	0.000000	0.000000	0.000000
C	0.000000	0.000000	1.360814
C	1.215909	0.000000	2.088290
C	2.367836	-0.000834	1.386116
O	2.396308	0.000074	0.038162
C	1.231505	0.001133	-0.743817
O	1.368278	0.003225	-1.947234
C	3.741460	-0.290781	4.581340
Se	3.756761	1.486291	4.576293
As	3.706788	-2.000383	4.544026
H	3.349774	-0.002009	1.834067
H	1.266659	-0.001301	3.164827
H	-0.940901	-0.000642	1.897473
H	-0.907985	-0.000824	-0.583632

TS1:

$E_{B3LYP-D3}$= -5019.2328341
E_{M06-2X}= -5019.0314202
$E_{DF-CCSD(T)}$= -5015.334389959805

C	0.036055	0.112397	0.008646
C	0.139220	0.165815	1.437608
O	1.414733	-0.104464	1.962895
C	2.102410	-1.183479	1.431754
C	1.525297	-1.727746	0.224278
C	0.738439	-0.897227	-0.591566
As	-1.175324	-1.521973	2.214182
C	-0.234552	-2.879653	1.558473
Se	0.098422	-4.610066	1.352462
O	3.124190	-1.536906	1.985928
H	-0.266250	1.026304	1.954737
H	-0.718748	0.692857	-0.501397
H	0.598621	-1.140708	-1.637128
H	2.002943	-2.613540	-0.160854

INT:
$E_{B3LYP-D3}$= -5019.2685871
E_{M06-2X}= -5019.0810501
$E_{DF-CCSD(T)}$= -5015.384397804325

C	-0.154062	-0.067576	-0.007378
C	0.077662	-0.021352	1.529948
C	1.583598	-0.066491	1.800207
O	2.160719	-1.239733	1.470950
C	1.242908	-2.243903	0.922315
As	0.532803	-1.551819	-0.890686
C	-0.496984	-1.248465	2.204893
C	0.081980	-2.393272	1.849072
O	2.228381	0.823813	2.298640
Se	-1.057985	1.347560	-0.771923
H	1.844433	-3.138348	0.803024
H	-0.257134	-3.373587	2.156127
H	-1.343767	-1.159542	2.869169
H	-0.309455	0.907861	1.932137

TS2:
$E_{B3LYP-D3}$= -5019.2432025
E_{M06-2X}= -5019.041655
$E_{DF-CCSD(T)}$= -5015.349755359635

C	0.234828	0.050117	0.028293
C	-0.098476	0.121505	1.434748
O	1.862890	0.031554	2.094299
C	2.398329	-1.022614	1.640215
C	1.442845	-1.944715	0.649965
C	0.976037	-0.987717	-0.385414
As	-0.845573	-1.383009	2.343094
C	0.347193	-2.568256	1.473368
Se	0.305982	-4.381679	1.723897
O	3.538597	-1.439522	1.795674
H	-0.346015	1.101581	1.826884
H	-0.042808	0.865795	-0.628615
H	1.342856	-1.077179	-1.398609
H	2.104634	-2.707507	0.255646

PC:
$E_{B3LYP-D3}$= -5019.3267631
E_{M06-2X}= -5019.127458
$E_{DF-CCSD(T)}$= -5015.427461954292

C	-0.217279	-0.711621	-0.172098
As	-0.143681	-0.123498	1.605969
C	1.734511	0.119437	1.763296
Se	2.456390	0.737278	3.396981
C	0.875165	-0.864715	-1.002533
C	2.189544	-0.596420	-0.593218
C	2.573395	-0.152869	0.663780
C	2.859630	3.284432	1.079421
O	3.927335	2.935827	0.781147
O	1.804773	3.708201	1.314427
H	-1.202165	-0.942151	-0.567754
H	0.725063	-1.208782	-2.022401
H	2.979944	-0.747634	-1.321124
H	3.634680	0.010883	0.824078

RP(B):

RC:
$E_{B3LYP-D3}$= -5019.2758932
E_{M06-2X}= -5019.073123
$E_{DF-CCSD(T)}$= -5015.371233312123

C	0.000000	0.000000	0.000000
C	0.000000	0.000000	1.438662
C	1.164822	0.000000	2.142270
C	2.416161	-0.001104	1.477622
C	2.410674	-0.002774	0.128534
O	1.271640	-0.001787	-0.592761
O	-0.959332	0.001725	-0.739320
C	5.856317	-0.281910	0.603027
Se	5.855022	1.495117	0.587696
As	5.811251	-1.991663	0.612995
H	3.301844	-0.004757	-0.480314
H	3.363898	-0.002340	1.990768
H	1.137695	0.000213	3.225128
H	-0.969019	-0.000111	1.914135

TS1:

$E_{B3LYP-D3}$= -5019.2346225
E_{M06-2X}= -5019.0354326
$E_{DF-CCSD(T)}$= -5015.354000750539

As	0.077879	0.361038	-0.321679
C	0.057437	0.003222	1.679166
C	1.670732	-0.009730	2.024020
O	2.302300	-1.003101	1.590333
C	0.946724	-2.225931	0.409519
C	0.675990	-1.345675	-0.702736
C	-0.488208	-1.284371	2.062521
C	0.010375	-2.399225	1.478345
O	2.047593	0.958249	2.681403
Se	0.908463	-2.129742	-2.383393
H	1.678525	-3.004109	0.235337
H	-0.162847	-3.390416	1.875153
H	-1.166694	-1.353135	2.905439
H	-0.361406	0.848614	2.211930

INT:

$E_{B3LYP-D3}$= -5019.2788201
E_{M06-2X}= -5019.0915424
$E_{DF-CCSD(T)}$= -5015.394515555707

C	0.010340	-0.037417	0.000793
C	0.032665	-0.019293	1.506867
O	1.437302	0.011846	1.948252
C	2.219857	-0.970916	1.447849
C	1.532343	-1.835415	0.434738
C	0.780671	-0.985066	-0.530273
C	-0.675120	-1.269376	2.060348
Se	-2.240505	-1.013602	3.005415
O	3.371190	-1.084761	1.809887
As	0.149822	-2.875731	1.645921
H	-0.399130	0.878440	1.935668
H	-0.621775	0.640637	-0.551814
H	0.861948	-1.170411	-1.593579
H	2.236866	-2.531042	-0.004456

TS2:

$E_{B3LYP-D3}$= -5019.2503889
E_{M06-2X}= -5019.046615
$E_{DF-CCSD(T)}$= -5015.354000750539

As	0.077879	0.361038	-0.321679
C	0.057437	0.003222	1.679166
C	1.670732	-0.009730	2.024020
O	2.302300	-1.003101	1.590333
C	0.946724	-2.225931	0.409519
C	0.675990	-1.345675	-0.702736
C	-0.488208	-1.284371	2.062521
C	0.010375	-2.399225	1.478345
O	2.047593	0.958249	2.681403
Se	0.908463	-2.129742	-2.383393
H	1.678525	-3.004109	0.235337
H	-0.162847	-3.390416	1.875153
H	-1.166694	-1.353135	2.905439
H	-0.361406	0.848614	2.211930

PC:

$E_{B3LYP-D3}$= -5019.3248428
E_{M06-2X}= -5019.125211
$E_{DF-CCSD(T)}$= -5015.425405287922

C	0.000000	0.000000	0.000000
As	0.000000	0.000000	1.877383
C	1.880473	0.000000	2.177038
Se	2.538776	0.000943	3.939155
C	1.128730	-0.001143	-0.793756
C	2.431281	-0.002543	-0.268126
C	2.764398	-0.001737	1.075923
C	0.135575	-3.224453	0.027754
O	1.253137	-3.327287	0.315165
O	-0.985600	-3.183831	-0.280353
H	3.822831	-0.006619	1.320270
H	3.253460	-0.008519	-0.976333
H	1.020378	-0.000735	-1.875109
H	-0.971544	-0.005069	-0.484635

MeCP:

MeCP:
$E_{B3LYP-D3}$= -419.38672
E_{M06-2X}= -419.3067308
$E_{DF-CCSD(T)}$= -418.741620263767

C	0.000000	-0.000000	0.068863
P	0.000000	-0.000000	1.614210
C	0.000000	-0.000000	-1.386804
H	-1.020190	0.000000	-1.778025
H	0.510095	0.883510	-1.778025
H	0.510095	-0.883510	-1.778025

Product:
$E_{B3LYP-D3}$= -574.3197025
E_{M06-2X}= -574.1655263
$E_{DF-CCSD(T)}$= -573.329376901543

C	0.007754	-0.179960	-0.003798
P	0.014108	-0.252989	1.735388
C	1.716231	-0.035251	2.080909
C	2.157534	-0.036386	3.525913
C	1.124548	0.006496	-0.801248
C	2.405897	0.159077	-0.277332
C	2.674834	0.137720	1.086149
H	3.708187	0.265393	1.396009
H	3.231867	0.301035	-0.962023
H	1.002790	0.035793	-1.877908
H	-0.956145	-0.291293	-0.486593
H	2.880047	-0.834401	3.710556
H	2.642295	0.907595	3.784501
H	1.320408	-0.179622	4.208407

RP(A):

RC:
$E_{B3LYP-D3}$= -762.9012891
E_{M06-2X}= -762.677468
$E_{DF-CCSD(T)}$= -761.588207652161

C	-0.238275	-0.264337	-0.185994
H	-0.044374	0.234878	0.754220
C	0.844037	-0.847163	-0.909369
H	1.858245	-0.814662	-0.545142
C	0.566388	-1.457252	-2.076445
H	1.296089	-1.937731	-2.711281
O	-0.673264	-1.533072	-2.584621
C	-1.793778	-1.012907	-1.908308
O	-2.869217	-1.169984	-2.426232
C	-1.502034	-0.342527	-0.662859
H	-2.349955	0.073833	-0.141623
C	-1.627717	-4.423964	-1.288163
C	-2.408359	-4.514299	-2.510701
H	-3.274218	-5.165934	-2.370858
H	-2.766905	-3.526759	-2.811299
H	-1.806488	-4.921853	-3.326605
P	-0.799646	-4.313529	0.013474

TS1:
$E_{B3LYP-D3}$= -762.8619279
E_{M06-2X}= -762.6421304
$E_{DF-CCSD(T)}$= -761.555056571244

C	0.046695	0.224842	-0.228333
C	-0.062769	-0.324920	1.122972
O	1.130098	-0.324120	1.826451
C	2.270506	0.085596	1.181773
C	2.196801	1.103196	0.225541
C	1.021848	1.200602	-0.481822
O	-1.051637	-0.764497	1.646312
C	0.977815	-1.651372	-0.906043
C	-0.139733	-2.200008	-1.700213
P	2.453170	-1.861159	-0.325339
H	-0.880320	0.218634	-0.780913
H	0.913604	1.881242	-1.314036
H	3.087327	1.655759	-0.033596
H	3.137340	-0.058745	1.808735
H	0.109781	-3.178648	-2.110789
H	-1.029949	-2.307295	-1.075421
H	-0.401243	-1.533969	-2.525986

INT:
$E_{B3LYP-D3}$= =-762.9091436
E_{M06-2X}= -762.6988748
$E_{DF-CCSD(T)}$= -761.613810362714

C	0.108108	-0.052291	-0.306134
C	-0.032433	-0.216809	1.218564
O	1.163241	-0.318239	1.837520
C	2.320145	-0.226937	0.945350
C	2.199547	1.020131	0.118717
C	1.030589	1.134812	-0.500162
O	-1.070297	-0.250741	1.817011
C	0.748204	-1.355561	-0.799246
C	-0.071607	-2.188080	-1.730079
P	2.269101	-1.767993	-0.212930
H	-0.881312	0.096580	-0.726168
H	0.713414	1.982751	-1.087589
H	3.004492	1.738004	0.070623
H	3.182386	-0.250759	1.601155
H	0.435153	-3.105225	-2.025614
H	-1.022278	-2.456208	-1.259953
H	-0.317121	-1.618297	-2.631774

TS2:
$E_{B3LYP-D3}$= -762.8817817
E_{M06-2X}= -762.659373
$E_{DF-CCSD(T)}$= -761.575299466707

C	0.051605	0.135821	0.167168
P	0.278213	-0.089539	1.960570
C	1.980129	0.030543	1.896870
C	2.843653	-0.236847	3.091199
C	0.753809	1.179360	-0.521699
C	2.084869	1.227197	-0.305668
C	2.655585	0.250911	0.614910
O	1.073509	-1.401784	-0.487647
C	2.292816	-1.274441	-0.234334
O	3.277114	-1.938883	-0.433001
H	3.737439	0.255653	0.675848
H	2.745507	1.842027	-0.899843
H	0.261474	1.754601	-1.292622
H	-0.909332	-0.165963	-0.230194
H	3.498163	-1.090737	2.895403
H	3.491692	0.620214	3.292646
H	2.259826	-0.451446	3.984914

PC:
$E_{B3LYP-D3}$= -762.988329
E_{M06-2X}= -762.7646715
$E_{DF-CCSD(T)}$= -761.675201104840

C	0.057664	-0.609075	0.076162
C	0.149871	0.048240	1.292777
C	1.296816	0.011632	2.081216
C	2.445473	-0.681913	1.717360
C	2.591570	-1.412465	0.542348
C	3.909265	-2.098532	0.269859
P	1.319033	-1.562508	-0.650929
C	1.453631	2.303194	-0.862656
O	2.554210	2.011961	-0.640656
O	0.357290	2.609648	-1.090270
H	3.290534	-0.644946	2.398601
H	1.297084	0.549223	3.020421
H	-0.701693	0.616978	1.647629
H	-0.868646	-0.529352	-0.480325
H	4.711154	-1.363560	0.170810
H	4.178019	-2.766771	1.090618
H	3.882028	-2.686838	-0.646730

RP(B):

RC:
$E_{B3LYP-D3}$= -762.9012878
E_{M06-2X}= -762.6774709
$E_{DF-CCSD(T)}$= -761.588207652161

C	-0.238275	-0.264337	-0.185994
H	-0.044374	0.234878	0.754220
C	0.844037	-0.847163	-0.909369
H	1.858245	-0.814662	-0.545142
C	0.566388	-1.457252	-2.076445
H	1.296089	-1.937731	-2.711281
O	-0.673264	-1.533072	-2.584621
C	-1.793778	-1.012907	-1.908308
O	-2.869217	-1.169984	-2.426232
C	-1.502034	-0.342527	-0.662859
H	-2.349955	0.073833	-0.141623
C	-1.627717	-4.423964	-1.288163
C	-2.408359	-4.514299	-2.510701
H	-3.274218	-5.165934	-2.370858
H	-2.766905	-3.526759	-2.811299
H	-1.806488	-4.921853	-3.326605
P	-0.799646	-4.313529	0.013474

TS1:

$E_{B3LYP-D3}$= -762.8628068
E_{M06-2X}= -762.642566
$E_{DF-CCSD(T)}$= -761.555104198674

C	-0.091497	-0.037641	0.114564
C	0.057612	0.103846	1.496898
O	1.287490	-0.081753	2.069708
C	2.129504	-1.045149	1.536530
C	1.623594	-1.668683	0.314978
C	0.731535	-0.960945	-0.493562
O	3.170979	-1.278522	2.089752
C	-0.917261	-1.696700	2.192710
C	-2.087955	-1.143611	2.905313
P	-0.058420	-2.979566	1.792926
H	-0.516634	0.823505	2.061703
H	-0.928281	0.423250	-0.387397
H	0.595287	-1.242346	-1.528424
H	2.253503	-2.447925	-0.085675
H	-2.718409	-1.930673	3.319018
H	-1.766402	-0.500109	3.728707
H	-2.697446	-0.528725	2.237551

INT:

$E_{B3LYP-D3}$= =-762.91314
E_{M06-2X}= 762.7033261
$E_{DF-CCSD(T)}$= -761.618193273004

C	-0.096648	-0.046840	0.041606
C	-0.122602	-0.048970	1.550928
O	1.261453	-0.015473	2.033868
C	2.033481	-1.023424	1.562496
C	1.303493	-1.913749	0.561849
C	0.658928	-1.017548	-0.459343
O	3.169064	-1.169968	1.921387
C	-0.779027	-1.332713	2.067696
C	-1.995650	-1.182834	2.919717
P	-0.043323	-2.781743	1.646111
H	-0.576632	0.840636	1.977355
H	-0.633373	0.694605	-0.529018
H	0.838012	-1.178250	-1.512273
H	1.995159	-2.642459	0.157470
H	-2.409712	-2.140808	3.229525
H	-1.762129	-0.599851	3.815743
H	-2.773297	-0.632358	2.380184

TS2:
$E_{B3LYP\text{-}D3}$= -762.886563
$E_{M06\text{-}2X}$= -762.6646897
$E_{DF\text{-}CCSD(T)}$= -761.580478530763

C	0.195422	0.105134	0.156643
P	0.360472	0.094756	1.992832
C	2.057634	0.124790	2.028505
C	2.860388	-0.018389	3.289927
C	1.047608	1.021125	-0.583860
C	2.364941	1.016873	-0.289752
C	2.766858	0.131992	0.766290
C	0.842153	-1.465720	-0.216632
O	0.007985	-2.231676	-0.636714
O	2.076966	-1.560515	-0.005776
H	3.805808	-0.175257	0.790600
H	3.111031	1.508261	-0.895744
H	0.648325	1.561688	-1.431149
H	-0.837161	0.064097	-0.165003
H	3.569905	0.804436	3.403419
H	2.223316	-0.040351	4.173025
H	3.439211	-0.946203	3.265374

PC:
$E_{B3LYP\text{-}D3}$= -762.9883281
$E_{M06\text{-}2X}$= -762.7646738
$E_{DF\text{-}CCSD(T)}$= -761.675946917412

C	-0.138368	0.623006	0.330794
P	0.301209	-0.041084	1.879121
C	2.039138	0.169061	1.861019
C	2.829710	-0.301757	3.058456
C	0.745436	1.152803	-0.593249
C	2.119326	1.211058	-0.372097
C	2.720897	0.744187	0.790487
O	1.734637	-2.272764	-0.997245
C	2.519056	-2.480591	-0.169336
O	3.310887	-2.703218	0.650767
H	3.800663	0.832770	0.868686
H	2.749821	1.637783	-1.141163
H	0.359399	1.535793	-1.530633
H	-1.193558	0.610549	0.084240
H	3.532494	-1.085043	2.768504
H	3.408152	0.519001	3.487713
H	2.185750	-0.702690	3.840532

PCM geometries and their exact energies:

2*H*-pyran-2-one:

$E_{B3LYP-D3}$= -343.5147396
E_{M06-2X}= -343.3711346

O	0.008299	-0.000000	-0.003532
C	0.000064	-0.000000	1.344020
C	1.128912	0.000000	2.076524
C	2.375207	0.000000	1.384830
C	2.409483	0.000000	0.029654
C	1.195580	-0.000000	-0.745329
O	1.088335	-0.000000	-1.951816
H	3.333875	0.000000	-0.526747
H	3.298054	0.000000	1.948875
H	1.073661	0.000000	3.153095
H	-1.002757	-0.000000	1.743465

CO_2:

$E_{B3LYP-D3}$= -188.6656073
E_{M06-2X}= -188.5961911

C	0.000523	0.000000	0.000000
O	-0.000196	0.000000	1.160432
O	0.001242	0.000000	-1.160432

[PCO]$^-$:

[PCO]$^-$:
$E_{B3LYP-D3}$= -454.8789511
E_{M06-2X}= -454.7986836

C	0.000000	0.000000	-0.510585
O	0.000000	0.000000	-1.706277
P	0.000000	0.000000	1.114248

Product:
$E_{B3LYP-D3}$= -609.7630099
E_{M06-2X}= -609.6108827

C	-0.001848	0.000000	0.000599
C	0.002142	0.000000	1.382003
C	1.200601	0.000000	2.117683
C	2.463068	0.000000	1.564603
C	2.779324	-0.000000	0.162652
O	3.978570	-0.000000	-0.245124
P	1.409113	-0.000000	-1.039053
H	3.323348	0.000000	2.228485
H	1.128222	0.000000	3.200131
H	-0.937319	0.000000	1.922649
H	-0.962189	-0.000000	-0.506631

RP(A):

RC:
$E_{B3LYP-D3}$= -798.3998055
E_{M06-2X}= -798.1752878

C	0.431101	1.017134	0.000530
C	1.705433	1.651544	0.000402
C	2.844268	0.913609	0.000061
C	2.795892	-0.524095	-0.000180
O	1.517096	-1.083607	-0.000060
C	0.396331	-0.329600	0.000281
O	3.734543	-1.294376	-0.000455
O	-2.906011	1.588404	-0.000770
C	-3.185208	0.424583	-0.000325
P	-3.551942	-1.156215	0.000268
H	3.826194	1.361054	-0.000045
H	1.762983	2.731927	0.000579
H	-0.494300	1.570342	0.000784
H	-0.507680	-0.922517	0.000328

TS1:

$E_{B3LYP-D3}$= -798.3622266

E_{M06-2X}= -798.1439814

C	0.151862	-0.127053	-0.113099
O	0.135295	-0.172582	1.268376
C	1.408128	0.035599	1.899935
C	2.053186	1.277707	1.444323
C	2.008783	1.470873	0.107572
C	1.285101	0.524944	-0.681437
P	2.560375	-1.552328	1.540602
C	2.530968	-1.535642	-0.165767
O	2.785452	-1.991793	-1.226831
O	-0.807350	-0.627366	-0.702029
H	1.320727	0.558492	-1.759877
H	2.543938	2.278505	-0.376356
H	2.618236	1.887702	2.132909
H	1.226801	-0.015970	2.968683

INT:

$E_{B3LYP-D3}$= -798.3816138

E_{M06-2X}= -798.1721953

O	0.071354	0.128969	-0.025241
C	0.178834	-0.530420	1.307284
C	1.232027	0.208421	2.084656
C	2.389375	0.339853	1.443995
C	2.417662	-0.340109	0.096381
C	1.233721	0.193391	-0.689554
O	1.287722	0.664850	-1.806862
H	3.330590	-0.182384	-0.467120
H	3.262535	0.847596	1.824676
H	1.029230	0.572516	3.081625
H	-0.816455	-0.445915	1.728027
C	2.197907	-1.907606	0.266790
P	0.672357	-2.362110	1.083992
O	3.067510	-2.649931	-0.206984

TS2:
$E_{B3LYP-D3}$= -798.3667878
E_{M06-2X}= -798.1466968

C	0.121914	-0.107890	-0.023961
C	0.106609	-0.095208	1.470365
C	1.617765	-0.044942	1.943883
O	2.407386	-0.893995	1.403384
C	1.300434	-2.157984	0.330638
C	0.758127	-1.139780	-0.578847
C	-0.531338	-1.381907	2.051688
O	-1.431627	-1.285603	2.884426
O	1.935258	0.807953	2.776266
P	0.265375	-2.946692	1.549149
H	-0.406259	0.761264	1.895086
H	-0.289730	0.718560	-0.584388
H	0.922220	-1.242637	-1.643297
H	2.106142	-2.764880	-0.065548

PC:
$E_{B3LYP-D3}$= -798.434969
E_{M06-2X}= -798.212671

C	2.855221	-0.304004	0.370012
P	1.539342	-1.450471	0.218004
C	0.173678	-0.399949	-0.364760
O	-0.964156	-0.915428	-0.590924
C	2.791886	1.049754	0.100495
C	1.606721	1.660468	-0.344868
C	0.413844	1.002208	-0.557643
C	-3.197244	0.246105	0.292280
O	-2.629052	1.028306	0.936189
O	-3.859143	-0.491158	-0.314066
H	-0.443258	1.574855	-0.899010
H	1.630659	2.728246	-0.535544
H	3.674270	1.665682	0.232276
H	3.802394	-0.712964	0.709532

RP(B):

RC:

$E_{B3LYP-D3}$= -798.4001327
E_{M06-2X}= -798.1756858

O	2.495793	-1.166158	-0.009534
C	2.980810	0.143686	-0.008953
C	1.979281	1.176137	0.014140
C	0.657255	0.871744	0.033199
C	0.229292	-0.488162	0.030661
C	1.173751	-1.445295	0.009289
O	4.186968	0.281708	-0.027870
O	-2.556788	1.664906	-0.016164
C	-3.140674	0.620538	-0.011559
P	-3.913485	-0.806613	-0.005629
H	0.986273	-2.508311	0.004716
H	-0.817785	-0.751932	0.043705
H	-0.092168	1.650607	0.049227
H	2.339871	2.193290	0.014667

TS1:

$E_{B3LYP-D3}$=-798.3480255
E_{M06-2X}= -798.1279616

C	-0.122914	0.266274	0.113069
O	-0.009276	-0.024402	1.477109
C	1.236050	-0.048599	2.022458
C	2.317191	0.175026	1.041394
C	2.033042	1.214879	0.090476
C	0.764494	1.173792	-0.449698
O	1.365251	-0.237956	3.215900
P	2.315478	-1.799034	0.033662
C	0.808554	-1.792799	-0.731704
O	-0.062337	-2.236031	-1.417558
H	-1.129591	0.094406	-0.230837
H	0.497389	1.691019	-1.362267
H	2.832989	1.795236	-0.346236
H	3.291145	0.161232	1.511671

INT:
$E_{B3LYP-D3}$= -798.3861538
E_{M06-2X}= -798.1775288

O	0.104034	0.090326	-0.072058
C	0.033422	-0.054231	1.270348
C	1.364181	0.027211	1.959805
C	2.107692	1.223250	1.441525
C	2.172456	1.325250	0.115581
C	1.474247	0.201549	-0.590921
P	2.228362	-1.639669	1.402827
C	2.217066	-1.155669	-0.316565
O	2.710642	-1.746171	-1.291170
H	1.361368	0.337572	-1.661263
H	2.679674	2.105701	-0.430788
H	2.551159	1.928936	2.130398
H	1.223148	0.017139	3.033994
O	-1.044297	-0.229553	1.804223

TS2:
$E_{B3LYP-D3}$=-798.3540261
E_{M06-2X}= -798.1293325

C	0.068354	-0.132219	-0.048073
C	-0.239680	-0.140601	1.332896
O	1.801618	-0.046433	2.008136
C	2.401621	-1.022510	1.495938
C	1.497714	-1.987844	0.503394
C	0.917338	-1.098663	-0.485637
C	-0.632951	-1.374225	2.069326
O	-1.609842	-1.279976	2.854704
O	3.577030	-1.374904	1.618815
P	0.347894	-2.817924	1.758702
H	-0.578796	0.775178	1.801886
H	-0.219231	0.709431	-0.662922
H	1.284162	-1.109159	-1.504085
H	2.184047	-2.715758	0.087772

PC:
$E_{B3LYP-D3}$= -798.4342786
E_{M06-2X}= -798.2114875

C	0.182446	0.558068	-1.384544
P	-0.651480	-0.955343	-1.090182
C	-1.749515	-0.529181	0.300369
O	-2.515501	-1.409768	0.791154
C	0.002313	1.738111	-0.689560
C	-0.922155	1.841561	0.365765
C	-1.721468	0.813225	0.815117
C	2.582370	-0.357803	0.640226
O	3.173987	-0.535043	-0.342958
O	2.022617	-0.182513	1.640622
H	-2.402424	1.002940	1.640439
H	-1.011935	2.800867	0.864479
H	0.581782	2.614110	-0.958290
H	0.911999	0.546939	-2.188674

$[PCS]^{-}$:

$[PCS]^{-}$:
$E_{B3LYP-D3}$= -777.8426799
E_{M06-2X}= -777.7562067

C	0.000000	0.000000	0.220963
P	0.000000	0.000000	1.819500
S	0.000000	0.000000	-1.410463

Product:
$E_{B3LYP-D3}$= -932.7451543
E_{M06-2X}= -932.5877879

P	-0.390031	0.000000	-0.209374
C	-0.099025	0.000000	1.512280
C	1.145924	0.000000	2.117968
C	2.328040	-0.000000	1.370381
C	2.374144	-0.000000	-0.015520
C	1.255850	-0.000000	-0.876148
S	1.452473	-0.000000	-2.613938
H	3.352196	-0.000000	-0.485650
H	3.271150	-0.000000	1.904076
H	1.215185	0.000000	3.199693
H	-0.980326	0.000000	2.145768

RP(A):

RC:

$E_{B3LYP-D3}$= -1121.3640962
E_{M06-2X}= -1121.133598

C	0.000000	0.000000	0.000000
C	0.000000	0.000000	1.424795
C	1.168173	0.000000	2.114715
C	2.434287	0.000043	1.431529
O	2.365731	0.000084	0.036281
C	1.189243	0.000083	-0.628097
O	3.543093	0.000031	1.926082
C	-3.554552	0.000124	-0.953836
P	-3.330537	-0.000409	-2.533381
S	-3.763750	0.000809	0.664864
H	1.204951	-0.000048	3.193253
H	-0.948961	-0.000081	1.943346
H	-0.918099	-0.000048	-0.566056
H	1.330135	0.000100	-1.698277

TS1:

$E_{B3LYP-D3}$= -1121.3207292
E_{M06-2X}= -1121.0935989

C	0.095834	-0.145734	-0.110581
O	0.100469	-0.153935	1.268349
C	1.373108	0.001818	1.887413
C	2.139409	1.122956	1.370685
C	2.105031	1.264688	0.018805
C	1.282203	0.380478	-0.717056
P	2.422306	-1.726455	1.520950
C	2.304503	-1.833392	-0.133023
S	2.501931	-2.601832	-1.548357
O	-0.915353	-0.546995	-0.676488
H	1.319976	0.341473	-1.793948
H	2.746302	1.964731	-0.500667
H	2.810487	1.668043	2.017111
H	1.211570	-0.019579	2.958729

INT:

$E_{B3LYP\text{-}D3}$= -1121.3539821

$E_{M06\text{-}2X}$= -1121.1395126

O	0.072514	0.006089	-0.055637
C	0.176168	-0.607051	1.287956
C	1.176520	0.182734	2.080398
C	2.344574	0.341174	1.466442
C	2.437910	-0.367524	0.129468
C	1.249748	0.132872	-0.687058
O	1.316162	0.625344	-1.791388
H	3.351108	-0.151807	-0.411978
H	3.186462	0.898144	1.848363
H	0.933543	0.557710	3.063975
H	-0.832256	-0.574827	1.683614
C	2.255709	-1.906110	0.293864
P	0.799533	-2.410950	1.083754
S	3.472440	-2.917952	-0.340866

TS2:

$E_{B3LYP\text{-}D3}$= -1121.3346792

$E_{M06\text{-}2X}$= -1121.1076023

C	0.095834	-0.145734	-0.110581
O	0.100469	-0.153935	1.268349
C	1.373108	0.001818	1.887413
C	2.139409	1.122956	1.370685
C	2.105031	1.264688	0.018805
C	1.282203	0.380478	-0.717056
P	2.422306	-1.726455	1.520950
C	2.304503	-1.833392	-0.133023
S	2.501931	-2.601832	-1.548357
O	-0.915353	-0.546995	-0.676488
H	1.319976	0.341473	-1.793948
H	2.746302	1.964731	-0.500667
H	2.810487	1.668043	2.017111
H	1.211570	-0.019579	2.958729

PC:
$E_{B3LYP-D3}$= -1121.4150076
E_{M06-2X}= -1121.188795

C	0.000000	0.000000	0.000000
P	0.000000	0.000000	1.745295
C	1.734030	0.000000	2.126800
S	2.221175	-0.017483	3.807004
C	1.126387	-0.003421	-0.805645
C	2.416573	0.000380	-0.266763
C	2.693332	0.005499	1.092390
O	4.138943	-2.847317	2.133279
C	3.023098	-3.140271	2.261252
O	1.916528	-3.469240	2.374003
H	3.735408	0.006041	1.393338
H	3.257371	0.000159	-0.950370
H	1.013773	-0.006582	-1.883727
H	-0.974599	0.000725	-0.477554

RP(B):

RC:
$E_{B3LYP-D3}$= -1121.3637175
E_{M06-2X}= -1121.134146

C	0.000000	0.000000	0.000000
O	0.000000	0.000000	1.349149
C	1.179036	0.000000	2.098572
C	2.397153	0.000204	1.335603
C	2.372428	-0.000289	-0.022500
C	1.135585	-0.000828	-0.723156
O	1.061428	-0.000227	3.307588
C	-1.573706	0.000714	-3.155125
P	-1.640029	-1.589467	-3.255433
S	-1.493923	1.625272	-3.030634
H	-0.994051	0.000850	-0.418902
H	1.072854	0.000178	-1.799012
H	3.300720	0.000220	-0.578181
H	3.317567	0.000982	1.898661

TS1:
$E_{B3LYP-D3}$=-1121.3155555
E_{M06-2X}= -1121.0908277

C	-0.034627	0.180779	0.087966
C	0.043946	-0.019157	1.483848
C	1.298710	0.002265	2.044878
C	2.356906	0.261846	1.169724
O	2.139995	1.129355	0.109791
C	0.925979	1.091074	-0.516430
C	2.542986	-1.658323	0.062104
S	4.023448	-2.279861	0.502051
O	0.750010	1.804024	-1.488608
P	1.160700	-1.748250	-0.826885
H	3.387463	0.294692	1.481205
H	1.502060	-0.341143	3.047773
H	-0.824331	-0.351214	2.034753
H	-0.984251	0.129002	-0.422794

INT:
$E_{B3LYP-D3}$= -1121.3606234
E_{M06-2X}= -1121.1466764

C	0.006905	-0.022938	-0.015266
C	0.010641	-0.022742	1.490962
O	1.414288	-0.006385	1.948151
C	2.165717	-1.012683	1.458203
C	1.441650	-1.875586	0.462115
C	0.757418	-0.988814	-0.536748
C	-0.668691	-1.301150	2.025284
S	-2.091673	-1.121002	2.948410
O	3.312642	-1.158524	1.829416
P	0.116981	-2.777083	1.587735
H	-0.427030	0.863905	1.933739
H	-0.570649	0.699172	-0.571057
H	0.888556	-1.161652	-1.595596
H	2.122078	-2.602266	0.035078

TS2:

$E_{B3LYP\text{-}D3}$= -1121.3339207
$E_{M06\text{-}2X}$= -1121.1050666

C	0.019379	0.009117	-0.053911
C	0.016371	0.065439	1.401995
C	1.208444	-0.007557	2.038496
C	2.352968	-0.242146	1.220349
O	2.016760	1.449410	-0.074128
C	0.875302	1.312994	-0.577487
C	2.360724	-1.296408	0.215195
S	3.740830	-2.331220	0.223442
O	0.293489	2.011173	-1.410112
P	0.994661	-1.413191	-0.832647
H	3.320323	0.096277	1.567322
H	1.335058	0.248859	3.080581
H	-0.895367	0.312287	1.930249
H	-0.963801	0.087770	-0.502208

PC:

$E_{B3LYP\text{-}D3}$= -1121.4150076
$E_{M06\text{-}2X}$= -1121.183372

C	0.000000	0.000000	0.000000
C	0.000000	0.000000	1.391622
C	1.196089	0.000000	2.130601
C	2.467609	0.001228	1.559932
C	2.749547	0.004797	0.170313
S	4.407785	0.013032	-0.409376
P	1.425294	0.002413	-1.022445
O	-1.828243	3.421156	-1.249049
C	-0.949344	4.094490	-0.872688
O	-0.071015	4.769589	-0.499576
H	3.324741	0.001714	2.233246
H	1.130371	-0.000686	3.217544
H	-0.946632	-0.000231	1.930564
H	-0.962172	0.000648	-0.514116

$[PCSe]^{-}$:

$[PCSe]^{-}$:
$E_{B3LYP-D3}$= -2781.2462168
E_{M06-2X}= -2781.1838383

C	-0.239873	-0.000000	-0.032005
P	0.059814	-0.000000	1.528133
Se	-0.577807	-0.000000	-1.789699

Product:
$E_{B3LYP-D3}$= -2936.1551764
E_{M06-2X}= -2936.021813

C	-0.045432	0.044211	0.019511
C	0.340134	-0.514618	1.231262
C	1.425387	-0.084892	2.013165
P	2.443060	1.264736	1.504023
C	1.710187	1.728190	-0.011021
C	0.605498	1.121607	-0.586086
Se	1.841201	-0.952921	3.667190
H	-0.242421	-1.350334	1.604297
H	-0.903838	-0.385309	-0.482900
H	0.228708	1.488055	-1.534196
H	2.170435	2.561181	-0.532523

RP(A):

RC:
$E_{B3LYP-D3}$= -3124.7678429
E_{M06-2X}= -3124.561478

C	0.000000	0.000000	0.000000
O	0.000000	0.000000	1.350993
C	1.179385	0.000000	2.097607
C	2.398046	-0.000023	1.332964
C	2.372279	0.000018	-0.023802
C	1.133400	-0.000022	-0.725726
O	1.065514	0.000057	3.306515
C	0.019289	-0.001289	-4.258587
P	1.482174	-0.001801	-4.877798
Se	-1.619648	-0.000675	-3.542655
H	-0.995705	-0.000104	-0.416206
H	1.080824	0.000137	-1.803023
H	3.299012	0.000001	-0.581983
H	3.318254	-0.000074	1.896492

TS1:
$E_{B3LYP\text{-}D3}$= -3124.7240552
$E_{M06\text{-}2X}$= -3124.5197486

C	-0.032231	0.000846	0.037645
C	0.070295	0.090341	1.472058
O	1.361271	-0.168182	1.989219
C	2.006488	-1.286111	1.504428
C	1.439262	-1.843774	0.309932
C	0.655776	-1.031295	-0.531407
P	-1.163824	-1.420490	2.280390
C	-0.334385	-2.776389	1.848577
Se	-0.036510	-4.535062	1.880849
O	3.006497	-1.666215	2.098889
H	-0.304608	0.987535	1.948107
H	-0.757961	0.599353	-0.491818
H	0.532276	-1.292575	-1.573951
H	1.891304	-2.755696	-0.045124

INT:
$E_{B3LYP\text{-}D3}$= -3124.7619296
$E_{M06\text{-}2X}$= -3124.5709761

C	-0.125387	-0.121852	-0.033970
C	0.049089	-0.025951	1.504289
C	1.551822	-0.055044	1.776868
O	2.136480	-1.214919	1.438148
C	1.205092	-2.213666	0.869595
P	0.529160	-1.518432	-0.791040
C	-0.511441	-1.253731	2.196596
C	0.070066	-2.392493	1.834323
O	2.192219	0.847228	2.267559
Se	-0.992228	1.288759	-0.896934
H	1.809605	-3.100359	0.719562
H	-0.223294	-3.376779	2.168857
H	-1.321530	-1.157606	2.903218
H	-0.356243	0.904141	1.883792

TS2:
$E_{B3LYP-D3}$= -3124.7412733
E_{M06-2X}= -3124.5368106

C	0.241206	0.062853	0.015002
C	-0.084752	0.123626	1.428237
O	1.849579	0.004941	2.093549
C	2.346191	-1.072290	1.644712
C	1.382847	-1.948228	0.669155
C	0.957286	-0.992296	-0.391054
P	-0.803837	-1.287547	2.268495
C	0.250344	-2.469039	1.522385
Se	0.129681	-4.278089	1.876118
O	3.474757	-1.520294	1.840646
H	-0.325939	1.100022	1.831605
H	-0.026836	0.886788	-0.632255
H	1.330745	-1.106845	-1.398175
H	1.996155	-2.757605	0.291958

PC:
$E_{B3LYP-D3}$= -3124.8251563
E_{M06-2X}= -3124.62281

C	0.000000	0.000000	0.000000
P	0.000000	0.000000	1.745269
C	1.726336	0.000000	2.116467
Se	2.259293	0.022750	3.953527
C	1.129270	0.004671	-0.803072
C	2.418331	0.002290	-0.266009
C	2.691486	-0.002966	1.095955
O	4.007846	2.927311	2.009005
C	2.887623	3.182531	2.173792
O	1.774422	3.473298	2.321063
H	-0.973044	-0.000406	-0.480373
H	1.017227	0.007963	-1.881297
H	3.259754	0.004719	-0.948462
H	3.732998	0.000281	1.397741

RP(B):

RC:
$E_{B3LYP-D3}$= -3124.7674465
E_{M06-2X}= -3124.5617226

C	0.000000	0.000000	0.000000
C	0.000000	0.000000	1.358289
C	1.224179	0.000000	2.081113
C	2.372819	-0.002220	1.378715
O	2.397448	-0.002566	0.029850
C	1.232054	-0.000089	-0.740722
O	1.371362	0.002135	-1.947253
C	3.977932	-0.571479	4.505180
Se	3.884205	1.199949	4.732147
P	4.050903	-2.141574	4.280903
H	3.359296	-0.006450	1.815391
H	1.266874	0.005247	3.157985
H	-0.938172	0.000657	1.897130
H	-0.909779	0.000984	-0.580241

TS1:
$E_{B3LYP-D3}$= -3124.7223535
E_{M06-2X}= -3124.521437

P	-0.013407	0.283995	-0.150171
C	0.017914	-0.043729	2.356048
C	1.465194	0.064639	2.320470
O	2.107510	-1.034952	1.815567
C	1.341202	-2.060494	1.291248
C	-0.552066	-1.323834	2.479450
C	0.130868	-2.376673	1.917590
O	2.140157	1.004395	2.701181
H	1.968640	-2.800459	0.823313
H	-0.302761	-3.355508	1.782788
H	-1.559860	-1.434229	2.853974
H	-0.494616	0.832125	2.723278
C	0.670296	-1.138167	-0.576049
Se	1.176299	-2.300672	-1.901377

INT:
$E_{B3LYP\text{-}D3}$= -3124.7687736
$E_{M06\text{-}2X}$= -3124.578303

C	0.020943	-0.018367	-0.024162
C	0.029996	-0.009126	1.482353
O	1.439983	0.033665	1.929791
C	2.203779	-0.962215	1.442118
C	1.486938	-1.845719	0.456549
C	0.784733	-0.974604	-0.544015
C	-0.616858	-1.294531	2.024916
Se	-2.168368	-1.124421	3.046113
O	3.355879	-1.087779	1.804163
P	0.176265	-2.752865	1.594762
H	-0.414106	0.876984	1.919457
H	-0.569829	0.692429	-0.580495
H	0.915224	-1.150163	-1.602308
H	2.175627	-2.565586	0.031019

TS2:
$E_{B3LYP\text{-}D3}$= -3124.7437658
$E_{M06\text{-}2X}$= -3124.5383

P	0.098976	0.262617	-0.246643
C	0.058903	-0.011058	1.626608
C	1.657069	-0.021215	1.992682
O	2.299831	-1.019605	1.583604
C	0.943010	-2.233195	0.434560
C	0.670384	-1.304894	-0.645026
C	-0.488781	-1.291867	2.064306
C	0.002093	-2.415824	1.496179
O	2.050376	0.957474	2.630541
Se	0.929206	-2.000193	-2.376970
H	1.668925	-3.011559	0.241913
H	-0.173367	-3.404246	1.895713
H	-1.152455	-1.329449	2.918217
H	-0.367307	0.848698	2.129673

PC:
$E_{B3LYP\text{-}D3}$= -3124.8252406
$E_{M06\text{-}2X}$= -3124.6228099

C	0.000000	0.000000	0.000000
C	0.000000	0.000000	1.385569
C	1.184468	0.000000	2.125506
C	2.452832	-0.003097	1.559284
C	2.725223	-0.006706	0.181456
Se	4.532164	-0.029919	-0.451878
P	1.423046	-0.003850	-1.010488
C	3.209439	-3.332270	0.368841
O	3.544445	-3.200229	1.471871
O	2.860144	-3.504347	-0.724082
H	-0.955112	0.001121	-0.515027
H	-0.943903	0.000774	1.918712
H	1.115069	-0.000899	3.206596
H	3.301857	-0.010363	2.233899

$[AsCO]^{-}$:

$[AsCO]^{-}$:
$E_{B3LYP\text{-}D3}$= -2349.4549444
$E_{M06\text{-}2X}$= -2349.38152

C	-0.000000	0.000000	-0.099442
O	-0.000000	0.000000	1.088984
As	-0.000000	0.000000	-1.853341
XX	1.000000	0.000000	-0.099442

Product:
$E_{B3LYP\text{-}D3}$= -2504.3369595
$E_{M06\text{-}2X}$= -2504.1887475

As	-0.478491	0.000000	-0.299514
C	-0.121703	0.000000	1.543876
C	1.129157	-0.000000	2.119088
C	2.322783	-0.000000	1.368571
C	2.426130	-0.000000	-0.005690
C	1.357254	-0.000000	-0.967358
O	1.584507	-0.000000	-2.206439
H	3.421574	-0.000000	-0.443148
H	3.253293	-0.000000	1.927067
H	1.216898	-0.000000	3.200872
H	-0.987635	0.000000	2.198738

RP(A):

RC:

$E_{B3LYP-D3}$= -2692.975753
E_{M06-2X}= -2692.757959

C	0.000000	0.000000	0.000000
C	0.000000	0.000000	1.358460
C	1.233696	0.000000	2.102761
O	2.396865	-0.000376	1.323735
C	2.370876	-0.000338	-0.027315
C	1.218508	-0.000113	-0.730062
O	1.365091	0.000269	3.306403
O	2.028315	-0.001582	-4.002096
C	3.169574	-0.001926	-3.652546
As	4.834587	-0.002399	-3.112321
H	-0.907338	0.000085	1.943289
H	-0.940051	0.000082	-0.538155
H	1.253816	-0.000158	-1.808057
H	3.354304	-0.000574	-0.480952

TS1:

$E_{B3LYP-D3}$= -2692.9404997
E_{M06-2X}= -2692.727667

C	0.194534	-0.189372	-0.122488
O	0.163183	-0.220456	1.259562
C	1.405836	0.026901	1.919109
C	2.069275	1.239623	1.444037
C	2.037878	1.422497	0.102842
C	1.320200	0.477598	-0.687748
As	2.667123	-1.684853	1.583617
C	2.597061	-1.594627	-0.259868
O	2.832427	-2.010991	-1.336327
O	-0.749348	-0.713075	-0.714756
H	1.356741	0.513207	-1.765767
H	2.592772	2.217052	-0.380068
H	2.651840	1.843103	2.124122
H	1.209918	-0.020112	2.984116

INT:
$E_{B3LYP-D3}$= -2692.9593578
E_{M06-2X}= -2692.75605

C	0.118006	-0.102298	-0.034922
O	0.076487	-0.131676	1.304761
C	1.389751	0.012538	1.969956
C	2.048279	1.246081	1.439059
C	2.069562	1.346973	0.112273
C	1.498858	0.151554	-0.608267
As	2.547831	-1.621263	1.532412
C	2.386985	-1.144796	-0.331469
O	2.844690	-1.727026	-1.313503
O	-0.894358	-0.245879	-0.689141
H	1.421941	0.275458	-1.683167
H	2.495337	2.169111	-0.442649
H	2.482851	1.967846	2.116213
H	1.159299	0.049329	3.027655

TS2:
$E_{B3LYP-D3}$= -2692.9404175
E_{M06-2X}= -2692.725386

C	0.119591	-0.122634	-0.024052
C	0.107346	-0.088841	1.467633
C	1.616110	-0.046190	1.960287
O	2.419369	-0.869211	1.409204
C	1.332881	-2.172192	0.283736
C	0.760607	-1.148799	-0.588397
C	-0.561286	-1.343784	2.082399
O	-1.453019	-1.209532	2.909202
O	1.907291	0.785350	2.825443
As	0.264191	-3.061644	1.582814
H	-0.393121	0.784635	1.873512
H	-0.303526	0.695535	-0.588324
H	0.908239	-1.233783	-1.657779
H	2.157603	-2.742652	-0.126833

PC:
$E_{B3LYP-D3}$= -2693.0069925
E_{M06-2X}= -2692.790124

C	0.000000	0.000000	0.000000
C	0.000000	0.000000	1.379890
C	1.182025	0.000000	2.145747
C	2.474081	0.016159	1.663359
C	2.905850	0.060903	0.286006
O	4.113755	0.126066	-0.022499
As	1.515080	0.022399	-1.104491
C	2.750996	3.124127	1.911270
O	2.069467	3.399721	1.015294
O	3.438022	2.915978	2.826249
H	3.290968	0.012273	2.381047
H	1.066549	-0.016757	3.226358
H	-0.946126	-0.007877	1.914482
H	-0.960586	-0.010417	-0.508412

RP(B):

RC:
$E_{B3LYP-D3}$= -2692.9760609
E_{M06-2X}= -2692.7591341

C	0.000000	0.000000	0.000000
C	0.000000	0.000000	1.426945
C	1.186178	0.000000	2.055657
O	2.373537	-0.002019	1.401607
C	2.444945	0.000027	0.000823
C	1.174712	0.000789	-0.679564
O	3.550190	0.001299	-0.494172
O	-2.849292	1.495595	0.605830
C	-3.252755	0.375263	0.655407
As	-3.760896	-1.297740	0.724115
H	1.322078	-0.001171	3.127201
H	-0.929810	-0.018360	1.972873
H	-0.951300	-0.013566	-0.512638
H	1.219039	0.000412	-1.758209

TS1:
$E_{B3LYP-D3}$= -2692.9306299
E_{M06-2X}= -2692.7159546

C	-0.074866	0.173845	0.081583
O	0.024608	-0.071222	1.455771
C	1.263223	-0.059380	2.021343
C	2.344435	0.292372	1.111085
C	2.038567	1.215835	0.078477
C	0.783763	1.120583	-0.479297
O	1.363465	-0.315207	3.211664
As	2.318397	-1.985286	0.044570
C	0.688884	-1.782332	-0.766023
O	-0.188682	-2.166792	-1.483166
H	-1.089135	0.019837	-0.250999
H	0.511957	1.614911	-1.401026
H	2.819802	1.817218	-0.363627
H	3.321940	0.292395	1.569322

INT:
$E_{B3LYP-D3}$= -2692.9667038
E_{M06-2X}= -2692.7640085

C	-0.002065	-0.040071	-0.026797
O	0.013940	-0.059374	1.436994
C	1.248917	-0.054269	1.995571
C	2.367302	0.095182	1.020171
C	2.043587	1.152529	0.019525
C	0.826090	1.105781	-0.520811
O	1.357140	-0.154108	3.203221
As	2.350840	-1.795880	0.101657
C	0.578793	-1.404270	-0.553626
O	-0.125882	-2.072770	-1.313224
H	-1.052198	0.017473	-0.295971
H	0.448124	1.787121	-1.267490
H	2.785582	1.897825	-0.233423
H	3.308405	0.224143	1.539404

TS2:
$E_{B3LYP-D3}$= -2692.9316825
E_{M06-2X}= -2692.7115402

C	0.065027	-0.139374	-0.043169
C	-0.259343	-0.138185	1.330783
O	1.815776	-0.062922	2.021480
C	2.416040	-1.034587	1.501178
C	1.551401	-1.971014	0.468720
C	0.922373	-1.096537	-0.492636
C	-0.679000	-1.344380	2.094920
O	-1.649572	-1.215811	2.872478
O	3.591128	-1.391212	1.641261
As	0.347495	-2.924743	1.803315
H	-0.591283	0.789771	1.782927
H	-0.228366	0.699050	-0.660445
H	1.252479	-1.105879	-1.524215
H	2.245123	-2.679788	0.034256

PC:
$E_{B3LYP-D3}$= -2693.0067755
E_{M06-2X}= -2692.7895348

C	0.000000	0.000000	0.000000
C	0.000000	0.000000	1.378112
C	1.184073	0.000000	2.146558
C	2.473418	-0.000212	1.667900
C	2.909686	0.002466	0.289445
O	4.117022	0.002634	-0.018210
As	1.520159	0.006951	-1.106256
C	0.000841	3.179933	-0.003293
O	0.899945	3.355824	0.706632
O	-0.923364	3.083212	-0.703334
H	3.290469	0.003724	2.386322
H	1.065013	0.003609	3.226745
H	-0.946454	-0.002825	1.912502
H	-0.959707	0.005547	-0.508975

$[AsCS]^-$:

$[AsCS]^-$:
$E_{B3LYP-D3}$= -2672.4153341
E_{M06-2X}= -2672.3337974

C	-0.004388	-0.000000	-0.106872
S	0.000504	-0.000000	1.517726
As	-0.014726	-0.000000	-1.824578

Product:
$E_{B3LYP-D3}$= -2827.3154046
E_{M06-2X}= -2827.1629845

C	-0.020817	0.040452	0.040976
C	0.350868	-0.527771	1.250240
C	1.421188	-0.138247	2.081748
As	2.532024	1.319900	1.568440
C	1.708454	1.768386	-0.049841
C	0.610687	1.124851	-0.583809
S	1.744994	-0.969951	3.577399
H	-0.244231	-1.366034	1.600683
H	-0.875895	-0.396215	-0.462192
H	0.207846	1.464498	-1.532440
H	2.137801	2.600038	-0.598481

RP(A):

RC:
$E_{B3LYP-D3}$= -3015.8963271
E_{M06-2X}= -3015.7117343

C	0.000000	0.000000	0.000000
C	0.000000	0.000000	1.360986
C	1.215708	0.000000	2.088630
C	2.367730	0.001438	1.386426
O	2.396202	0.002849	0.038424
C	1.231478	0.001865	-0.743679
O	1.368578	0.003050	-1.947179
C	3.728166	0.026174	4.565413
S	3.705035	1.635114	4.359829
As	3.730712	-1.683107	4.739074
H	3.349473	0.002097	1.834598
H	1.267187	-0.004981	3.165311
H	-0.941028	0.000016	1.897521
H	-0.907961	-0.000951	-0.583685

TS1:
$E_{B3LYP-D3}$= -3015.8963271
E_{M06-2X}= -3015.6740188

C	-0.046428	-0.022717	0.048505
C	0.057129	0.083971	1.482227
O	1.346731	-0.186636	2.000977
C	1.980793	-1.315227	1.527097
C	1.408491	-1.879529	0.338960
C	0.637533	-1.058408	-0.513584
As	-1.256095	-1.491764	2.344990
C	-0.297560	-2.903505	1.807142
S	0.032808	-4.491861	1.786584
O	2.972499	-1.703306	2.132401
H	-0.306734	0.988941	1.950944
H	-0.763905	0.580290	-0.487631
H	0.518961	-1.321596	-1.556240
H	1.866630	-2.787249	-0.018872

INT:
$E_{B3LYP-D3}$= -3015.9290238
E_{M06-2X}= -3015.7196359

C	-0.158496	-0.075641	-0.032072
C	0.056793	-0.028330	1.509951
C	1.560555	-0.058595	1.767101
O	2.146670	-1.223828	1.453525
C	1.236317	-2.249496	0.913955
As	0.537149	-1.573103	-0.898375
C	-0.507734	-1.256751	2.193286
C	0.079748	-2.398831	1.847319
O	2.205762	0.853575	2.235174
S	-0.957196	1.234249	-0.755213
H	1.848021	-3.136126	0.802375
H	-0.236862	-3.378688	2.175481
H	-1.339895	-1.164113	2.874358
H	-0.339898	0.899090	1.907066

TS2:
$E_{B3LYP-D3}$= -3015.9059579
E_{M06-2X}= -3015.6837281

C	0.229791	0.062827	0.016346
C	-0.089076	0.180478	1.425275
O	1.868640	0.011167	2.080153
C	2.335878	-1.089788	1.659670
C	1.373354	-1.955722	0.683627
C	0.928635	-1.008594	-0.377186
As	-0.895505	-1.270140	2.373014
C	0.256991	-2.528036	1.534837
S	0.193040	-4.179858	1.829039
O	3.447643	-1.568644	1.888440
H	-0.294034	1.177431	1.796991
H	-0.037982	0.867957	-0.655847
H	1.276105	-1.145031	-1.391008
H	1.990133	-2.757257	0.293175

PC:
$E_{B3LYP-D3}$= -3015.9852814
E_{M06-2X}= -3015.7641176

C	0.000000	0.000000	0.000000
C	0.000000	0.000000	1.380100
C	1.179200	0.000000	2.142400
C	2.471100	0.000800	1.644000
C	2.870700	-0.002700	0.285400
S	4.538000	-0.015100	-0.157300
As	1.532400	-0.007000	-1.079700
C	4.144100	-3.058600	1.085800
O	3.852300	-3.483000	0.045300
O	4.427900	-2.731400	2.164200
H	-0.956200	0.000500	-0.515400
H	-0.946300	0.000700	1.914500
H	1.073200	-0.000500	3.222600
H	3.281000	-0.001600	2.367600

RP(B):

RC:

$E_{B3LYP-D3}$= -3015.9369464
E_{M06-2X}= -3015.7115091

C	0.000000	0.000000	0.000000
C	0.000000	0.000000	1.441660
C	1.161277	0.000000	2.145287
C	2.417855	-0.000128	1.479597
C	2.408576	-0.000373	0.133592
O	1.267388	-0.000281	-0.594013
O	-0.961927	0.000185	-0.735581
C	5.850189	-0.048213	2.330997
S	6.082350	-0.035214	0.724237
As	5.574691	-0.061375	4.027358
H	3.299013	-0.001660	-0.476743
H	3.354938	-0.002426	2.016876
H	1.142626	-0.000441	3.227862
H	-0.970295	-0.000190	1.914968

TS1:

$E_{B3LYP-D3}$= -3015.8944764
E_{M06-2X}= -3015.6734747

C	-0.019383	0.081218	-0.023177
C	0.039830	0.003493	1.349214
C	1.299698	0.183063	1.925741
O	2.192683	1.044543	1.310715
C	2.211463	1.101541	-0.058194
C	1.185273	0.313278	-0.713535
C	2.286125	-1.784264	1.587655
As	2.556206	-1.846002	-0.164037
O	3.049124	1.807716	-0.593403
S	2.446966	-2.460233	3.092540
H	1.467188	0.161094	2.989454
H	-0.779228	-0.356252	1.953002
H	-0.912973	-0.185611	-0.569529
H	1.212232	0.339311	-1.792004

INT:

$E_{B3LYP-D3}$= -3015.9381254

E_{M06-2X}= -3015.7290337

C	0.004894	-0.032667	-0.011079
C	0.023129	-0.007115	1.493053
O	1.425917	0.015440	1.944182
C	2.198999	-0.976747	1.452120
C	1.522086	-1.834695	0.432910
C	0.774772	-0.982598	-0.537154
C	-0.677163	-1.257671	2.067392
S	-2.094575	-1.029341	2.969043
O	3.343227	-1.102168	1.843952
As	0.154835	-2.869978	1.648365
H	-0.406384	0.894031	1.916130
H	-0.608088	0.658555	-0.568209
H	0.873971	-1.162671	-1.598690
H	2.222718	-2.534577	-0.004075

TS2:

$E_{B3LYP-D3}$= -3015.9098277

E_{M06-2X}= -3015.6845351

As	0.088523	0.355623	-0.322439
C	0.059107	-0.007425	1.677844
C	1.656302	-0.008512	1.990286
O	2.305489	-1.000174	1.574975
C	0.932146	-2.247572	0.412604
C	0.684862	-1.358054	-0.709261
C	-0.489942	-1.293899	2.065019
C	0.001932	-2.412133	1.479963
O	2.058441	0.978175	2.616817
S	0.955634	-2.023126	-2.269336
H	1.650925	-3.038108	0.236075
H	-0.171788	-3.400833	1.881225
H	-1.166777	-1.355632	2.908016
H	-0.367990	0.837354	2.203569

PC:
$E_{B3LYP-D3}$= -3015.9850677
E_{M06-2X}= -3015.763173

C	0.000000	0.000000	0.000000
As	0.000000	0.000000	1.878083
C	1.891131	0.000000	2.186412
S	2.491627	0.004708	3.794384
C	1.126509	-0.002851	-0.795728
C	2.430748	-0.006048	-0.269204
C	2.768352	-0.004764	1.070973
C	0.087748	-3.213188	0.039953
O	1.195850	-3.340085	0.353560
O	-1.024824	-3.152703	-0.295043
H	3.827079	-0.011225	1.315393
H	3.251710	-0.013748	-0.979245
H	1.017951	-0.002461	-1.877047
H	-0.972413	-0.004795	-0.483196

$[AsCSe]^{-}$:

$[AsCSe]^{-}$:
$E_{B3LYP-D3}$= -4675.8182166
E_{M06-2X}= -4675.7600328

C	0.000000	0.000000	-0.165656
Se	0.000000	0.000000	1.617557
As	0.000000	0.000000	-1.875501

Product:
$E_{B3LYP-D3}$= -4830.7250208
E_{M06-2X}= -4830.5964749

C	-0.027278	0.048852	0.030499
C	0.354108	-0.525173	1.237318
C	1.426015	-0.131904	2.053810
As	2.532299	1.321083	1.553491
C	1.701583	1.778602	-0.056989
C	0.600858	1.135805	-0.588660
Se	1.787951	-1.050600	3.685037
H	-0.239050	-1.365511	1.585217
H	-0.884148	-0.386532	-0.470316
H	0.193818	1.481622	-1.533259
H	2.126764	2.613664	-0.603427

RP(A):

RC:
$E_{B3LYP-D3}$= -5019.3400833
E_{M06-2X}= -5019.1361551

C	0.000000	0.000000	0.000000
C	0.000000	0.000000	1.360814
C	1.215909	0.000000	2.088290
C	2.367836	-0.000834	1.386116
O	2.396308	0.000074	0.038162
C	1.231505	0.001133	-0.743817
O	1.368278	0.003225	-1.947234
C	3.741460	-0.290781	4.581340
Se	3.756761	1.486291	4.576293
As	3.706788	-2.000383	4.544026
H	3.349774	-0.002009	1.834067
H	1.266659	-0.001301	3.164827
H	-0.940901	-0.000642	1.897473
H	-0.907985	-0.000824	-0.583632

TS1:
$E_{B3LYP-D3}$= -5019.2994713
E_{M06-2X}= -5019.0998474

C	0.033271	0.125847	0.004159
C	0.133900	0.170805	1.429687
O	1.400400	-0.140032	1.959582
C	2.058820	-1.226680	1.424282
C	1.504748	-1.728785	0.196074
C	0.734759	-0.872486	-0.612279
As	-1.195166	-1.527524	2.185096
C	-0.191865	-2.860631	1.584401
Se	0.221041	-4.596249	1.477441
O	3.052153	-1.632562	2.009357
H	-0.261933	1.026491	1.958931
H	-0.700586	0.734826	-0.502193
H	0.614878	-1.084862	-1.665899
H	1.981035	-2.606547	-0.208621

INT:
$E_{B3LYP-D3}$= -5019.3363812
E_{M06-2X}= -5019.1501606

C	-0.143210	-0.065398	-0.004363
C	0.063877	-0.027201	1.533165
C	1.568613	-0.067139	1.789990
O	2.149096	-1.232059	1.461641
C	1.234549	-2.250264	0.920680
As	0.531549	-1.545641	-0.883912
C	-0.503260	-1.260167	2.208191
C	0.078866	-2.402209	1.853286
O	2.218278	0.836428	2.266874
Se	-1.005835	1.382065	-0.788799
H	1.842102	-3.138271	0.798451
H	-0.241198	-3.383001	2.174985
H	-1.333018	-1.168431	2.892336
H	-0.327310	0.896434	1.943968

TS2:
$E_{B3LYP-D3}$= -5019.3119678
E_{M06-2X}= -5019.1121035

C	0.229754	0.061107	0.019869
C	-0.100930	0.128531	1.425096
O	1.864521	0.010699	2.095842
C	2.380656	-1.049394	1.637263
C	1.441794	-1.936478	0.636895
C	0.971781	-0.974260	-0.396096
As	-0.844344	-1.385573	2.333753
C	0.356498	-2.558102	1.479330
Se	0.364457	-4.374879	1.774354
O	3.514138	-1.487229	1.833115
H	-0.343188	1.105942	1.824991
H	-0.050854	0.878604	-0.631733
H	1.341698	-1.062145	-1.407433
H	2.095335	-2.698469	0.228199

PC:
$E_{B3LYP-D3}$= -5019.3950758
E_{M06-2X}= -5019.197679

C	-0.217279	-0.711621	-0.172098
As	-0.143681	-0.123498	1.605969
C	1.734511	0.119437	1.763296
Se	2.456390	0.737278	3.396981
C	0.875165	-0.864715	-1.002533
C	2.189544	-0.596420	-0.593218
C	2.573395	-0.152869	0.663780
C	2.859630	3.284432	1.079421
O	3.927335	2.935827	0.781147
O	1.804773	3.708201	1.314427
H	-1.202165	-0.942151	-0.567754
H	0.725063	-1.208782	-2.022401
H	2.979944	-0.747634	-1.321124
H	3.634680	0.010883	0.824078

RP(B):

RC:
$E_{B3LYP-D3}$= -5019.3945498
E_{M06-2X}= -5019.1379544

C	0.000000	0.000000	0.000000
C	0.000000	0.000000	1.438662
C	1.164822	0.000000	2.142270
C	2.416161	-0.001104	1.477622
C	2.410674	-0.002774	0.128534
O	1.271640	-0.001787	-0.592761
O	-0.959332	0.001725	-0.739320
C	5.856317	-0.281910	0.603027
Se	5.855022	1.495117	0.587696
As	5.811251	-1.991663	0.612995
H	3.301844	-0.004757	-0.480314
H	3.363898	-0.002340	1.990768
H	1.137695	0.000213	3.225128
H	-0.969019	-0.000111	1.914135

TS1:

$E_{B3LYP-D3}$= -5019.3004465
E_{M06-2X}= -5019.1025213

C	-0.016341	0.075576	-0.022737
C	0.058397	-0.024795	1.348179
C	1.320939	0.163843	1.916645
O	2.201281	1.033197	1.303213
C	2.207605	1.112840	-0.067227
C	1.166403	0.351038	-0.725787
C	2.296798	-1.784653	1.551290
As	2.592585	-1.846126	-0.182390
O	3.047852	1.819844	-0.595970
Se	2.453294	-2.560496	3.196528
H	1.494313	0.135825	2.979317
H	-0.747145	-0.410422	1.953605
H	-0.912744	-0.198270	-0.561195
H	1.181497	0.390514	-1.803984

INT:

$E_{B3LYP-D3}$= -5019.3455745
E_{M06-2X}= -5019.1595012

C	0.004984	-0.037960	-0.009135
C	0.023709	-0.015502	1.496503
O	1.430943	0.012883	1.942524
C	2.205534	-0.975900	1.448877
C	1.528637	-1.835716	0.430050
C	0.779007	-0.982639	-0.537890
C	-0.663686	-1.269838	2.063235
Se	-2.219350	-1.034696	3.048597
O	3.350726	-1.100205	1.837823
As	0.159495	-2.869322	1.646347
H	-0.402958	0.887230	1.919097
H	-0.610671	0.653437	-0.562943
H	0.880349	-1.159309	-1.599658
H	2.229755	-2.534572	-0.007813

TS2:
$E_{B3LYP-D3}$= -5019.3192984
E_{M06-2X}= -5019.1172903

As	0.079015	0.357238	-0.307158
C	0.064133	-0.003343	1.694088
C	1.657697	-0.004147	2.001178
O	2.304856	-0.998150	1.586111
C	0.935677	-2.238976	0.417105
C	0.680912	-1.343513	-0.688401
C	-0.489505	-1.293687	2.073117
C	0.000451	-2.408838	1.484922
O	2.066341	0.983997	2.621519
Se	0.952863	-2.084308	-2.391751
H	1.663140	-3.022730	0.247413
H	-0.183102	-3.400799	1.873196
H	-1.171990	-1.356540	2.911325
H	-0.363626	0.839482	2.222692

PC:
$E_{B3LYP-D3}$= -5019.3945498
E_{M06-2X}= -5019.1965528

C	0.000000	0.000000	0.000000
As	0.000000	0.000000	1.877383
C	1.880473	0.000000	2.177038
Se	2.538776	0.000943	3.939155
C	1.128730	-0.001143	-0.793756
C	2.431281	-0.002543	-0.268126
C	2.764398	-0.001737	1.075923
C	0.135575	-3.224453	0.027754
O	1.253137	-3.327287	0.315165
O	-0.985600	-3.183831	-0.280353
H	3.822831	-0.006619	1.320270
H	3.253460	-0.008519	-0.976333
H	1.020378	-0.000735	-1.875109
H	-0.971544	-0.005069	-0.484635

MeCP:

MeCP:

$E_{B3LYP-D3}$= -419.388393
E_{M06-2X}= -419.3084752

C	0.000000	-0.000000	0.067538
P	0.000000	-0.000000	1.615063
C	0.000000	-0.000000	-1.388016
H	-1.020331	0.000000	-1.777831
H	0.510166	0.883633	-1.777831
H	0.510166	-0.883633	-1.777831
XX	1.000000	0.000000	0.067538

Product:

$E_{B3LYP-D3}$= -574.321303
E_{M06-2X}= -574.1673586

C	-0.001977	-0.000730	-0.002383
P	0.002298	-0.000206	1.734006
C	1.724789	0.000175	2.082803
C	2.155790	0.001883	3.532616
C	1.128613	-0.000879	-0.807395
C	2.417612	-0.000839	-0.286850
C	2.688314	-0.000416	1.081129
H	3.730999	-0.000575	1.383074
H	3.253994	-0.001305	-0.973349
H	1.005973	-0.001171	-1.884019
H	-0.974284	-0.000745	-0.481256
H	3.242256	-0.010969	3.619268
H	1.788933	0.886683	4.055913
H	1.767044	-0.867697	4.065374

RP(A):

RC:
$E_{B3LYP-D3}$= -762.9088507
E_{M06-2X}= -762.6854111

C	-0.962169	1.158236	-0.343077
C	-1.667709	0.210718	-1.165105
C	-2.107274	-0.965626	-0.655709
C	-1.874939	-1.291400	0.711686
C	-1.221187	-0.394827	1.472789
O	-0.788117	0.786508	0.992976
O	-0.510000	2.233238	-0.674428
C	2.246561	-0.146832	0.004209
C	2.568739	1.187158	0.482377
P	1.878032	-1.560401	-0.506204
H	-2.632424	-1.667162	-1.289512
H	-2.203635	-2.224434	1.139972
H	-0.982405	-0.520245	2.517801
H	-1.817042	0.488509	-2.196848
H	3.483510	1.559229	0.015459
H	1.757107	1.880684	0.250090
H	2.717220	1.186899	1.564713

TS1:
$E_{B3LYP-D3}$= -762.870612
E_{M06-2X}= -762.6510769

C	0.032880	0.229916	-0.214078
C	-0.059040	-0.311229	1.134718
O	1.128558	-0.316253	1.827430
C	2.277065	0.075667	1.168509
C	2.195049	1.097180	0.215274
C	1.015293	1.196130	-0.481874
O	-1.050197	-0.741939	1.681751
C	0.974150	-1.672865	-0.889208
C	-0.130381	-2.201199	-1.711885
P	2.446171	-1.864535	-0.293703
H	-0.893352	0.219628	-0.767189
H	0.899980	1.875673	-1.313554
H	3.084180	1.648249	-0.050746
H	3.142124	-0.062780	1.798167
H	0.122938	-3.174099	-2.133620
H	-1.035626	-2.314160	-1.110890
H	-0.370459	-1.518243	-2.529811

INT:
$E_{B3LYP\text{-}D3}$= -762.917778
$E_{M06\text{-}2X}$= -762.7076308

C	0.103368	-0.056202	-0.300588
C	-0.029067	-0.212744	1.218191
O	1.158397	-0.315299	1.839184
C	2.324159	-0.227029	0.944665
C	2.198017	1.018550	0.117498
C	1.028115	1.131734	-0.499772
O	-1.067233	-0.240768	1.831069
C	0.744897	-1.362020	-0.793396
C	-0.069314	-2.187124	-1.734203
P	2.267512	-1.769378	-0.206732
H	-0.883631	0.092586	-0.724968
H	0.707309	1.974534	-1.091998
H	3.006002	1.732337	0.065348
H	3.184233	-0.248431	1.601984
H	0.439710	-3.100759	-2.035715
H	-1.022517	-2.458358	-1.271636
H	-0.310625	-1.606492	-2.629638

TS2:
$E_{B3LYP\text{-}D3}$= -762.891334
$E_{M06\text{-}2X}$= -762.6682069

C	0.030907	0.162805	0.179692
P	0.273186	-0.096196	1.963308
C	1.973407	0.014649	1.897680
C	2.839273	-0.235893	3.091085
C	0.750370	1.184461	-0.517887
C	2.082186	1.215717	-0.308086
C	2.649447	0.217370	0.602853
O	1.102916	-1.426756	-0.493257
C	2.314597	-1.258086	-0.229534
O	3.304133	-1.930829	-0.449396
H	3.729831	0.246485	0.678536
H	2.748678	1.824172	-0.901720
H	0.261669	1.766925	-1.285700
H	-0.927671	-0.139716	-0.222337
H	3.501754	-1.085461	2.904497
H	3.481643	0.629094	3.275647
H	2.259747	-0.442055	3.989139

PC:
$E_{B3LYP-D3}$= -762.9913289
E_{M06-2X}= -762.7677953

C	0.241829	1.388362	-0.834600
C	0.453537	1.710390	0.496758
C	-0.276554	1.135389	1.534525
C	-1.276953	0.191642	1.328755
C	-1.667884	-0.291526	0.082858
C	-2.762121	-1.330058	0.013452
P	-0.935390	0.244901	-1.413614
C	2.481541	-0.962770	0.069465
O	1.710668	-1.710465	0.507356
O	3.262083	-0.222740	-0.366707
H	-1.786308	-0.198515	2.204679
H	-0.055041	1.434977	2.550372
H	1.218126	2.436667	0.745765
H	0.852314	1.876731	-1.584813
H	-2.459978	-2.241108	0.534336
H	-3.672341	-0.967139	0.494838
H	-3.008308	-1.598057	-1.013445

RP(B):

RC
$E_{B3LYP-D3}$= -762.9918677
E_{M06-2X}= -762.6854108

C	-0.962169	1.158236	-0.343077
C	-1.667709	0.210718	-1.165105
C	-2.107274	-0.965626	-0.655709
C	-1.874939	-1.291400	0.711686
C	-1.221187	-0.394827	1.472789
O	-0.788117	0.786508	0.992976
O	-0.510000	2.233238	-0.674428
C	2.246561	-0.146832	0.004209
C	2.568739	1.187158	0.482377
P	1.878032	-1.560401	-0.506204
H	-2.632424	-1.667162	-1.289512
H	-2.203635	-2.224434	1.139972
H	-0.982405	-0.520245	2.517801
H	-1.817042	0.488509	-2.196848
H	3.483510	1.559229	0.015459
H	1.757107	1.880684	0.250090
H	2.717220	1.186899	1.564713

TS1:
$E_{B3LYP-D3}$= -762.872570
E_{M06-2X}= -762.6524275

C	-0.099961	-0.047089	0.128181
C	0.056747	0.088780	1.510732
O	1.303508	-0.093746	2.064768
C	2.133109	-1.047815	1.522551
C	1.635353	-1.661928	0.302582
C	0.725743	-0.958940	-0.491813
O	3.185052	-1.279426	2.079381
C	-0.907909	-1.692063	2.215978
C	-2.096913	-1.135499	2.895009
P	-0.045228	-2.979239	1.833204
H	-0.505889	0.809846	2.083540
H	-0.942713	0.412678	-0.364115
H	0.586113	-1.233893	-1.527476
H	2.259426	-2.440160	-0.108470
H	-2.731535	-1.922470	3.301046
H	-1.798118	-0.479575	3.716518
H	-2.689586	-0.531209	2.203684

INT:
$E_{B3LYP-D3}$= -762.922522
E_{M06-2X}= =-762.7127609

C	-0.098700	-0.046429	0.045996
C	-0.124335	-0.048881	1.554277
O	1.270931	-0.021650	2.032366
C	2.031845	-1.026392	1.556661
C	1.308348	-1.912648	0.561266
C	0.657318	-1.015094	-0.457693
O	3.174787	-1.167618	1.922180
C	-0.776146	-1.333170	2.074058
C	-1.998064	-1.182461	2.916427
P	-0.038518	-2.781899	1.651084
H	-0.572176	0.840463	1.984562
H	-0.643044	0.693002	-0.519457
H	0.834206	-1.179678	-1.510016
H	1.993317	-2.643852	0.151041
H	-2.412749	-2.139491	3.227098
H	-1.770807	-0.592186	3.808848
H	-2.769015	-0.633766	2.366601

TS2:

$E_{B3LYP-D3}$= -762.8977121
E_{M06-2X}= -762.6752691

C	0.205388	0.077435	0.143879
P	0.367379	0.071677	1.993046
C	2.063430	0.115388	2.033059
C	2.864347	-0.010466	3.296959
C	1.056503	1.013963	-0.584966
C	2.371484	1.016731	-0.285923
C	2.780062	0.146267	0.776302
C	0.812349	-1.449894	-0.226460
O	-0.025339	-2.233041	-0.647152
O	2.045381	-1.589024	-0.032451
H	3.816149	-0.169242	0.796437
H	3.117060	1.517975	-0.884478
H	0.653356	1.560188	-1.426196
H	-0.831397	0.068462	-0.166396
H	3.556908	0.826630	3.405789
H	2.225008	-0.042825	4.177648
H	3.462794	-0.925185	3.272886

PC:

$E_{B3LYP-D3}$= -762.9918677
E_{M06-2X}= -762.7682873

C	0.241829	1.388362	-0.834600
C	0.453537	1.710390	0.496758
C	-0.276554	1.135389	1.534525
C	-1.276953	0.191642	1.328755
C	-1.667884	-0.291526	0.082858
C	-2.762121	-1.330058	0.013452
P	-0.935390	0.244901	-1.413614
C	2.481541	-0.962770	0.069465
O	1.710668	-1.710465	0.507356
O	3.262083	-0.222740	-0.366707
H	-1.786308	-0.198515	2.204679
H	-0.055041	1.434977	2.550372
H	1.218126	2.436667	0.745765
H	0.852314	1.876731	-1.584813
H	-2.459978	-2.241108	0.534336
H	-3.672341	-0.967139	0.494838
H	-3.008308	-1.598057	-1.013445
